# Novel Sulfonamide Analogs of Sivelestat as Potent Human Neutrophil Elastase Inhibitors

**DOI:** 10.3389/fchem.2020.00795

**Published:** 2020-09-01

**Authors:** Letizia Crocetti, Maria Paola Giovannoni, Niccolò Cantini, Gabriella Guerrini, Claudia Vergelli, Igor A. Schepetkin, Andrei I. Khlebnikov, Mark T. Quinn

**Affiliations:** ^1^Neurofarba, Pharmaceutical and Nutraceutical Section, University of Florence, Sesto Fiorentino, Italy; ^2^Department of Microbiology and Immunology, Montana State University, Bozeman, MT, United States; ^3^Kizhner Research Center, Tomsk Polytechnic University, Tomsk, Russia

**Keywords:** human neutrophil elastase, inhibitor, Sivelestat, stability, molecular modeling

## Abstract

Human neutrophil elastase (HNE) is involved in a number of essential physiological processes and has been identified as a potential therapeutic target for treating acute and chronic lung injury. Nevertheless, only one drug, Sivelestat, has been approved for clinical use and just in Japan and the Republic of Korea. Thus, there is an urgent need for the development of low-molecular-weight synthetic HNE inhibitors, and we have developed a wide variety of HNE inhibitors with various chemical scaffolds. We hypothesized that substitution of the active fragment of Sivelestat into these HNE inhibitor scaffolds could modulate their inhibitory activity, potentially resulting in higher efficacy and/or improved chemical stability. Here, we report the synthesis, biological evaluation, and molecular modeling studies of novel compounds substituted with the 4-(sulfamoyl)phenyl pivalate fragment necessary for Sivelestat activity. Many of these compounds were potent HNE inhibitors with activity in the nanomolar range (IC_50_ = 19–30 nM for compounds **3a**, **3b**, **3f**, **3g**, and **9a**), confirming that the 4-(sulfamoyl)phenyl pivalate fragment could substitute for the N-CO group at position 1 and offer a different point of attack for Ser195. Results of molecular docking of the these pivaloyl-containing compounds into the HNE binding site supported the mechanism of inhibitory activity involving a nucleophilic attack of Ser195 from the catalytic triad onto the pivaloyl carbonyl group. Furthermore, some compounds (e.g., **3a** and **3f**) had a relatively good stability in aqueous buffer (t_1/2_ > 9 h). Thus, this novel approach led to the identification of a number of potent HNE inhibitors that could be used as leads for the further development of new therapeutics.

## Introduction

Human neutrophil elastase (HNE) is a multifunctional enzyme involved in the killing of pathogens, regulation of inflammatory processes, and tissue homeostasis. HNE is also involved in chemotaxis and the release of inflammatory mediators through the cleavage of adhesion molecules in cellular junctions (Pham, 2006; Korkmaz et al., 2010). Under physiological conditions, HNE is regulated by a group of endogenous protease inhibitors called “serpins” (Silverman et al., 2001; Heutinck et al., 2010). However, when this balance fails in favor of the proteolytic enzyme, excessive HNE activity can cause tissue damage. Among the pathologies associated with increased HNE activity are acute respiratory distress syndrome (ARDS) and acute lung injury (ALI) (Polverino et al., 2017), chronic obstructive pulmonary disease (Pandey et al., 2017), cystic fibrosis (Kelly et al., 2008; Dittrich et al., 2018), and other disorders with an inflammatory component, such as rheumatoid arthritis (Hilbert et al., 2002; Di Cesare Mannelli et al., 2016), atherosclerosis (Henriksen and Sallenave, 2008; Wen et al., 2018), psoriasis, and dermatitis (Marto et al., 2018). HNE has also been implicated in the progression of non-small cell lung cancer (Lerman and Hammes, 2017; Lerman et al., 2017).

**Figure 1:**
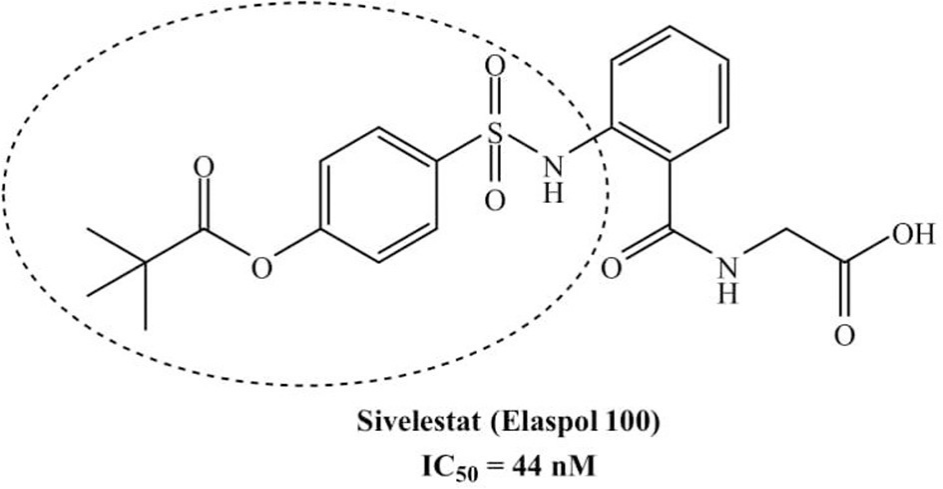
Sivelestat (ONO-5046) claimed by Ono Pharmaceutical (Kawabata et al., 1991).

**Figure 2:**
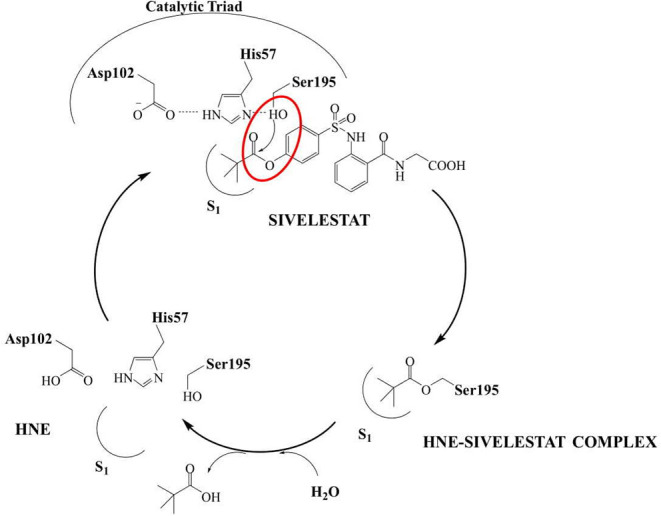
Mechanism of HNE inhibition by Sivelestat.

**Figure 3:**
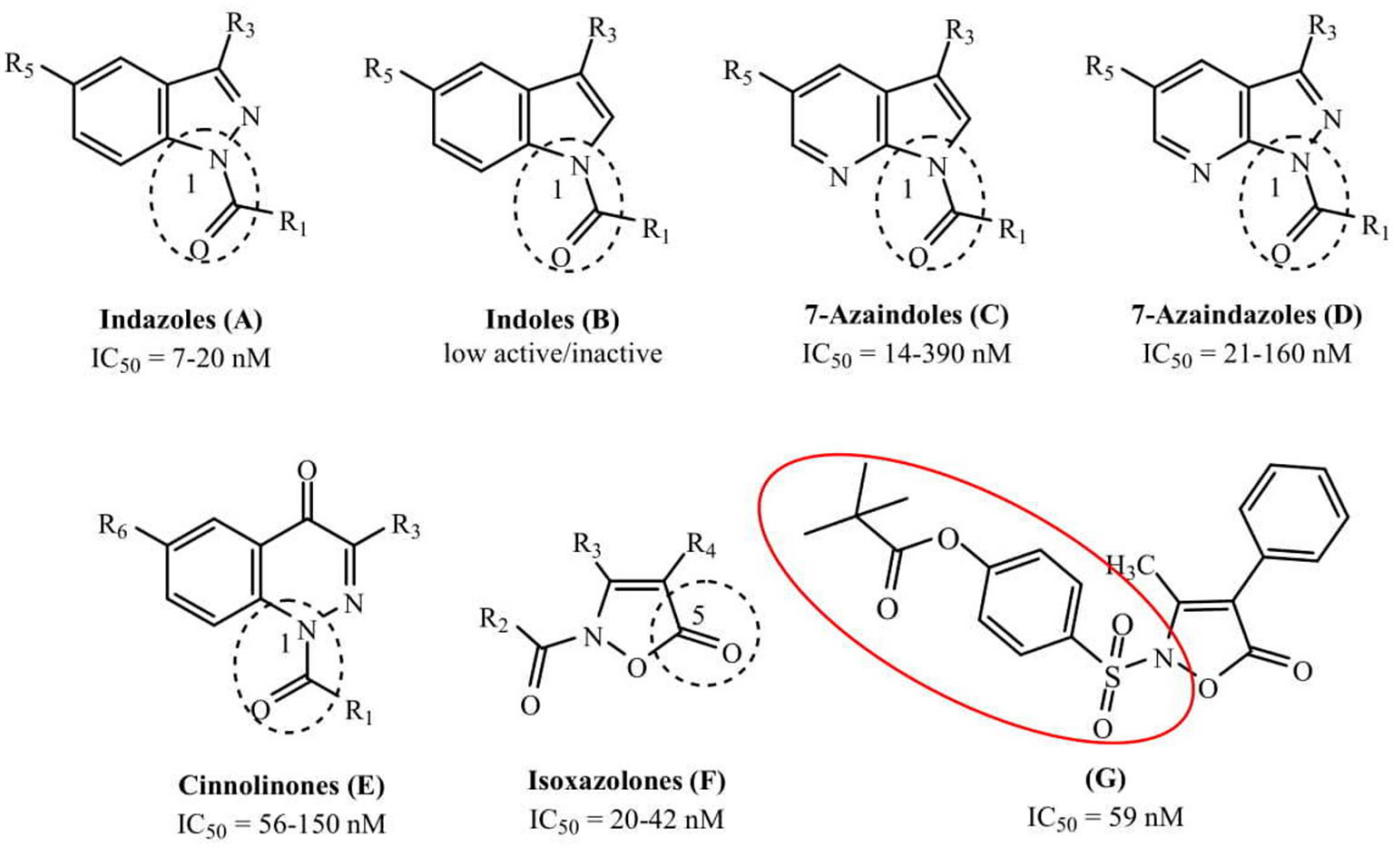
The investigated scaffolds of HNE inhibitors and range of their inhibitory activity. The segmented circle underlines the point of attack by OH group of Ser195 (**G**: **3b**; Giovannoni et al., 2018).

The development of new and selective HNE inhibitors is of great interest, both in the academic and industrial world, for therapeutic development. Despite the discovery of numerous classes of potent HNE inhibitors (Groutas et al., 2011; Von Nussbaum and Li, 2015; Crocetti et al., 2019), only two drugs are currently available on the market: the peptide inhibitor Prolastin® (purified α1-antitrypsin, from Alpha Therapeutic Corp, 2003) and the small molecule Sivelestat. Sivelestat (ONO-5046, Figure 1) developed by Ono Pharmaceutical and marketed in Japan and Korea exclusively as a sodium salt in the injectable formulation Elaspol® 100 for the treatment of ARDS and ALI associated with systemic inflammatory response syndrome and in pediatric surgery to alleviate the inflammatory response induced by cardiopulmonary bypass (Kawabata et al., 1991; Fujii et al., 2010; Inoue et al., 2013). Sivelestat acts as acyl-enzyme inhibitor (IC_50_ = 44 nM) (Ohbayashi, 2005), as demonstrated by electrospray ionization mass spectrometry (ESI), which highlighted the formation of a HNE-Sivelestat complex after 0–10 min incubation of the drug with HNE (Figure 2) (Nakayama et al., 2002). We have been working for some time on the design and synthesis of HNE inhibitors with different nitrogen monocyclic and bicyclic scaffolds (Crocetti et al., 2011, 2013, 2016, 2017, 2018; Giovannoni et al., 2016, 2018, 2019; Vergelli et al., 2017). Figure 3 illustrates the compounds already investigated (**A-F**) and the range of activity for each series. Many of these compounds have very potent HNE inhibitory activity (IC_50_ values in the low nanomolar range), and kinetic experiments have characterized our compounds as competitive and pseudo-irreversible acyl-enzyme inhibitors. Furthermore, molecular modeling studies on compound interactions with HNE indicated that Ser195-OH of the catalytic triad attacks the carbonyl group of the N-CO group at position 1 in the bicyclic compounds (**A-E**) and the CO at position 5 in the monocyclic derivatives **F** (Vergelli et al., 2017; Giovannoni et al., 2018).

Since the first patent in which Sivelestat was reported (Ono Pharmaceutical Co. and LTD., 1989), followed by the publication of Kawabata and coworkers (Kawabata et al., 1991), few articles have appeared in the literature describing Sivelestat analogs and (or) derivatives. The patent filed in 2002 (Macias, 2002) reports the introduction of substituents on the benzoyl moiety of Sivelestat, whereas Hwang et al. (2015) performed structural modification of Sivelestat by replacing the glycine moiety with an oxime group and obtained a slightly more potent compound. Recently, we synthesized the isoxazolone derivative **G** (Giovannoni et al., 2018) (Figure 3) containing the 4-(sulfonyl)phenyl pivalate fragment bound to the aniline nitrogen of Sivelestat. This compound exhibited high HNE inhibitory activity (IC_50_ = 59 nM) and excellent chemical stability in aqueous buffer (data not shown), which was improved over that of our previously published HNE inhibitors.

In the present studies, we expanded our strategy to evaluate how addition of the 4-(sulfonyl)phenyl pivalate fragment could modulate inhibitory activity of other HNE inhibitor scaffolds to evaluate if an additive effect would occur or whether these modifications could improve chemical stability of these new compounds. Therefore, we selected a number of compounds from our HNE inhibitor library belonging to the series **A-E** shown in Figure 3 and inserted the active 4-(sulfamoyl)phenyl pivalate fragment of Sivelestat into our scaffolds while leaving/maintaining the best substituents at positions 3 and 5/6. In most of these new bicyclic compounds, the carbonyl group of the N-CO function at position 1 was replaced with the Sivelestat pivalate fragment. In a few compounds, the N-CO function of our original compounds was left unchanged, and the active fragment of Sivelestat was inserted into a different position in order to produce two possible points of interaction with Ser195.

## Materials and Methods

### Chemistry

All melting points were determined on a Büchi apparatus (New Castle, DE) and are uncorrected. Extracts were dried over Na_2_SO_4_, and the solvents were removed under vacuum. Merck F-254 commercial plates (Merck, Durham, NC) were used for analytical TLC to follow the course of reactions. Silica gel 60 (Merck 70–230 mesh, Merck, Durham, NC) was used for column chromatography. ^1^H NMR and ^13^C NMR spectra were recorded on an Avance 400 instrument (Bruker Biospin Version 002 with SGU, Bruker Inc., Billerica, MA). Chemical shifts (δ) are reported in ppm to the nearest 0.01 ppm using the solvent as an internal standard. Coupling constants (J values) are given in Hz and were calculated using TopSpin 1.3 software (Nicolet Instrument Corp., Madison, WI) and are rounded to the nearest 0.1 Hz. Mass spectra (m/z) were recorded on an ESI-TOF mass spectrometer (Brucker Micro TOF, Bruker Inc., Billerica, MA), and reported mass values are within the error limits of ±5 ppm mass units. Microanalyses indicated by the symbols of the elements or functions were performed with a PerkinElmer 260 elemental analyzer (PerkinElmer, Inc., Waltham, MA) for C, H, and N, and the results were within ±0.4% of the theoretical values, unless otherwise stated. Reagents and starting material were commercially available.

### Experimental Section

#### General Procedure for Compounds (3a-c)

To a suspension of the substrate **1a-c** (1a: Shahidul et al., 2006; 1b: DeGraw and Goodman, 1964; 1c: Yuen et al., 2013) (0.43 mmol) in 10 mL of anhydrous THF, 0.86 mmol of sodium hydride (60% dispersion in mineral oil) was added while stirring. After 30 min, 0.56 mmol of 4-(chlorosulfonyl)phenyl pivalate **2** (Hwang et al., 2015) was added, and the mixture was stirred at room temperature overnight. After evaporation of the solvent *in vacuo*, the residue was diluted with ice-cold water (10 mL), neutralized with HCl 6N, and extracted with ethyl acetate (3 × 15 mL). The organic phase was dried over sodium sulfate, and the solvent was evaporated *in vacuo* to obtain the final compounds **3a-c**, which were purified by flash column chromatography using cyclohexane/ethyl acetate 4:1 (for **3a**) and 6:1 (for **3c**) or hexane/ethyl acetate 5:2 for **3b** as eluents.

##### Ethyl 1-{[4-(pivaloyloxy)phenyl] sulfonyl}-1H-indole-3 -carboxylate (3a)

Yield = 15%; mp = 118–121°C (EtOH). ^1^H-NMR (400 MHz, CDCl_3_) δ 8.28 (s, 1H, Ar), 8.16 (dd, 1H, Ar, *J* = 2.2 Hz and *J* = 6.6 Hz), 7.99 (m, 3H, Ar), 7.38 (m, 2H, Ar), 7.22 (d, 2H, Ar, *J* = 8.8 Hz), 4.41 (q, 2H, *CH*_2_CH_3_, *J* = 7.1 Hz), 1.45 (t, 3H, CH_2_*CH*_3_, *J* = 7.1 Hz), 1.33 (s, 9H, C(CH_3_)_3_). ^13^C-NMR (100 MHz, CDCl_3_) δ 175.95 (C), 163.59 (C), 155.74 (C), 134.83 (C), 134.48 (C), 131.80 (CH), 128.82 (CH), 127.88 (C), 125.52 (CH), 124.56 (CH), 122.77 (CH), 122.35 (CH), 114.26 (C), 113.23 (CH), 60.61 (CH_2_), 39.27 (C), 26.94 (CH_3_), 14.42 (CH_3_). IR (ν) = 1,753 cm^−1^ (CO), 1,718 cm^−1^ (CO). ESI-MS calcd. for C_22_H_23_NO_6_S, 429.49; found: m/z 430.13 [M + H]^+^. Anal. C_22_H_23_NO_6_S (C, H, N).

##### Ethyl 5-nitro-1-{[4-(pivaloyloxy)pheny]sulfonyl}-1H-indole-3-carboxylate (3b)

Yield = 25%; mp = 170-173°C (EtOH). ^1^H-NMR (400 MHz, CDCl_3_) δ 9.04 (d, 1H, Ar, *J* = 2.4 Hz), 8.39 (s, 1H, Ar), 8.27 (dd, 1H, Ar, *J* = 2.4 Hz, and *J* = 9.2 Hz), 8.09 (d, 1H, Ar, *J* = 9.2 Hz), 8.01 (d, 2H, Ar, *J* = 8.8 Hz), 7.27 (d, 2H, Ar, *J* = 8.8 Hz), 4.46 (q, 2H, *CH*_2_CH_3_, *J* = 7.2 Hz), 1.46 (t, 3H, CH_2_*CH*_3_, *J* = 7.2 Hz); 1.33 (s, 9H, C(CH_3_)_3_). ^13^C-NMR (100 MHz, CDCl_3_) δ 175.88 (C), 162.53 (C), 156.32 (C), 145.15 (C), 137.49 (C), 134.16 (CH), 133.76 (C), 128.97 (CH), 127.91 (C), 123.16 (C), 120.69 (CH), 118.89 (CH), 114.90 (C), 113.67 (CH), 61.19 (CH_2_), 29.70 (C), 26.91 (CH_3_), 14.38 (CH_3_). IR (ν) = 1,747 cm^−1^ (CO), 1,712 cm^−1^ (CO), 1,517, and 1,377 cm^−1^ (NO_2_). ESI-MS calcd. for C_22_H_22_N_2_O_8_S, 474.48; found: m/z 475.11 [M + H]^+^. Anal. C_22_H_22_N_2_O_8_S (C, H, N).

##### 4-[(3-Cyano-1H-indol-1-yl)sulfonyl]phenyl pivalate (3c)

Yield = 14%; mp = 133–136°C (EtOH). ^1^H-NMR (400 MHz, CDCl_3_) δ 8.11 (s, 1H, Ar), 8.0 (m, 3H, Ar), 7.72 (d, 1H, Ar, *J* = 7.6 Hz), 7.47 (t, 1H, Ar, *J* = 7.6 Hz), 7.41 (t, 1H, Ar, *J* = 7.6 Hz), 7.26 (d, 2H, Ar, *J* = 8.8 Hz), 1.34 (s, 9H, C(CH_3_)_3_). ^13^C-NMR (100 MHz, CDCl_3_) δ 156.13 (C), 133.87 (C), 133.64 (C), 132.98 (CH), 128.94 (CH), 128.38 (C), 126.73 (CH), 125.0 (CH), 123.01 (CH), 120.45 (CH), 113.69 (CH), 113.31 (C), 94.16 (C), 39.31 (C), 26.93 (CH_3_). IR (ν) = 2,231 cm^−1^ (CN), 1,743 cm^−1^ (CO). ESI-MS calcd. for C_20_H_18_N_2_O_4_S, 382.43; found: m/z 383.10 [M + H]^+^. Anal. C_20_H_18_N_2_O_4_S (C, H, N).

#### General Procedure for Compounds (3d-f)

To a cooled (0°C) suspension of the appropriate substrate **1d-f** (1d: Alaime et al., 2018; 1e: Crocetti et al., 2013; 1f: Crocetti et al., 2011) (0.32 mmol) in anhydrous CH_2_Cl_2_ (1–2 mL), 0.64 mmol of Et_3_N and 0.96 mmol of 4-(chlorosulfonyl)phenyl pivalate **2** (Hwang et al., 2015) were added. The solution was stirred at 0°C for 2 h and then for 2 h at room temperature. The organic solvent was evaporated under vacuum. After dilution with ice-cold water, the precipitate was filtered and washed with water (10–20 mL) to obtain the final compounds **3d-f**, which were purified by crystallization from ethanol.

##### 4-[(3-Cyano-1H-indazol-1-yl)sulfonyl]phenyl pivalate (3d)

Yield = 69%; mp = 155–158°C (EtOH). ^1^H-NMR (400 MHz, DMSO-d_6_) δ 8.28 (d, 1H, Ar, *J* = 7.2 Hz), 8.17 (d, 2H, Ar, *J* = 6.4 Hz), 7.99 (d, 1H, Ar, *J* = 6.4 Hz), 7.84 (m, 1H, Ar), 7.61 (m, 1H, Ar), 7.44 (d, 2H, Ar, *J* = 6.4 Hz), 1.25 (s, 9H, C(CH_3_)_3_). ^13^C-NMR (100 MHz, DMSO-d_6_) δ 176.02 (C), 156.71 (C), 139.79 (C), 132.74 (C), 131.98 (CH), 130.37 (CH), 126.99 (CH), 125.95 (C), 125.33 (C), 124.27 (CH), 120.92 (CH), 113.62 (CH), 112.17 (C), 39.35 (C), 26.95 (CH_3_). IR (ν) = 2,247 cm^−1^ (CN), 1,753 cm^−1^ (CO). ESI-MS calcd. for C_19_H_17_N_3_O_4_S, 383.42; found: m/z 384.10 [M + H]^+^. Anal. C_19_H_17_N_3_O_4_S (C, H, N).

##### 4-[(3-Cyano-5-nitro-1H-indazol-1-yl)sulfonyl]phenyl pivalate (3e)

Yield = 44%; mp = 183–186°C (EtOH). ^1^H-NMR (400 MHz, CDCl_3_) δ 8.81 (d, 1H, Ar, *J* = 2.0 Hz), 8.57 (dd, 1H, Ar, *J* = 2.2 Hz and *J* = 9.4 Hz), 8.45 (d, 1H, Ar, *J* = 9.2 Hz), 8.14 (d, 2H, Ar, *J* = 8.8 Hz), 7.33 (d, 2H, Ar, *J* = 8.8 Hz), 1.36 (s, 9H, C(CH_3_)_3_). ^13^C-NMR (100 MHz, CDCl_3_) δ 176.02 (C), 157.08 (C), 145.73 (C), 141.85 (C), 132.30 (C), 130.28 (CH), 126. 99 (C), 125.40 (CH), 125.06 (C), 123.29 (CH), 116.95 (CH), 114.61 (CH), 110.38 (C), 39.36 (C), 26.93 (CH_3_). IR (ν) = 2,245 cm^−1^ (CN), 1,751 cm^−1^ (CO), 1,531 and 1,377 cm^−1^ (NO_2_). ESI-MS calcd. for C_19_H_17_N_4_O_6_S, 428.42; found: m/z 429.08 [M + H]^+^. Anal. C_19_H_17_N_4_O_6_S (C, H, N).

##### Ethyl 1-{[4-(pivaloyloxy)phenyl]sulfonyl}-1H-indazole-3-carboxylate (3f)

Yield = 44%; mp = 132–135°C (EtOH). ^1^H-NMR (400 MHz, CDCl_3_) δ 8.23 (m, 2H, Ar), 8.10 (d, 2H, Ar, *J* = 8.8 Hz), 7.62 (t, 1H, Ar, *J* = 7.6 Hz), 7.45 (t, 1H, Ar, *J* = 7.4 Hz), 7.22 (d, 2H, Ar, *J* = 8.8 Hz), 4.52 (q, 2H, *CH*_2_CH_3_, *J* = 7.1 Hz), 1.48 (t, 3H, CH_2_*CH*_3_, *J* = 7.1 Hz), 1.33 (s, 9H, C(CH_3_)_3_). ^13^C-NMR (100 MHz, CDCl_3_) δ 176.03 (C), 161.31 (C), 155.95 (C), 142.26 (C), 141.18 (C), 133.94 (C), 129.69 (CH), 129.65 (CH), 125.46 (CH), 124.43 (C), 122.79 (CH), 122.68 (CH), 112.99 (CH), 61.84 (CH_2_), 39.26 (C), 26.94 (CH_3_), 14.32 (CH_3_). IR (ν) = 1,745 cm^−1^ (CO), 1,732 cm^−1^ (CO). ESI-MS calcd. for C_21_H_22_N_2_O_6_S, 430.48; found: m/z 431.12 [M + H]^+^. Anal. C_21_H_22_N_2_O_6_S (C, H, N).

#### Procedure for Ethyl 5-nitro-1-{[4-(pivaloyloxy)phenyl]sulfonyl}-1H-indazole-3-carboxylate (3g)

To a suspension of intermediate **1g** (Crocetti et al., 2013) (0.64 mmol) in 5 mL of anhydrous pyridine, 1.92 mmol of 4-(chlorosulfonyl)phenyl pivalate **2** (Hwang et al., 2015) was added. The mixture was stirred at room temperature for 4 h. The solvent was concentrated *in vacuo* to obtain the final compound **3g**, which was purified by crystallization from ethanol. Yield = 72%; mp = 185–188°C (EtOH). ^1^H-NMR (400 MHz, DMSO-d_6_) δ 8.87 (s, 1H, Ar), 8.54 (d, 1H, Ar, *J* = 9.2 Hz), 8.43 (d, 1H, Ar, *J* = 9.2 Hz), 8.18 (d, 2H, Ar, *J* = 8.4 Hz), 7.45 (d, 2H, Ar, *J* = 8.4 Hz), 4.46 (q, 2H, *CH*_2_CH_3_, *J* = 7.0 Hz), 1.38 (t, 3H, CH_2_*CH*_3_, *J* = 7.0 Hz), 1.25 (s, 9H, C(CH_3_)_3_). ^13^C-NMR (100 MHz, DMSO-d_6_) δ 176.08 (C), 160.26 (C), 156.80 (C), 145.67 (C), 143.29 (C), 142.85 (C), 132.84 (C), 130.44 (CH), 125.45 (CH), 124.37 (CH), 123.97 (C), 119.46 (C), 114.65 (CH), 62.62 (CH_2_), 39.18 (C), 26.98 (CH_3_), 14.49 (CH_3_). IR (ν) = 1,736 cm^−1^ (CO), 1,735 cm^−1^ (CO), 1,523 and 1,377 cm^−1^ (NO_2_). ESI-MS calcd. for C_21_H_21_N_3_O_8_S, 475.47; found: m/z 476.11 [M + H]^+^. Anal. C_21_H_21_N_3_O_8_S (C, H, N).

#### Procedure for 4-[(3-Amino-1H-indazol-1-yl)sulfonyl]phenyl Pivalate (3h)

1H-Indazol-3-amine **1h** (Lefebvre et al., 2010) (2.03 mmol) was dissolved in 13 mL of dry DMF and triethylamine (20.28 mmol). 4-(chlorosulfonyl)phenyl pivalate **2** (Hwang et al., 2015) (2.13 mmol) dissolved in 4 mL of dry 1,4-dioxane was added to the reaction mixture dropwise at 10°C, and the mixture was stirred at 50°C for 4 h. Ice-cold water was added to the reaction mixture, and the suspension was extracted with ethyl acetate (3 × 15 mL). The organic phase was dried over sodium sulfate, and the solvent was evaporated *in vacuo* to obtain the desired compound **3h**, which was purified by flash column chromatography using cyclohexane/ethyl acetate 2:1 as eluent. Yield = 5%; mp = 189-192°C (EtOH). ^1^H-NMR (400 MHz, DMSO-d_6_) δ 7.96 (d, 1H, Ar, *J* = 8.2 Hz), 7.79 (d, 1H, Ar, *J* = 8.2 Hz), 7.75 (d, 2H, Ar, *J* = 8.8 Hz), 7.59 (t, 1H, Ar, *J* = 7.6 Hz), 7.35 (t, 1H, Ar, *J* = 7.6 Hz), 7.26 (d, 2H, Ar, *J* = 8.8 Hz), 6.55 (exch br s, 2H, NH_2_), 1.24 (s, 9H, C(CH_3_)_3_). ESI-MS calcd. for C_18_H_19_N_3_O_4_S, 373.43; found: m/z 374.11 [M + H]^+^. Anal. C_18_H_19_N_3_O_4_S (C, H, N).

#### General Procedure for Compounds (3i-l and 4)

Compounds **3i-l** and **4** were obtained starting from intermediates **1i-l** (1i: Bahekar et al., 2007; 1j: Crocetti et al., 2018; 1k,l: Schirok et al., 2015) and **3h**, respectively, following the same procedure described for **3d-f** and using the appropriate sulfonyl chloride **2** (Hwang et al., 2015) (for **3i-l**) or m-toluoyl chloride (for **4**) as reagents. After evaporation of organic solvent, ice-cold water was added (20 mL). Compounds **3i,j** and **4** were recovered by extraction with CH_2_Cl_2_ (3 × 15 mL), while compounds **3k**, **l** were recovered by vacuum filtration. The final compounds **3i,j** and **4** were purified by flash column chromatography using cyclohexane/ethyl acetate 4:1 (for **3i**), 5:1 (for **3j**) or toluene/ethyl acetate 95:5 (for **4**) as eluents or by crystallization from ethanol (compounds **3k** and **3l**).

##### 4-[(3-Cyano-1H-pyrrolo[2,3-b]pyridin-1-yl]sulfonyl)phenyl pivalate (3i)

Yield = 22%; mp = 176–179°C (EtOH). ^1^H-NMR (400 MHz, CDCl_3_) δ 8.56 (dd, 1H, Ar, *J* = 1.6 Hz and *J* = 4.8 Hz), 8.33 (d, 2H, Ar, *J* = 7.2 Hz), 8.30 (s, 1H, Ar), 8.07 (dd, 1H, Ar, *J* = 1.6 Hz and *J* = 8.0 Hz), 7.38 (m, 1H, Ar), 7.28 (d, 2H, Ar, *J* = 7.2 Hz), 1.35 (s, 9H, C(CH_3_)_3_). ^13^C-NMR (100 MHz, CDCl_3_) δ 176.08 (C), 156.32 (C), 147.09 (CH), 145.64 (C), 133.66 (C), 133.17 (CH), 130.59 (CH), 129.11 (CH), 122.64 (CH), 121.01 (C), 120.46 (CH), 112.90 (C), 90.67 (C), 39.31 (C), 26.96 (CH_3_). IR (ν) = 2,233 cm^−1^ (CN), 1,755 cm^−1^ (CO). ESI-MS calcd. for C_19_H_17_N_3_O_4_S, 383.42; found: m/z 384.10 [M + H]^+^. Anal. C_19_H_17_N_3_O_4_S (C, H, N).

##### 4-{[3-(3-Methyl-1,2,4-oxadiazol-5-yl)-1H-pyrrolo[2,3-b]pyridin-1-yl]sulfonyl}phenyl pivalate (3j)

Yield = 8%; mp = 164–167°C (EtOH). ^1^H-NMR (400 MHz, CDCl_3_) δ 8.55 (m, 3H, Ar), 8.34 (d, 2H, Ar, *J* = 8.8 Hz), 7.39 (m, 1H, Ar), 7.26 (d, 2H, Ar, *J* = 8.8 Hz), 2.51 (s, 3H, CH_3_), 1.34 (s, 9H, C(CH_3_)_3_). ^13^C-NMR (100 MHz, CDCl_3_) δ 176.05 (C), 170.65 (C), 167.57 (C), 156.08 (C), 146.86 (C), 146.47 (CH), 134.18 (C), 130.66 (CH), 130.43 (CH), 128.78 (CH), 122.49 (CH), 120.38 (CH), 119.45 (C), 104.59 (C), 39.28 (C), 26.95 (CH_3_), 11.65 (CH_3_). IR (ν) = 1,757 cm^−1^ (CO). ESI-MS calcd. for C_21_H_20_N_4_O_5_S, 440.47; found: m/z 441.12 [M + H]^+^. Anal. C_21_H_20_N_4_O_5_S (C, H, N).

##### 4-{[3-(Trifluoromethyl)-1H-pyrazolo[3,4-b]pyridin-1-yl]sulfonyl}phenyl pivalate (3k)

Yield = 24%; mp = 135–138°C (EtOH). ^1^H-NMR (400 MHz, CDCl_3_) δ 8.83 (d, 1H, Ar, *J* = 3.6 Hz), 8.32 (d, 2H, Ar, *J* = 8.8 Hz), 8.21 (d, 1H, Ar, *J* = 8.0 Hz), 7.46 (m, 1H, Ar), 7.27 (d, 2H, Ar, *J* = 8.8 Hz), 1.34 (s, 9H, C(CH_3_)_3_). ^13^C-NMR (100 MHz, CDCl_3_) δ 176.08 (C), 156.33 (C), 151.86 (CH), 151.10 (C), 133.81 (C), 131.90 (C), 130.42 (CH), 130.12 (CH), 122.74 (CH), 121.80 (C), 120.95 (CH), 113.95 (C), 39.27 (C), 26.92 (CH_3_). IR (ν) = 1,759 cm^−1^ (CO). ESI-MS calcd. for C_18_H_16_F_3_N_3_O_4_S, 427.40; found: m/z 428.08 [M + H]^+^. Anal. C_18_H_16_F_3_N_3_O_4_S (C, H, N).

##### 4-[(3-Cyano-1H-pyrazolo[3,4-b]pyridin-1-yl)sulfonyl]phenyl pivalate (3l)

Yield = 24%; mp = 166–169°C (EtOH). ^1^H-NMR (400 MHz, CDCl_3_) δ 8.86 (d, 1H, Ar, *J* = 4.4 Hz), 8.32 (d, 2H, Ar, *J* = 8.8 Hz), 8.24 (d, 1H, Ar, *J* = 8.0 Hz), 7.52 (m, 1H, Ar), 7.28 (d, 2H, Ar, *J* = 8.8 Hz), 1.34 (s, 9H, C(CH_3_)_3_). ^13^C-NMR (100 MHz, CDCl_3_) δ 176.06 (C), 156.62 (C), 152.34 (CH), 150.59 (C), 133.22 (C), 130.60 (CH), 129.65 (CH), 122.94 (CH), 121.50 (CH), 117.96 (C), 111.09 (C), 39.31 (C), 26.94 (CH_3_). IR (ν) = 2,250 cm^−1^ (CN), 1,762 cm^−1^ (CO). ESI-MS calcd. for C_18_H_16_N_4_O_4_S, 384.41; found: m/z 385.09 [M + H]^+^. Anal. C_18_H_16_N_4_O_4_S (C, H, N).

##### 4-{[3-(3-Methylbenzamido)-1H-indazol-1-yl]sulfonyl}phenyl pivalate (4)

Yield = 37%; oil. ^1^H-NMR (400 MHz, DMSO-d_6_) δ 11.33 (exch br s, 1H, NH), 8.16 (d, 1H, Ar, *J* = 8.4 Hz), 8.01 (d, 2H, Ar, *J* = 8.8 Hz), 7.87 (m, 3H, Ar), 7.70 (t, 1H, Ar, *J* = 7.8 Hz), 7.43 (m, 3H, Ar), 7.38 (d, 2H, Ar, *J* = 8.8 Hz), 2.39 (s, 3H, m-*CH*_3_-Ph), 1.26 (s, 9H, C(CH_3_)_3_). ^13^C-NMR (100 MHz, DMSO-d_6_) δ 176.13 (C), 166.51 (C), 155.87 (C), 148.72 (C), 141.66 (C), 138.32 (C), 133.67 (C), 133.49 (CH), 133.27 (C), 130.73 (CH), 129.58 (CH), 129.26 (CH), 128.87 (CH), 125.83 (CH), 124.77 (CH), 124.28 (CH), 123.81 (CH), 121.22 (C), 113.38 (CH), 39.36 (C), 26.99 (CH_3_), 21.36 (CH_3_). IR (ν) = 3,263 cm^−1^ (NH), 1,747 cm^−1^ (CO), 1,645 cm^−1^ (CO amide). ESI-MS calcd. for C_26_H_25_N_3_O_5_S, 491.56; found: m/z 492.15 [M + H]^+^. Anal. C_26_H_25_N_3_O_5_S (C, H, N).

#### General Procedure for Compounds (5a, 5d)

A mixture of intermediate **1b** (DeGraw and Goodman, 1964) or **1g** (Bistocchi et al., 1981) (0.93 mmol), 1.40 mmol of Na_2_CO_3_, and 4.68 mmol of CH_3_I in 4 mL of anhydrous acetonitrile was stirred at 80°C for 6 h. After cooling, the organic solvent was evaporated under vacuum, and ice-cold water was added (10–15 mL). The precipitate was filtered and washed with water to obtain compounds **5a** and **5d**, which were purified by flash column chromatography using toluene/ethyl acetate 7:3 (for **5a**) or 8:2 (for **5d**) as eluents.

##### Ethyl 1-methyl-5-nitro-1H-indole-3-carboxylate (5a)

Yield = 22%; mp = 153–155°C (EtOH). ^1^H-NMR (400 MHz, CDCl_3_) δ 9.11 (d, 1H, Ar, *J* = 2.4 Hz), 8.22 (dd, 1H, Ar, *J* = 2.4 Hz and *J* = 9.2 Hz), 7.94 (s, 1H, Ar), 7.42 (d, 1H, Ar, *J* = 9.2 Hz), 4.45 (q, 2H, *CH*_2_CH_3_, *J* = 7.2 Hz), 3.93 (s, 3H, N-CH_3_); 1.47 (t, 3H, CH_2_*CH*_3_, *J* = 7.2 Hz). ESI-MS calcd. for C_12_H_12_N_2_O_4_, 248.24; found: m/z 249.08 [M + H]^+^. Anal. C_12_H_12_N_2_O_4_ (C, H, N).

##### Ethyl 1-methyl-5-nitro-1H-indazole-3-carboxylate (5d)

Yield = 64%; mp = 178–181°C (EtOH). ^1^H-NMR (400 MHz, CDCl_3_) δ 9.18 (s, 1H, Ar), 8.36 (dd, 1H, Ar, *J* = 2.0 Hz and *J* = 9.2 Hz), 7.57 (d, 1H, Ar, *J* = 9.2 Hz), 4.59 (q, 2H, *CH*_2_CH_3_, *J* = 7.0 Hz), 4.26 (s, 3H, N-CH_3_), 1.53 (t, 3H, CH_2_*CH*_3_, *J* = 7.0 Hz). ESI-MS calcd. for C_11_H_11_N_3_O_4_, 249.23; found: m/z 250.08 [M + H]^+^. Anal. C_11_H_11_N_3_O_4_ (C, H, N).

#### General Procedure for Compounds (6a, 6b, 6d)

Compounds **5a,b,d** (**5b**: Crocetti et al., 2016) (0.60 mmol) were subjected to catalytic reduction in 96% EtOH (8 mL) for 2 h with a Parr instrument using 128 mg of 10% Pd/C as catalyst and hydrogen (30 psi). The catalyst was filtered, and the solvent was evaporated under vacuum, resulting in the desired products **6a**, **6b**, and **6d**. Compounds **6b** and **6d** were purified by crystallization from ethanol.

##### Ethyl 5-amino-1-methyl-1H-indole-3-carboxylate (6a)

Yield = 98%; oil. ^1^H-NMR (400 MHz, CDCl_3_) δ 7.67 (s, 1H, Ar), 7.50 (s, 1H, Ar), 7.13 (d, 1H, Ar, *J* = 8.2 Hz), 6.75 (d, 1H, Ar, *J* = 8.2 Hz), 4.37 (q, 2H, *CH*_2_CH_3_, *J* = 7.2 Hz), 3.75 (s, 3H, N-CH_3_), 1.41 (t, 3H, CH_2_*CH*_3_, *J* = 7.2 Hz). ESI-MS calcd. for C_12_H_14_N_2_O_2_, 218.26; found: m/z 219.11 [M + H]^+^. Anal. C_12_H_14_N_2_O_2_ (C, H, N).

##### Ethyl 5-amino-1-(3-methylbenzoyl)-1H-indole-3-carboxylate (6b)

Yield = 44%; mp = 154–157°C (EtOH). ^1^H-NMR (400 MHz, DMSO-d_6_) δ 7.97 (d, 1H, Ar, *J* = 8.8 Hz), 7.66 (s, 1H, Ar), 7.59 (s, 1H, Ar), 7.53 (m, 3H, Ar), 7.27 (d, 1H, Ar, *J* = 2.2 Hz), 6.72 (dd, 1H, Ar, *J* = 2.2 Hz and *J* = 8.8 Hz), 5.23 (exch br s, 2H, NH_2_), 4.27 (q, 2H, *CH*_2_CH_3_, *J* = 7.1 Hz), 2.42 (s, 3H, m-*CH*_3_-Ph), 1.29 (t, 3H, CH_2_*CH*_3_, *J* = 7.1 Hz). IR (ν) = 3,414–3,336 cm^−1^ (NH_2_), 1,681 cm^−1^ (CO), 1,585 cm^−1^ (CO amide). ESI-MS calcd. for C_19_H_18_N_2_O_3_, 322.36; found: m/z 323.14 [M + H]^+^. Anal. C_19_H_18_N_2_O_3_ (C, H, N).

##### Ethyl 5-amino-1-methyl-1H-indazole-3-carboxylate (6d)

Yield = 46%; mp = 151-154°C (EtOH). ^1^H-NMR (400 MHz, CDCl_3_) δ 7.43 (s, 1H, Ar), 7.29 (d, 1H, Ar, *J* = 8.8 Hz), 6.92 (dd, 1H, Ar, *J* = 2.0 Hz and *J* = 8.8 Hz), 4.51 (q, 2H, *CH*_2_CH_3_, *J* = 7.1 Hz), 4.12 (s, 3H, N-CH_3_), 1.48 (t, 3H, CH_2_*CH*_3_, *J* = 7.1 Hz). ESI-MS calcd. for C_11_H_13_N_3_O_2_, 219.24; found: m/z 220.10 [M + H]^+^. Anal. C_11_H_13_N_3_O_2_ (C, H, N).

#### General Procedure for Compounds (7a, 7b)

Compounds **7a,b** were obtained starting from intermediates **6a,b** following the same procedure described for **3d-f** and **3i-l** using 4-(chlorosulfonyl)phenyl pivalate **2** (Hwang et al., 2015) as reagent. After evaporation of the organic solvent, ice-cold water was added (20 mL). Compounds **7a,b** were recovered by extraction with CH_2_Cl_2_ (3 × 15 mL) and purified by flash column chromatography using toluene/ethyl acetate 8:2 (for **7a**) or cyclohexane/ethyl acetate 2:1 (for **7b**) as eluents.

##### Ethyl 1-methyl-5-{[4-(pivaloyloxy)phenyl]sulfonamido}-1H-indole-3-carboxylate (7a)

Yield = 64%; oil. ^1^H-NMR (400 MHz, CDCl_3_) δ 7.76 (d, 1H, Ar, *J* = 4.8 Hz), 7.75 (m, 3H, Ar), 7.18 (m, 2H, Ar), 7.11 exch br s, 1H, NH), 7.07 (d, 2H Ar, *J* = 7.2 Hz), 4.35 (q, 2H, *CH*_2_CH_3_, *J* = 7.2 Hz), 3.78 (s, 3H, N-CH_3_), 1.38 (t, 3H, CH_2_*CH*_3_, *J* = 7.2 Hz), 1.33 (s, 9H, C(CH_3_)_3_). ^13^C-NMR (100 MHz, CDCl_3_) δ 176.08 (C), 163.88 (C), 154.35 (C), 136.31 (C), 136.13 (CH), 135.45 (C), 130.70 (C), 128.91 (CH), 126.91 (C), 121.97 (CH), 119.20 (CH), 116.35 (CH), 110.51 (CH), 59.81 (CH_2_), 39.18 (C), 33.53 (CH_3_), 26.98 (CH_3_), 14.49 (CH_3_). IR (ν) = 3,242 cm^−1^ (NH), 1,747 cm^−1^ (CO), 1,714 cm^−1^ (CO). ESI-MS calcd. for C_23_H_26_N_2_O_6_S, 458.53; found: m/z 459.15 [M + H]^+^. Anal. C_23_H_26_N_2_O_6_S (C, H, N).

##### Ethyl 1-(3-methylbenzoyl)-5-{[4-(pivaloyloxy)phenyl] sulfonamido}-1H-indole-3-carboxylate (7b)

Yield = 38%; mp = 172-175°C (EtOH). ^1^H-NMR (400 MHz, CDCl_3_) δ 8.27 (d, 1H, Ar, *J* = 8.8 Hz), 8.0 (s, 1H, Ar), 7.90 (d, 1H, Ar, *J* = 2.2 Hz), 7.81 (dd, 2H, Ar, *J* = 2.2 Hz and *J* = 8.4 Hz), 7.56 (s, 1H, Ar), 7.48 (m, 3H, Ar), 7.21 (dd, 1H, Ar, *J* = 2.2 Hz and *J* = 8.8 Hz), 7.16 (dd, 2H, Ar, *J* = 2.2 Hz and *J* = 8.4 Hz), 6.64 (exch br s, 1H, NH), 4.39 (q, 2H, *CH*_2_CH_3_, *J* = 7.1 Hz), 2.47 (s, 3H, m-*CH*_3_-Ph), 1.41 (t, 3H, CH_2_*CH*_3_, *J* = 7.1 Hz), 1.35 (s, 9H, C(CH_3_)_3_). ^13^C-NMR (100 MHz, CDCl_3_) δ 176.08 (C), 163.88 (C), 154.56 (C), 139.07 (C), 136.06 (C), 134.40 (CH), 134.17 (C), 133.62 (CH), 133.35 (C), 133.0 (C), 129.89 (CH), 128.94 (CH), 128.75 (CH), 128.43 (C), 126.52 (CH), 122.17 (CH), 120.23 (CH), 117.02 (CH), 115.03 (CH), 113.33 (C), 60.75 (CH_2_), 39.22 (C), 26.99 (CH_3_), 21.36 (CH_3_), 14.37 (CH_3_). IR (ν) = 3,261 cm^−1^ (NH), 1,749 cm^−1^ (CO), 1,715 cm^−1^ (CO), 1,697 cm^−1^ (CO amide). ESI-MS calcd. for C_30_H_30_N_2_O_7_S, 562.64; found: m/z 563.18 [M + H]^+^. Anal. C_30_H_30_N_2_O_7_S (C, H, N).

#### General Procedure for Compounds (7c-e)

Compounds **7c-e** were obtained starting from intermediates **6c-e** (**6c**: Purandare et al., 2014; **6e**: Crocetti et al., 2013), respectively, following the same procedure described for **3g**. The solvent was concentrated *in vacuo* to obtain the final compounds **7c-e**, which were first purified by flash column chromatography using cyclohexane/ethyl acetate 1:1 as eluent and then by crystallization from ethanol.

##### 4-[N-(3-Cyano-1-methyl-1H-indazol-5-yl)sulfamoyl]phenyl pivalate (7c)

Yield = 63%; mp = 162–165°C (EtOH). ^1^H-NMR (400 MHz, CDCl_3_) δ 7.79 (d, 2H, Ar, *J* = 8.8 Hz), 7.49 (d, 1H, Ar, *J* = 1.2 Hz), 7.39 (m, 3H, 2H Ar and 1H NH), 7.16 (d, 2H, Ar, *J* = 8.8 Hz), 4.13 (s, 3H, N-CH_3_), 1.35 (s, 9H, C(CH_3_)_3_). ^13^C-NMR (100 MHz, CDCl_3_) δ 176.47 (C), 154.74 (C), 138.01 (C), 135.73 (C), 132.64 (C), 128.87 (CH), 125.57 (C), 124.05 (CH), 122.43 (CH), 117.15 (C), 113.32 (C), 111.89 (CH), 111.30 (CH), 39.25 (C), 36.90 (CH_3_), 26.98 (CH_3_). IR (ν) = 3,300 cm^−1^ (NH), 2,233 cm^−1^ (CN), 1,753 cm^−1^ (CO). ESI-MS calcd. for C_20_H_20_N_4_O_4_S, 412.46; found: m/z 413.12 [M + H]^+^. Anal. C_20_H_20_N_4_O_4_S (C, H, N).

##### Ethyl 1-methyl-5-{[4-(pivaloyloxy)phenyl]sulfonamido}-1H-indazole-3-carboxylate (7d)

Yield = 89%; mp = 130–133°C (EtOH). ^1^H-NMR (400 MHz, CDCl_3_) δ 7.85 (d, 2H, Ar, *J* = 6.8 Hz), 7.76 (d, 2H, Ar, *J* = 8.8 Hz), 7.30 (s, 1H, Ar), 7.08 (d, 2H, Ar, *J* = 8.8 Hz), 4.43 (q, 2H, *CH*_2_CH_3_, *J* = 7.1 Hz), 4.06 (s, 3H, N-CH_3_), 1.39 (t, 3H, CH_2_*CH*_3_, *J* = 7.1 Hz), 1.29 (s, 9H, C(CH_3_)_3_). ^13^C-NMR (100 MHz, CDCl_3_) δ 176.32 (C), 162.37 (C), 154.51 (C), 139.12 (C), 136.02 (C), 134.61 (C), 132.11 (C), 130.11 (C), 128.92 (CH), 123.75 (C), 123.40 (CH), 122.19 (CH), 115.20 (CH), 110.54 (CH), 61.10 (CH_2_), 39.18 (C), 36.50 (CH_3_), 26.96 (C), 14.38 (CH_3_). IR (ν) = 3,228 cm^−1^ (NH), 1,755 cm^−1^ (CO), 1,720 cm^−1^ (CO). ESI-MS calcd. for C_22_H_25_N_3_O_6_S, 459.52; found: m/z 460.15 [M + H]^+^. Anal. C_22_H_25_N_3_O_6_S (C, H, N).

##### Ethyl 1-(3-methylbenzoyl)-5-{[4-(pivaloyloxy)phenyl]sulfonamido}-1H-indazole-3-carboxylate (7e)

Yield = 64%; mp = 180–182°C (EtOH). ^1^H-NMR (400 MHz, CDCl_3_) δ 8.46 (d, 1H, Ar, *J* = 8.8 Hz), 7.98 (s, 1H, Ar), 7.92 (s, 2H, Ar), 7.82 (d, 2H, Ar, *J* = 8.8 Hz), 7.43 (m, 3H, Ar), 7.18 (d, 2H, Ar, *J* = 8.8 Hz), 6.68 (exch br s, 1H, NH), 4.53 (q, 2H, *CH*_2_CH_3_, *J* = 7.1 Hz), 2.47 (s, 3H, m-*CH*_3_-Ph), 1.48 (t, 3H, CH_2_*CH*_3_, *J* = 7.1 Hz), 1.34 (s, 9H, C(CH_3_)_3_). ^13^C-NMR (100 MHz, CDCl_3_) δ 168.15 (C), 161.51 (C), 154.79 (C), 140.66 (C), 139.26 (C), 138.04 (C), 135.83 (C), 134.30 (C), 133.92 (CH), 132.05 (CH), 131.70 (C), 128.92 (CH), 128.08 (CH), 125.09 (C), 124.62 (CH), 122.38 (CH), 116.76 (CH), 114.44 (CH), 61.93 (CH_2_), 39.23 (C), 26.99 (CH_3_), 21.36 (CH_3_), 14.25 (CH_3_). IR (ν) = 3,250 cm^−1^ (NH), 1,745 cm^−1^ (CO), 1,712 cm^−1^ (CO), 1,698 cm^−1^ (CO amide). ESI-MS calcd. for C_29_H_29_N_3_O_7_S, 563.63; found: m/z 564.18 [M + H]^+^. Anal. C_29_H_29_N_3_O_7_S (C, H, N).

#### General Procedure for Compounds (9a, 9b)

Compounds **9a,b** were obtained starting from intermediates **8a,b** (**8a**: Muthupplaniappan et al., 2009; **8b**: Giovannoni et al., 2016) following the same procedure described for **3d-f, 3i-l**, and **7a,b**. Compounds **9a,b** were recovered by extraction with CH_2_Cl_2_ (3 × 15 mL) and purified by flash column chromatography using cyclohexane/ethyl acetate 2:1 (for **9a**) or petroleum ether/ethyl acetate 5:1 (for **9b**) as eluents.

##### 4-[(4-Oxocinnolin-1(4H)-yl)sulfonyl]phenyl pivalate (9a)

Yield = 8%; mp = 116–119°C (EtOH). ^1^H-NMR (400 MHz, CDCl_3_) δ 8.63 (d, 1H, Ar, *J* = 8.6 Hz), 8.27 (d, 1H, Ar, *J* = 8.6 Hz), 8.08 (d, 2H, Ar, *J* = 8.8 Hz), 7.83 (s, 1H, Ar), 7.81 (t, 1H, Ar, *J* = 7.6 Hz), 7.51 (t, 1H, Ar, *J* = 7.6 Hz), 7.30 (d, 2H, Ar, *J* = 8.8 Hz), 1.36 (s, 9H, C(CH_3_)_3_). ^13^C-NMR (100 MHz, CDCl_3_) δ 175.99 (C), 170.78 (C), 156.26 (C), 141.52 (CH), 139.39 (C), 134.93 (CH), 133.27 (C), 130.16 (CH), 126.70 (CH), 123.79 (C), 122.82 (CH), 117.05 (CH), 39.33 (C), 26.96 (CH_3_). IR (ν) = 1,745 cm^−1^ (CO), 1,716 cm^−1^ (CO). ESI-MS calcd. for C_19_H_18_N_2_O_5_S, 386.42; found: m/z 387.10 [M + H]^+^. Anal. C_19_H_18_N_2_O_5_S (C, H, N).

##### 4-[(3-Cyclopropyl-4-oxocinnolin-1(4H)-yl)sulfonyl]phenyl pivalate (9b)

Yield = 25%; mp = 121–123°C (EtOH). ^1^H-NMR (400 MHz, CDCl_3_) δ 8.62 (d, 1H, Ar, *J* = 8.8 Hz), 8.29 (d, 1H, Ar, *J* = 8.8 Hz), 8.02 (d, 2H, Ar, *J* = 8.8 Hz), 7.74 (t, 1H, Ar, *J* = 7.6 Hz), 7.46 (t, 1H, Ar, *J* = 7.6 Hz), 7.28 (d, 2H, Ar, *J* = 8.8 Hz), 2.54 (m, 1H, CH C_3_H_5_), 1.36 (s, 9H, C(CH_3_)_3_), 0.99 (m, 2H, CH_2_ C_3_H_5_), 0.85 (m, 2H, CH_2_ C_3_H_5_). ^13^C-NMR (100 MHz, CDCl_3_) δ 176.0 (C), 170.55 (C), 156.04 (C), 153.20 (C), 139.71 (C), 134.36 (CH), 133.70 (C), 130.15 (CH), 126.42 (CH), 125.93 (CH), 122.53 (CH), 122.0 (C), 116.86 (CH), 39.31 (C), 26.97 (CH_3_), 9.90 (CH_2_), 9.09 (CH). IR (ν) = 1,745 cm^−1^ (CO), 1,732 cm^−1^ (CO). ESI-MS calcd. for C_22_H_22_N_2_O_5_S, 426.49; found: m/z 427.13 [M + H]^+^. Anal. C_22_H_22_N_2_O_5_S (C, H, N).

#### Procedure for 4-[(5-Oxo-4-phenylisoxazol-2(5H)-yl)sulfonyl]phenyl Pivalate (11)

Compound **11** was obtained starting from intermediate **10** (Becalli et al., 1984) by reaction with 4-(chlorosulfonyl)phenyl pivalate **2** (Hwang et al., 2015) following the general procedure described for **3g** and **7c-e**. The pyridine was concentrated *in vacuo* to obtain the final compound **11**, which was purified by flash column chromatography using hexane/acetone 1:1 as eluent. Yield = 18%; mp = 166–168°C (EtOH). ^1^H-NMR (400 MHz, CDCl_3_) δ 8.35 (s, 1H, CH), 7.93 (d, 2H, Ar, *J* = 8.8 Hz), 7.66 (d, 2H, Ar, *J* = 8.0 Hz), 7.37 (d, 3H, Ar, *J* = 6.4 Hz), 7.31 (d, 2H, Ar, *J* = 8.8 Hz), 1.34 (s, 9H, C(CH_3_)_3_). ^13^C-NMR (100 MHz, CDCl_3_) δ 165.85 (C), 156.91 (C), 143.93 (CH), 131.16 (CH), 129.56 (CH), 128.99 (CH), 128.13 (C), 126.37 (C), 126.24 (CH), 122.89 (CH), 115.15 (C), 39.36 (C), 26.96 (CH_3_). IR (ν) = 3,099 cm^−1^ (CH isox), 1,759 cm^−1^ (CO), 1,745 cm^−1^ (CO), 1,597 cm^−1^ (C_3_=C_4_). ESI-MS calcd. for C_20_H_19_NO_6_S, 401.43; found: *m/z* 402.10 [M+H]^+^. Anal. C_20_H_19_NO_6_S (C, H, N).

### Pharmacology

Compounds were dissolved in 100% DMSO at 5 mM stock concentrations. The final concentration of DMSO in the reactions was 1%, and this level of DMSO had no effect on enzyme activity. HNE inhibition assays were performed in black flat-bottom 96-well microtiter plates. Briefly, a solution containing 200 mM Tris–HCl, pH 7.5, 0.01% bovine serum albumin, 0.05% Tween®-20, and 20 mU/mL of HNE (Calbiochem) was added to wells containing different concentrations of each compound. The reaction was initiated by addition of 25 μM elastase substrate (N-methylsuccinyl-Ala-Ala-Pro-Val-7-amino-4-methylcoumarin, Calbiochem) in a final reaction volume of 100 μL/well. Kinetic measurements were obtained every 30 s for 10 min at 25°C using a Fluoroskan Ascent FL fluorescence microplate reader (Thermo Electron, MA) with excitation and emission wavelengths of 355 and 460 nm, respectively. For all compounds tested, the concentration of inhibitor that caused 50% inhibition of the enzymatic reaction (IC_50_) was calculated by plotting % inhibition vs. logarithm of inhibitor concentration (at least six points). The data are presented as the mean values of at least three independent experiments with relative standard deviations of <15%.

### Analysis of Compound Stability

Spontaneous hydrolysis of selected derivatives was evaluated at 25°C in 0.05 M phosphate buffer, pH 7.3. Kinetics of compound hydrolysis was monitored by measuring changes in absorbance spectra over time using a SpectraMax ABS Plus microplate spectrophotometer (Molecular Devices, Sunnyvale, CA). Absorbance (A_t_) at the characteristic absorption maxima of each compound was monitored over time until no further absorbance decreases occurred (A_∞_). Using these measurements, we created semilogarithmic plots of log(A_t_-A_∞_) *vs*. time, and k′ values were determined from the slopes of these plots. Half-conversion times were calculated using t_1/2_ = 0.693/k′.

### Molecular Modeling

Structures of Sivelestat (in the form of a carboxylate anion) and compounds **3a**, **7b**, **7d**, and **7e**, were created using ChemOffice 2016 software, pre-optimized with the MM2 force field and saved in Tripos MOL2 format. The ligand structures were then imported into the Molegro Virtual Docker 6.0 program (MVD). The structure of HNE complexed with 1-{3-methyl-2-[4-(morpholine-4-carbonyl)-benzoylamino]-butyryl}-pyrrolidine-2-carboxylic acid (3,3,4,4,4-pentafluoro-1-isopropyl-2-oxo-butyl)-amide (SEI) ligand was downloaded from the Protein Data Bank (PDB code 1B0F) and also imported into MVD. The co-crystallized water molecules were removed from the 1B0F structure on importing. A search space for docking was defined in the HNE binding site as a sphere of radius 12 Å positioned at the geometric center of gravity of the SEI ligand, and the investigated compounds were docked into the binding site. MolDock score functions (Thomsen and Christensen, 2006) were applied with a 0.3 Å grid resolution. Ligand flexibility was accounted for with respect to torsion angles auto-detected in MVD. Structure of the protein was considered rigid. The “Internal HBond” and “sp2-sp2 torsions” options were activated in the “Ligand evaluation” menu of the MVD Docking Wizard. Three hundred docking runs were performed for each molecule. Our attempts to enhance number of docking runs up to 600 did not lead to better scored docking poses. The option “Return multiple poses for each run” was enabled, and the post-processing options “Energy minimization” and “Optimize H-bonds” were applied after docking. Similar poses were clustered at a RMSD threshold of 1 Å.

## Results and Discussion

### Chemistry

**Scheme 1:**
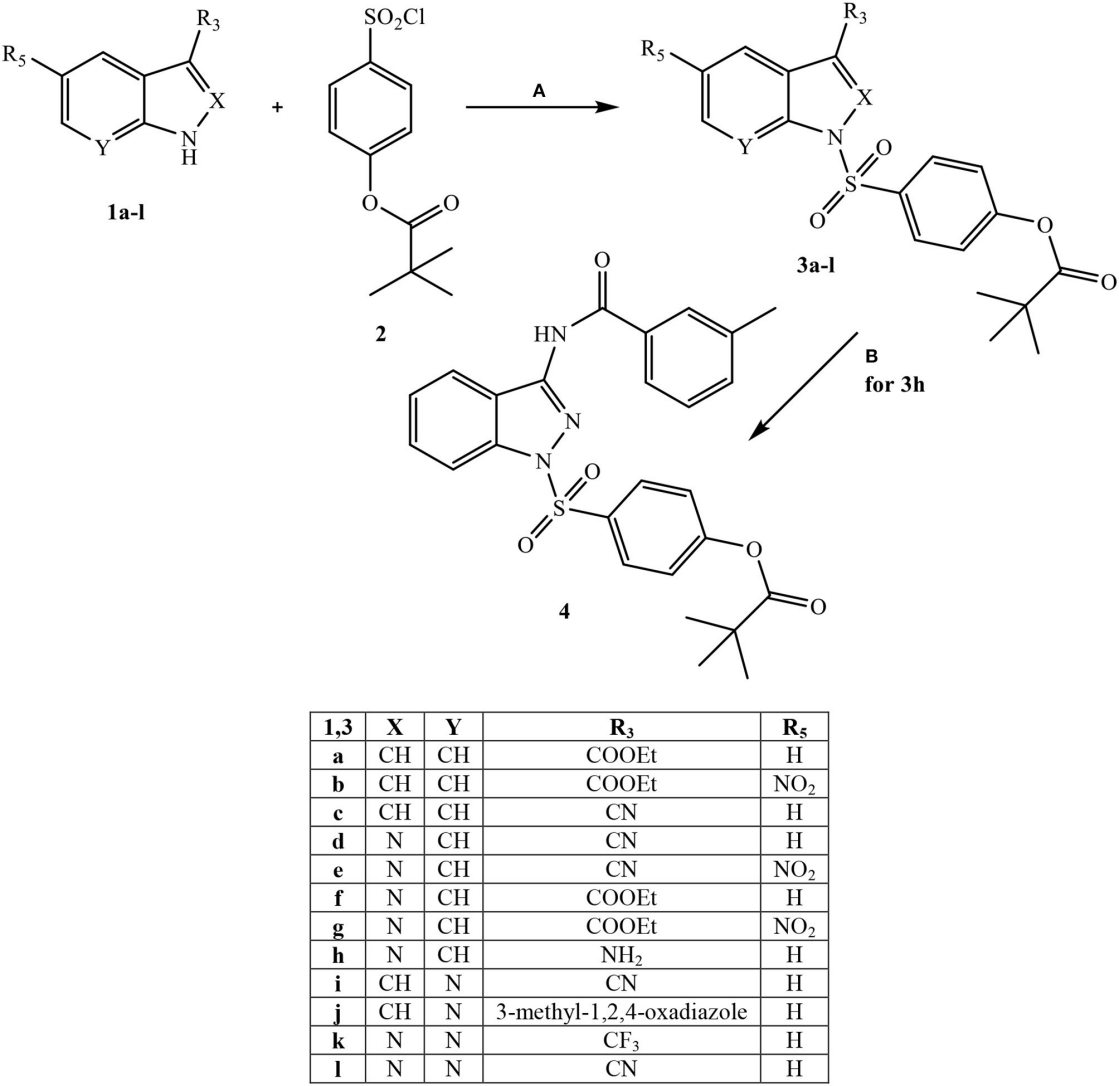
^a^Reagents and conditions. **(A)** for **3a-c:** NaH (60% dispersion in mineral oil), anhydrous THF, r.t., 24 h; for **3d-f** and **3i-l:** Et_3_N, anhydrous CH_2_Cl_2_, 0°C, 2 h then r.t., 2 h; for **3g:** dry pyridine, r.t., 4 h; for **3h:** Et_3_N, dry DMF, 1,4-dioxane, 50 °C, 4h; **(B)** m-Toluoyl chloride, Et_3_N, anhydrous CH_2_Cl_2_, 0°C, 2 h then r.t., 2 h.

**Scheme 2:**
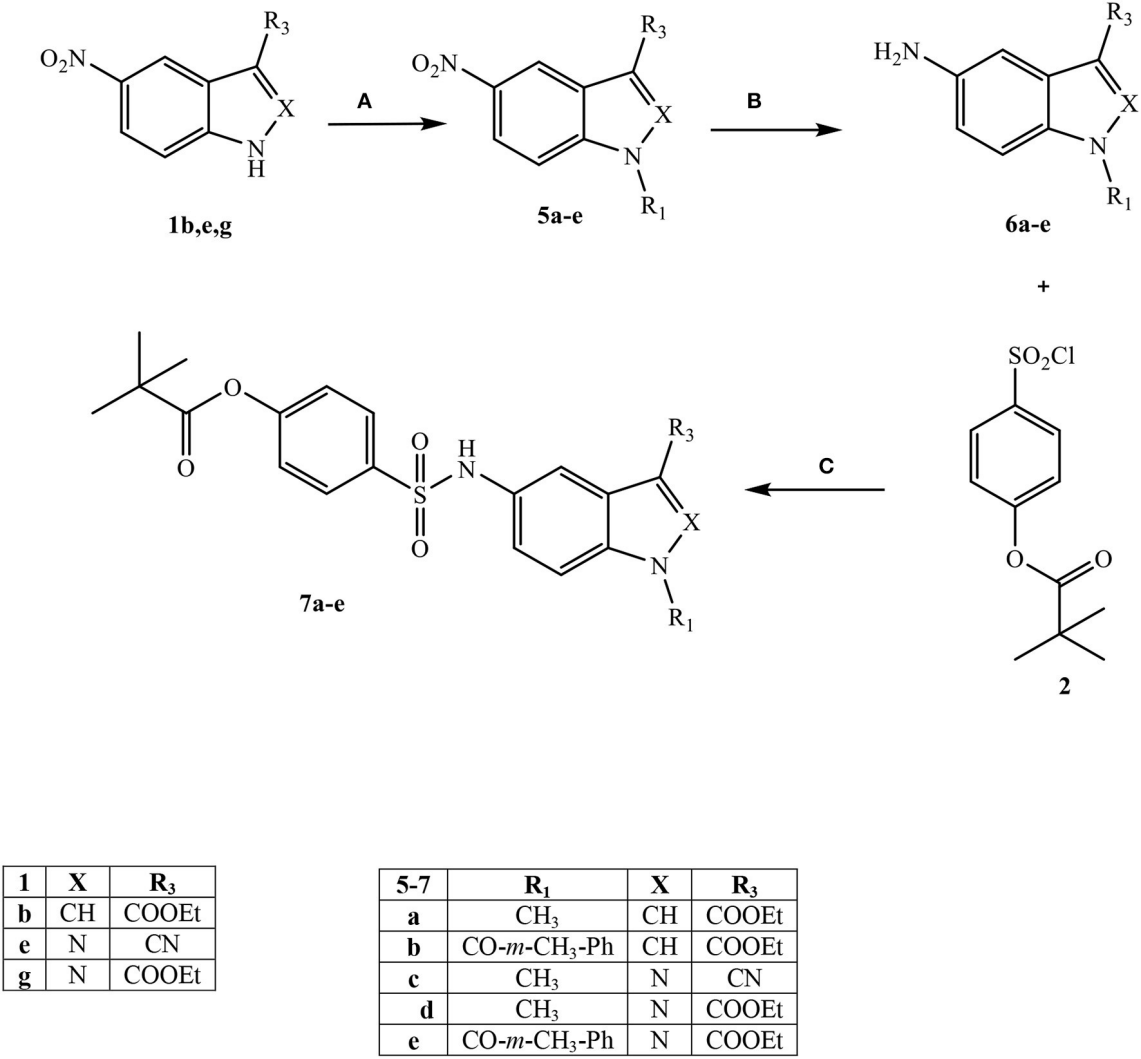
^a^Reagents and conditions. **(A)** for **5a,c,d:** CH_3_I, Na_2_CO_3_, anhydrous CH_3_CN, 80 C, 6 h; for **5b:** *m*-Toluoyl chloride, NaH (60% dispersion in mineral oil), anhydrous THF, r.t., 24 h; for **5e:** *m*-Toluoyl chloride, Et_3_N, anhydrous CH_2_Cl_2_, 0°C, 2 h then r.t., 2 h **(B)** H_2_ (Parr), Pd/C, EtOH 96%, 30 min (for **6c**) and 2 h (for **6a,b,d,e**); **(C) for 7a,b:** Et_3_N, anhydrous CH_2_Cl_2_, 0°C, 2 h then r.t., 2 h; for **7c-e:** dry pyridine, r.t., 4 h.

**Scheme 3:**
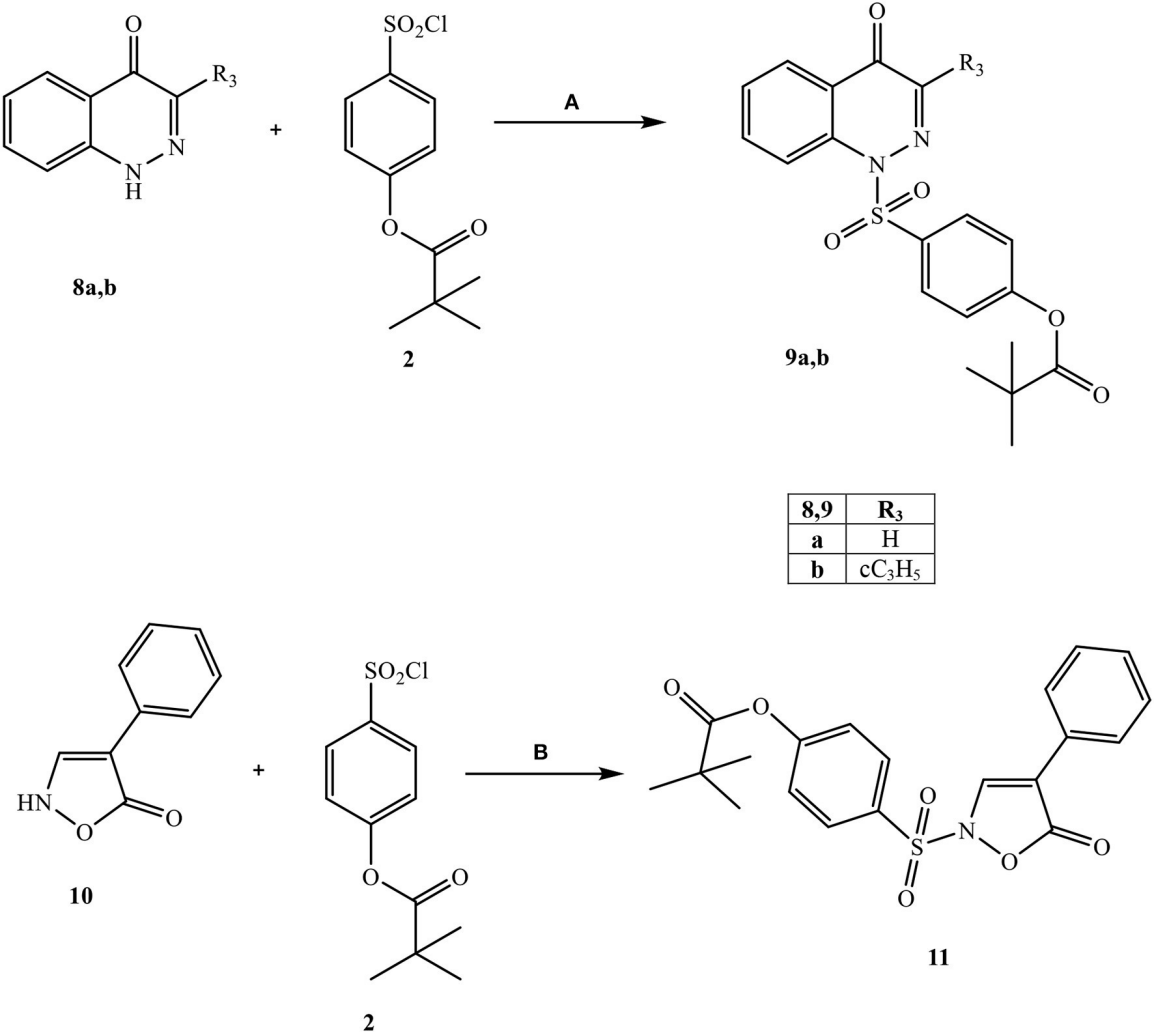
^a^Reagents and conditions. **(A)** Et_3_N, anhydrous CH_2_Cl_2_, 0°C, 2 h then r.t., 2 h; **(B)** dry pyridine, r.t., 4 h.

All final compounds were synthesized as reported in Schemes 1–3, and the structures were confirmed on the basis of analytical and spectral data. The 4-(chlorosulfonyl)phenyl pivalate fragment **2** representing the active portion of Sivelestat that was incorporated into all new compounds was synthesized as reported previously (Hwang et al., 2015).

Starting from Scheme 1, fragment **2** was inserted at the N-1 position of four different bi-heterocycles that were previously investigated by our research group as N(1)-CO- aryl(alkyl) derivatives: indoles (Crocetti et al., 2016), indazoles (Crocetti et al., 2011, 2013), 7-azaindoles (Crocetti et al., 2018; Giovannoni et al., 2019) and 7-azaindazoles (data not shown). Indole derivatives **3a-c** were obtained starting from the precursors **1a-c** (**1a**: Shahidul et al., 2006; **1b**: DeGraw and Goodman, 1964; **1c**: Yuen et al., 2013) by treatment with 4-(chlorosulfonyl)phenyl pivalate **2** and sodium hydride in anhydrous tetrahydrofuran (THF) at room temperature. To obtain the final indazoles **3d-g** two different procedures were followed: treatment of the appropriate intermediate **1d-g** (**1d:** Alaime et al., 2018; **1e,g:** Crocetti et al., 2013; **1f:** Crocetti et al., 2011) with the sulfonyl chloride **2** in anhydrous CH_2_Cl_2_ and Et_3_N (compounds **3d-f**) or in dry pyridine at room temperature (**3g**). The 3-aminoindazole **1h** (Lefebvre et al., 2010) was reacted with 4-(chlorosulfonyl)phenyl pivalate **2** in dry 1,4-dioxane/DMF, Et_3_N at 50 °C to obtain the intermediate **3h**, which was further elaborated by treatment with m-toluoyl chloride in anhydrous CH_2_Cl_2_ and Et_3_N to obtain the final compound **4**, containing a benzamido moiety at position 3. Lastly, synthesis of the 7-azaindoles **3i**, **j** and 7-azaindazoles **3k**, **l** was performed starting from precursors **1i,j** and **1-k,l** (**1i**: Bahekar et al., 2007; **1j**: Crocetti et al., 2018; **1k,l**: Schirok et al., 2015), respectively, under the same conditions described for compounds **3d-f**.

Scheme 2 shows the synthetic route followed to obtain compounds **7a-e** bearing the active fragment of Sivelestat at position 5 of the indazole or indole scaffolds. Treatment of precursors **1b**, **1e**, and **1g** with iodomethane, sodium carbonate in anhydrous acetonitrile at reflux afforded intermediates **5a**, **c**, **d** (**5c**: Purandare et al., 2014), while compounds **5b** and **5e** were obtained by treatment of **1b** with m-toluoyl chloride in anhydrous dichloromethane and Et_3_N and **1g** with m-toluoyl chloride and sodium hydride in tetrahydrofuran, respectively (**5b**: Crocetti et al., 2016; **5e**: Crocetti et al., 2013). The 5-NO_2_ derivatives **5a-e** were transformed into the corresponding 5-amino compounds **6a-e** (**6b**: Crocetti et al., 2016; **6c**: Purandare et al., 2014) through catalytic reduction with a Parr instrument and a subsequent reaction with 4-(chlorosulfonyl)phenyl pivalate **2** (Hwang et al., 2015) under the same conditions reported in Scheme 1, leading to compounds **7a-e**. Finally, Scheme 3 shows the synthesis of the cinnolinone derivatives of type **9** and isoxazolone **11**. In all precursors **8a**, **b** (**8a**: Muthupplaniappan et al., 2009; **8b**: Giovannoni et al., 2016) and **10** (Becalli et al., 1984), the insertion of fragment **2** was carried out under the same conditions as described in Scheme 1.

### Biological Evaluation

All new products were evaluated for HNE inhibitory activity, and the results are reported in Tables 1, **2** in comparison with Sivelestat. Table 1 presents the results of compounds lacking the 1-N-CO function responsible for activity in our original compounds (Figure 3). These include **3a-g**, **3i-l**, **4**, and **9a**, **b**, which contain the sulfamoyl fragment of Sivelestat at N-1 of the bicyclic nucleus, and compounds **7a**, **7c**, and **7d**, which have the (sulfonyl)phenyl pivalate chain at position five of the nucleus and a methyl group at N-1 (N-methyl derivatives). Many of the new derivatives exhibited very potent HNE inhibitory activity, with IC_50_ values between 15 and 78 nM for most compounds, which is comparable to or better that that of Sivelestat (IC_50_ = 44 nM). The most potent compounds were the indazole **3f** and the cinnoline **9a**, which had IC_50_ values of 15 and 19 nM, respectively. These results clearly demonstrated that replacement of the NCO function at N-1 with the active fragment of Sivelestat did not affect HNE inhibitory activity (Table 1). Previously, we found that indoles of type B (Figure 3), which were designed as 2-deaza analogs of highly active N-benzoylindazole compounds, were inactive or low activity HNE inhibitors due to the lack of the nitrogen at position two, which forms an important interaction with Gly193 of the catalytic site (Crocetti et al., 2016). Here, we report that introduction of the (sulfonyl)phenyl pivalate chain at N-1 of the indole nucleus into these compounds (i.e., **3a-c**) resulted in very potent HNE inhibitors, with IC_50_ values of 30, 25, and 49 nM, respectively, suggesting that inclusion of the active fragment of Sivelestat leads to a different interaction of the indole scaffold with HNE. Finally, the N-1 methyl derivatives **7a**, **c**, **d** bearing the (sulfonyl)phenyl pivalate chain at position five of the nucleus retained some inhibitory activity, although they were not as active as the other compounds described above.

**Table 1:** HNE inhibitory activity of compounds **3a-g, 3i-l**, **4, 7a,c,d**, and **9a,b**.

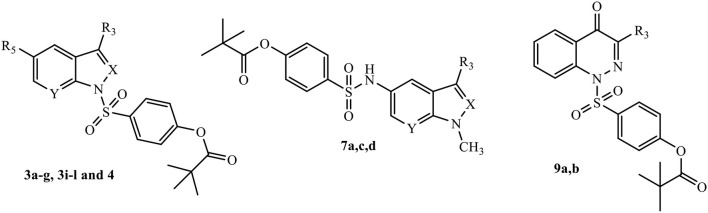					
**Compound**	**X**	**Y**	**R**_**3**_	**R**_**5**_	**IC**_**50**_ **(nM)**^**a**^
**3a**	CH	CH	COOEt	H	30 ± 9
**3b**	CH	CH	COOEt	NO_2_	25 ± 8
**3c**	CH	CH	CN	H	49 ± 13
**3d**	N	CH	CN	H	76 ± 11
**3e**	N	CH	CN	NO_2_	93 ± 27
**3f**	N	CH	COOEt	H	15 ± 4
**3g**	N	CH	COOEt	NO_2_	23 ± 10
**3i**	CH	N	CN	H	55 ± 12
**3j**	CH	N	3-methyl-1,2,4-oxadiazole	H	43 ± 14
**3k**	N	N	CF_3_	H	135 ± 37
**3l**	N	N	CN	H	66 ± 9
**4**	N	CH	NH-CO-m-CH_3_-Ph	H	62 ± 12
**7a**	CH	CH	COOEt	-	233 ± 76
**7c**	N	CH	CN	-	394 ± 38
**7d**	N	CH	COOEt	-	86 ± 13
**9a**	-	-	H	-	19 ± 5
**9b**	-	-	cC_3_H_5_	-	78 ± 18
**Sivelestat**					44 ± 20

a *IC_50_ values are presented as the mean ± SD of three independent experiments*.

The results reported in the Table 1 indicate that in practice, the pivaloyl fragment of Sivelestat can “replace” the role of the N-CO group at position 1 and offer a different point of attack for Ser195. It is also clear that the selected scaffolds with adequate substitutions are appropriate carriers for the Sivelestat pharmacophore. On the other hand, compounds maintaining the NCO function at position 1 (compounds **7b** and **7e**) or CO at position five (compound **11**) (Table 2) as the point of attack for Ser195, and simultaneously bearing the active pivaloyl fragment of Sivelestat, only exhibited moderate HNE inhibitory activity (IC_50_ = 0.29–5.2 μM), with the exception of the previously published compound G (IC_50_ = 59 nM), which has activity comparable to its analogs lacking the Sivelestat fragment (Giovannoni et al., 2018). However, these data also clearly indicate that this strategy does not produce the expected additive effect, probably due to the increased hindrance of the molecules.

**Table 2:** HNE inhibitory activity of compounds 7b, e, 11 in comparison with compound G and Sivelestat.

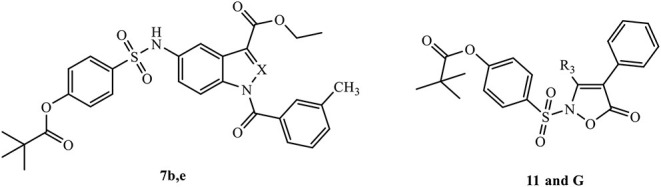			
**Compound**	**X**	**R**_**3**_	**IC**_**50**_ **(μM)**^**a**^
**7b**	CH	-	5.2 ± 0.14
**7e**	N	-	0.460 ± 0.12
**11**	-	H	0.290 ± 0.04
**G**^***b***^	-	CH_3_	0.059 ± 0.02
**Sivelestat**			0.044 ± 0.02

a IC_50_ values are presented as the mean ± SD of three independent experiments.

b *(Giovannoni et al., 2018)*.

A set of the most potent HNE inhibitors, as well as low activity compound **7b**, were evaluated for their chemical stability in aqueous buffer. Spontaneous hydrolysis rates of the inhibitors were measured in phosphate buffer at pH 7.3 and 25°C. As shown in Table 3, compounds **3a**, **3c**, **3f**, and **7d** had a relatively good stability (t_1/2_ > 9 h) with high HNE inhibitory activity (IC_50_ <100 nM).

**Table 3:** Half-life (t_1/2_) for the spontaneous hydrolysis of selected derivatives.

**Compound**	**t_**1/2**_ (h)**	**Absorption wavelength** **(nm)^**a**^**
**3a**	9.6	280
**3b**	6.1	275
**3c**	14.4	270
**3d**	6.1	315
**3e**	0.4	260
**3f**	10.5	265
**3g**	1.8	270
**3i**	1.8	0.270
**3j**	2.5	290
**3k**	1.3	290
**3l**	3.5	295
**4**	6.4	260
**7a**	6.8	295
**7b**	5.3	280
**7c**	1.5	270
**7d**	16.5	280
**7e**	5.8	285
**9a**	2.1	260
**9b**	1.9	270
**G**	5.8	290

a *Absorption used for monitoring spontaneous hydrolysis*.

**Figure 4:**
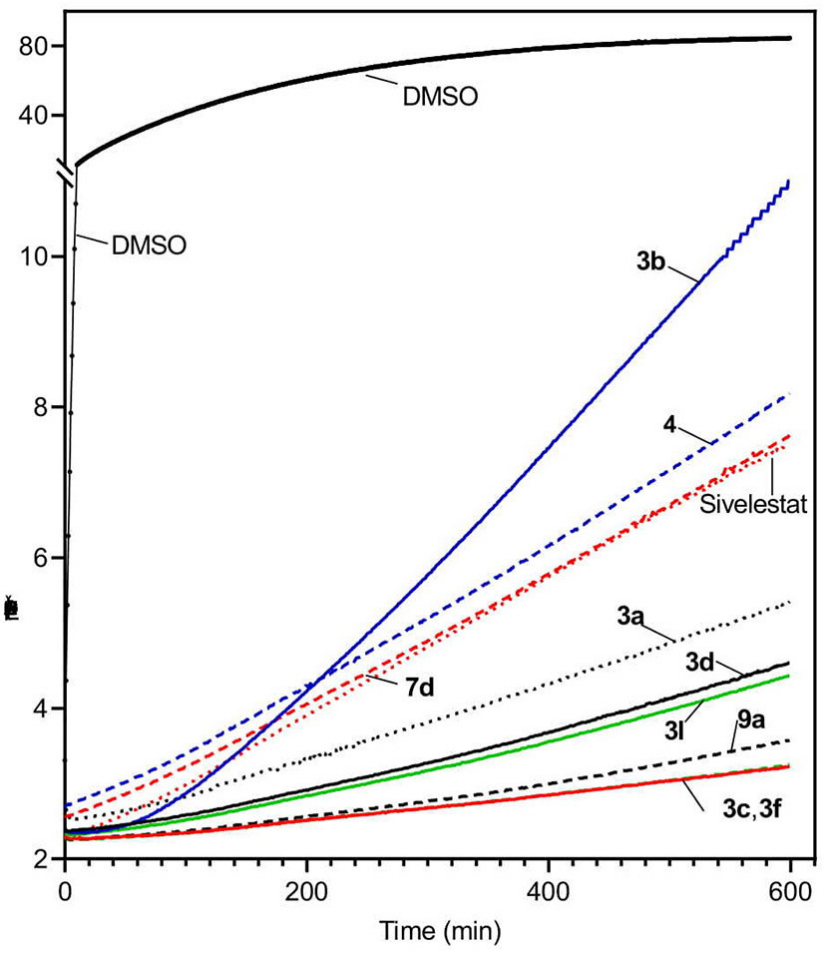
Evaluation of HNE inhibition by representative sulfonamides and Sivelestat over time. HNE was incubated with the indicated selected compounds (5 μM), and kinetic curves monitoring substrate cleavage catalyzed by HNE over time are shown. Representative curves are from two independent experiments.

The relatively high enzymatic stability of HNE allowed us to evaluate reversibility of the enzyme inhibition over time. As an example, Figure 4 shows kinetic curves monitoring substrate cleavage catalyzed by HNE over a 10-h period in the presence of selected sulfonamide derivatives (5 μM) and compared to Sivelestat. Persistence of selected HNE inhibitors over an extended period of time (16 h) was also evaluated and showed that the most effective HNE inhibitors over time were **3c**, **3d**, **3i**, **3l**, and **9a** (Table 4). Inhibitory activities of these compounds were comparable or better than Sivelestat in this assay.

**Table 4:** Potency of selected HNE inhibitors (with IC_50_ <100 nM) over extended period of time (16 h).

**Compounds**	**Remaining HNE activity (%)^**a**^**
**3a**	5.5 ± 1.8
**3b**	20.5 ± 1.7
**3c**	2.0 ± 0.4
**3d**	4.3 ± 0.3
**3e**	41.7 ± 3.2
**3f**	5.9 ± 1.2
**3g**	20.1 ± 1.6
**3i**	2.6 ± 0.5
**3j**	26.2 ± 3.4
**3l**	4.5 ± 1.7
**4**	8.6 ± 1.7
**7d**	9.1 ± 2.5
**9a**	2.2 ± 0.1
**9b**	9.2 ± 2.5
**Sivelestat**	3.7 ± 0.1

a *Enzymatic activity of HNE was monitored during 16 h in the presence of 10 μM inhibitor*.

### Molecular Modeling

Molecular docking studies of some HNE inhibitors, including Sivelestat and triterpenes, were previously made (Feng et al., 2012, 2013) based on 1B0F structure from PDB. Hence, we also used this structure in our docking calculations. The MVD program was validated on HNE by confirming the ability of the program to reproduce the position of the co-crystallized SEI ligand contained in the 1B0F structure taken from the Protein Data Bank (Cregge et al., 1998). Independent docking of SEI into the HNE binding site was performed, and comparison of the resulting pose with the experimental ligand location demonstrated that the MVD program accurately reproduced the location of the SEI ligand in the HNE binding site (RMSD of non-hydrogen atom positions between the two structures is 1.24 Å) (Figure S1, see Supplementary Material).

**Figure 5:**
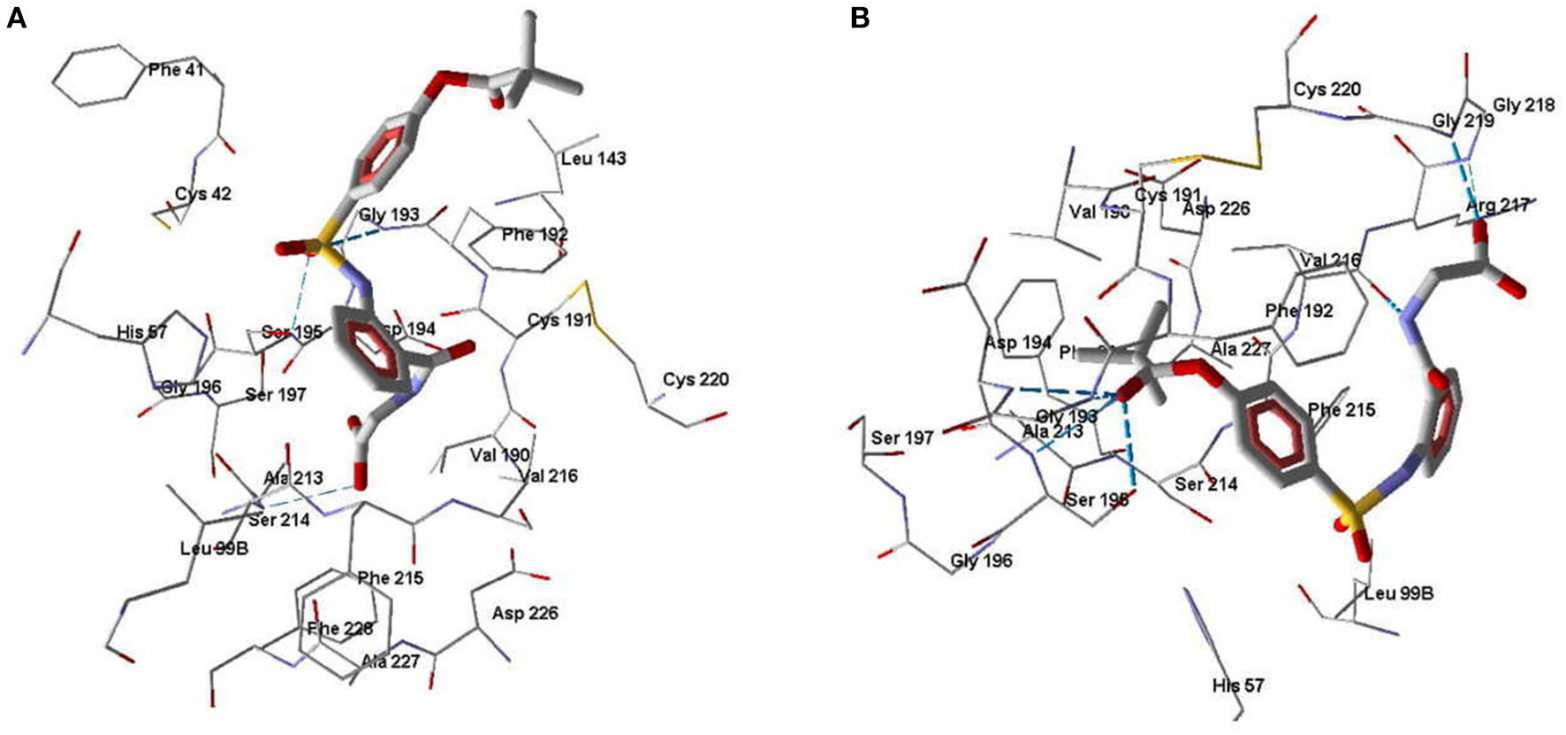
Docking poses of Sivelestat. **(A)** The lowest-score docking pose of Sivelestat. **(B)** Alternative docking pose of Sivelestat. Residues within 5 Å from the pose are visible. H-bonds are shown by blue dashed lines.

One of the goals of our molecular modeling study consisted in clarifying the possibilities for Nakayama's mechanism for inhibitory action of pivaloyl-containing compounds (Nakayama et al., 2002) via an attack of Ser195 hydroxyl oxygen atom at the carbon center of C=O group in the inhibitor molecule. Docking of Sivelestat in its anionic form into HNE using MVD software gave a ligand position (Figure 5A) similar to the pose obtained by Feng and co-authors with the free docking program AutoDock (Feng et al., 2012). In this docking pose, Sivelestat forms H-bonds between the oxygen atom of the sulfonamide group and Ser195 and Gly193, as well as an H-bond between the carboxyl group and Ser214. In addition, the Sivelestat pose is near to hydrophobic residues Leu99B, Phe192, His57, Val216, Cys191, and Phe41. All of these features of the ligand location are consistent with the docking results previously reported for Sivelestat (Feng et al., 2012). According to our data and the results obtained by Feng et al. (2012) on the binding of Sivelestat to HNE, Ser195 forms an H-bond with the sulfonamide oxygen atom, hence Ser195 is far from the carbonyl carbon atom of the pivaloyl group, which is a potential reaction center in the reported mechanism (Nakayama et al., 2002). Thus, for the docking pose of Sivelestat, we calculated the distance O(Ser195)····C=O(pivaloyl) to be 9.4 Å. In this regard, the experimental results of Nakayama and co-authors (Nakayama et al., 2002) can be explained by the presence of other possibilities for binding of Sivelestat to HNE using conformations other than the optimal docking pose that we obtained. Indeed, we found another pose for Sivelestat in which the pivaloyl group is located close to the elastase catalytic triad, forming H-bonds with Ser195 and Asp194. In addition, H-bonds were formed between the amide nitrogen atom of the ligand and Val216 and between the carboxyl group and Gly218 and Gly219 (Figure 5B). This alternative docking pose of Sivelestat is favorable for nucleophilic attack of the Ser195 oxygen atom on the carbonyl carbon of the pivaloyl group, resulting the distance O(Ser195)····C=O(pivaloyl) of 3.04 Å, which is consistent with the reported mechanism (Nakayama et al., 2002). It should be noted that the MolDock score for this pose is only 1.4 units higher than that for the optimal pose shown in Figure 5A.

**Figure 6:**
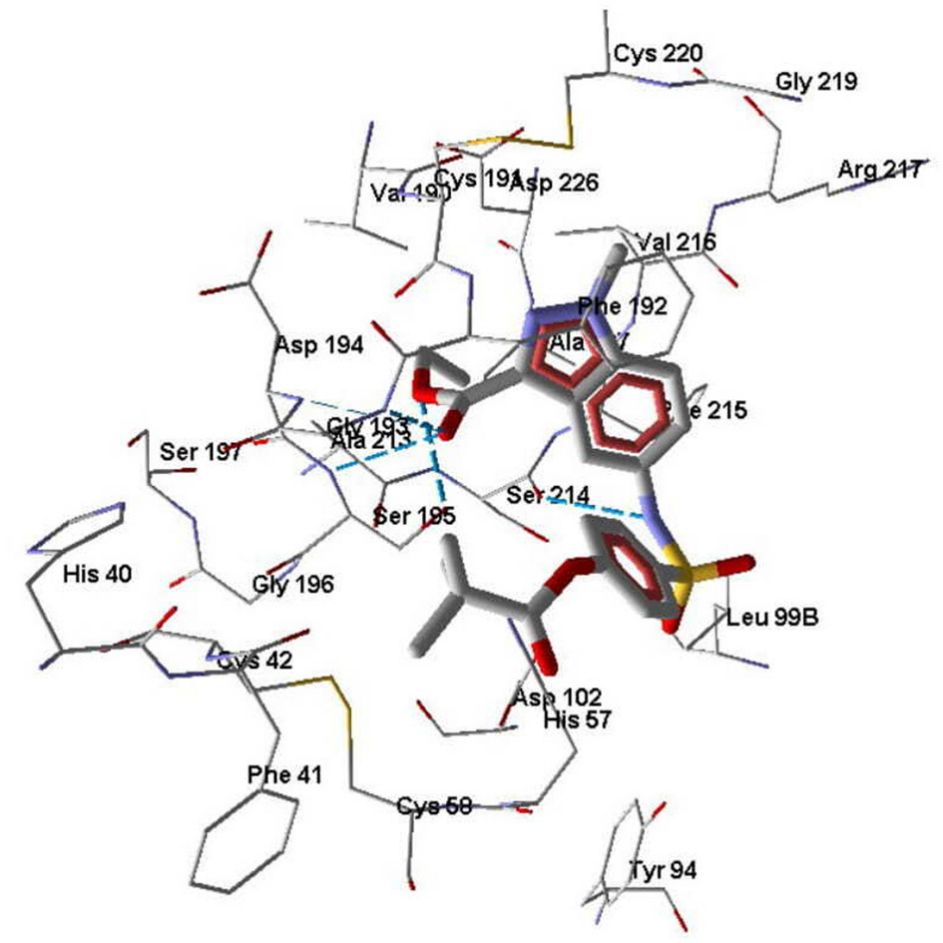
Docking pose of compound **7d**. Residues within 5 Å from the pose are visible. H-bonds are shown by blue dashed lines.

Molecule **7d** forms H-bonds with Ser195 via participation of both oxygen atoms of the ethoxycarbonyl group. In addition, the carbonyl oxygen of the ethoxycarbonyl substituent forms Hbonds with Gly193 and Asp194, while the sulfonamide nitrogen atom is H-bonded to Ser214 (Figure 6). The docking pose of molecule **7d** in the HNE binding site is characterized by a distance of 5.40 Å between the oxygen atom of Ser195 and the carbonyl carbon of the pivaloyl group. This does not exclude the possibility of nucleophilic addition of Ser195 to the C=O group according to the reported mechanism (Nakayama et al., 2002), because as a result of thermal movements of the ligand and receptor, the carbonyl oxygen may be available for nucleophilic attack.

**Figure 7:**
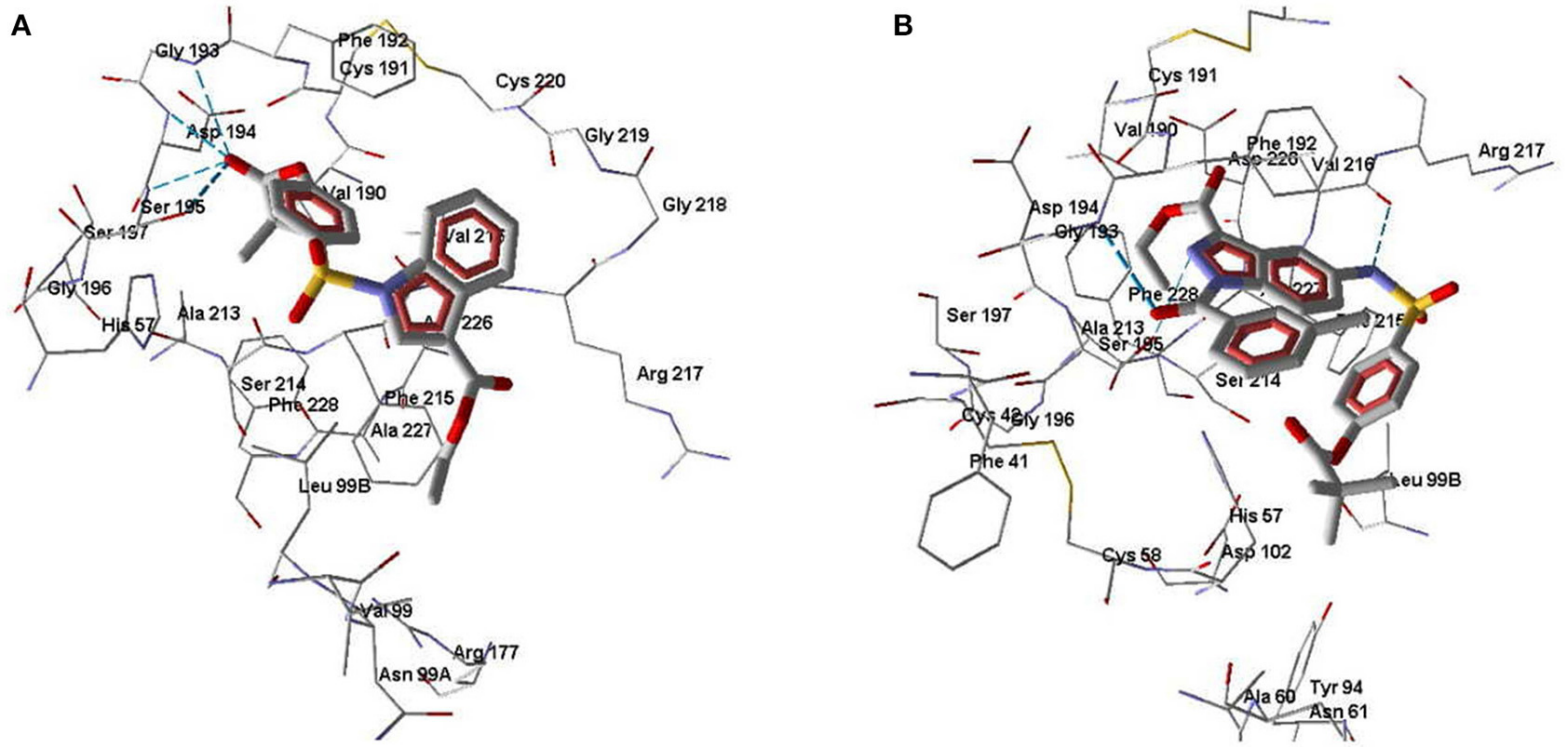
Docking pose of compounds **3a (A)** and **7e (B)**. Residues within 5 Å from the pose are visible. H-bonds are shown by blue dashed lines.

**Figure 8:**
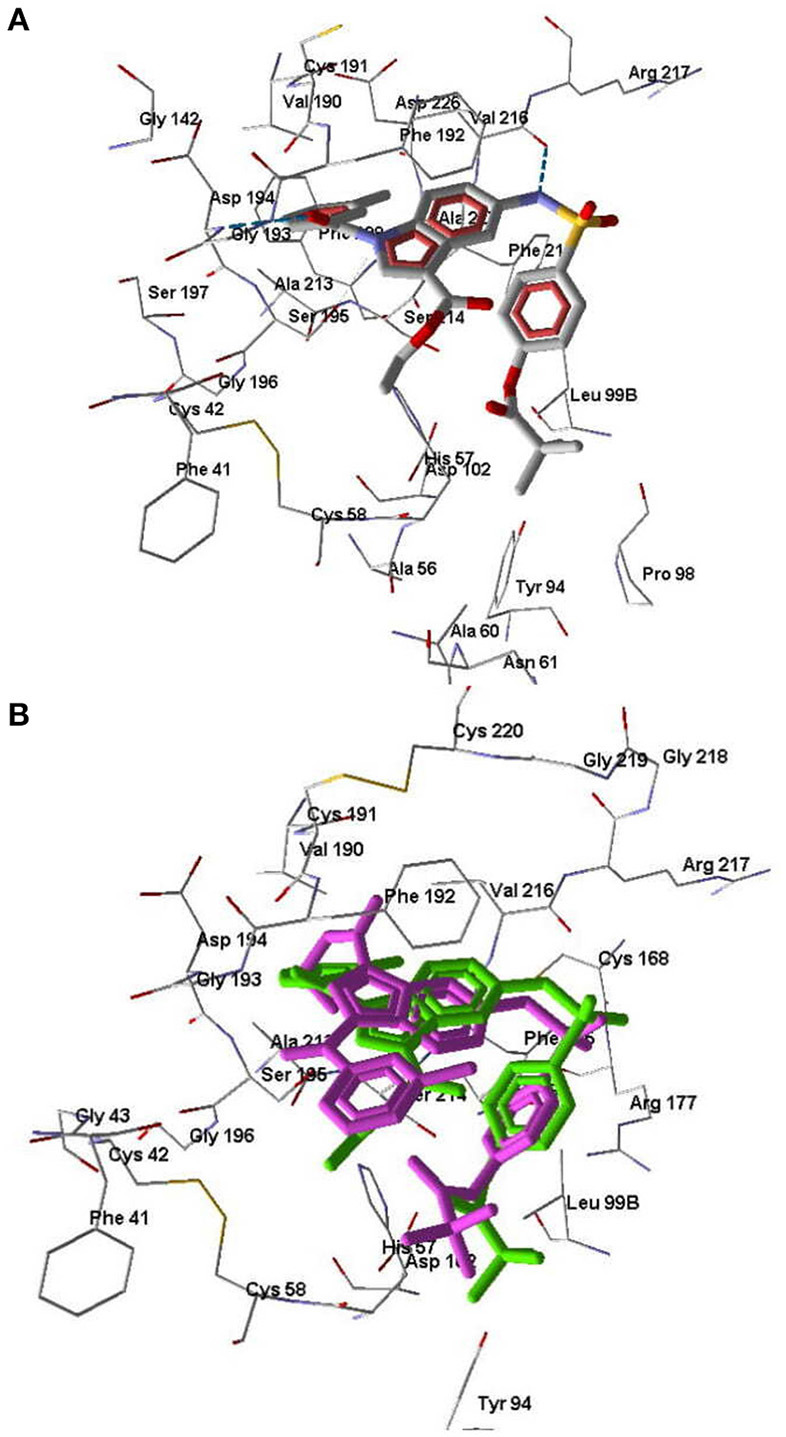
Docking poses of compounds **7b** and **7e**. **(A)** Docking pose of compounds **7b**. H-bonds are shown by blue dashed lines. **(B)** Superimposed docking poses of compounds **7e** (purple) and **7b** (green). Amino acid residues within 5 Å from the co-crystallized SEI ligand (not shown) are visible.

According to our docking results, compound **3a** forms strong H-bonds with Ser195 (two possible H-bonds), Asp194, and Gly193 with participation of the carbonyl oxygen atom of the pivaloyl group (Figure 7A). With this position in the binding site, **3a** is quite accessible for attack by Ser195 at the carbonyl carbon atom, i.e., according to the direction of metabolism proposed by Nakayama et al. (2002). The distance O(Ser195)····C=O(pivaloyl) in this case is 3.05 Å, which is comparable to the corresponding distance for Sivelestat (see above). Compound **7e** in its docking pose forms several H-bonds with HNE, one of them being a bond between the pyrazole nitrogen and Ser195 (Figure 7B). Additionally, the amide oxygen atom forms a strong Hbond with Gly193, while the sulfonamide nitrogen H-bonds with Val216. The distance O(Ser195)····C=O(pivaloyl) for the pose of compound **7e** is 5.96 Å. Thus, compound **7e** is anchored significantly within the binding site. Compound **7b** differs from **7e** by the presence of a CH group in the 5-membered ring. This reduces opportunities for the formation of H-bonds involving participation of the heterocycle. Accordingly, **7b** is slightly shifted away from Ser195 and neighboring residues (Figure 8A). Molecule **7b** is H-bonded to Val216 with participation of the sulfonamide nitrogen atom and also with Asp194 and Gly193 via participation of oxygen atom in the *m*-methylbenzoyl substituent. In Figure 8B, the poses of **7e** and **7b** are shown together. Visible amino acid residues lie within 5 Å of the co-crystallized ligand SEI. The distance O(Ser195)····C=O(pivaloyl) for the pose of **7b** in the binding site is 6.74 Å, which is the largest value of the investigated compounds (Table 5). Perhaps, due to the remoteness of the pivaloyl group from the key residue Ser195 of the catalytic triad, compound **7b** is the least active among the sulfonamides investigated.

**Table 5:** Geometric parameters of the docking poses along with biological activities of sulfonamides.

**Compound**	**O(Ser195)····C=O(pivaloyl) (Å)**	**IC_**50**_ (μM)**
**3a**	3.05	0.030
**7b**	6.74	5.2
**7d**	5.40	0.086
**7e**	5.96	0.460
**Sivelestat**	3.04	0.044

Docking scores for the obtained poses of compounds **3a**, **7b**, **7d**, **7e**, and Sivelestat anion are equal to −127.45, −108.94, −114.37, −133.75, and −125.04 MolDock units, respectively. It should be noted that these values did not show any significant correlation with IC_50_ indicating that specific protein-ligand interactions rather than total complementarity play role in appearing the inhibitory activity. Indeed, according to our results, the specific mechanism of HNE inhibition proposed by Nakayama et al. (2002), which includes the Ser195 attack on the carbonyl carbon of the pivaloyl group, can be easily achieved for compounds **3a** and Sivelestat (Table 5). The IC50 values obtained for compounds **7b**, **7d**, and **7e** are also in agreement with the geometric characteristics of their docking poses (Table 5).

## Conclusions

Previously, we demonstrated that the isoxazolone derivative **G** had high HNE inhibitory activity (Giovannoni et al., 2018) and excellent chemical stability in aqueous buffer (data not shown). Since this compound contains the 4-(sulfamoyl)phenyl pivalate fragment that is necessary for Sivelestat activity, we hypothesized that substitution of this active fragment onto other HNE inhibitor scaffolds could modulate their inhibitory activity, potentially resulting in higher efficacy and/or improved chemical stability of these new compounds. Based on this novel approach, we synthesized and characterized a number of new derivatives and demonstrated that the 4-(sulfamoyl)phenyl pivalate fragment could “replace” the role of the N-CO group at position 1 and offer a different point of attack for Ser195. Indeed, results of molecular docking of the these pivaloyl-containing compounds into the HNE binding site supported the mechanism of inhibitory activity involving a nucleophilic attack of Ser195 from the catalytic triad onto the carbonyl group of the pivaloyl moiety. Clearly, the selected scaffolds with adequate substituents can be appropriate carriers for the Sivelestat pharmacophore since many of the new compounds had high inhibitory activity in the nanomolar range, with the most potent inhibitors being **3a, 3b, 3f, 3g**, and **9a** (IC_50_= 19–30 nM). However, these data also indicate that this strategy does not produce an expected additive effect of inhibitor potency, probably due to increased steric hindrance of the pivaloyl substituent.

## Data Availability Statement

All datasets presented in this study are included in the article/Supplementary Material.

## Author Contributions

LC and MG designed the compounds and wrote the manuscript. NC, GG, and CV synthesized the compounds and checked the final version of the manuscript. IS and MQ performed *in vitro* studies (inhibition essay), analyzed the stability of compounds and wrote the pharmacological section. AK performed molecular modeling studies. All of the authors have given approval to the final version of the manuscript.

## Conflict of Interest

The authors declare that the research was conducted in the absence of any commercial or financial relationships that could be construed as a potential conflict of interest.
