# Supplementary material for: Novel Sulfonamide Analogs of Sivelestat as Potent Human Neutrophil Elastase Inhibitors
Source: Front Chem. 2020 Sep 1;8:795. doi: 10.3389/fchem.2020.00795 (PMC7491426; doi:10.3389/fchem.2020.00795)
Supplement: Supplementary file 1 [file Data_Sheet_1.docx]

**Supplementary Material**

**For**

## Novel Sulfonamide Analogs of Sivelestat as Potent Human Neutrophil Elastase Inhibitors

**Letizia Crocetti^1^, Maria Paola Giovannoni^1*^, Niccolò Cantini^1^, Gabriella Guerrini^1^, Claudia Vergelli^1^, Igor A. Schepetkin^2^, Andrei I. Khlebnikov^3^, and Mark T. Quinn^2^**

^1^Neurofarba, Pharmaceutical and Nutraceutical Section, University of Florence, Sesto Fiorentino, Italy

^2^Department of Microbiology and Immunology, Montana State University, Bozeman, Montana

^3^Kizhner Research Center, Tomsk Polytechnic University, Tomsk, Russia

***Corresponding Author**

Prof. Maria Paola Giovannoni

Department of Neurofarba,

University of Florence, Via Ugo Schiff 6,

Sesto Fiorentino, Firenze 50019, Italy.

Tel. +39 055 4573682

Email: [mariapaola.giovannoni@unifi.it](mailto:mariapaola.giovannoni@unifi.it)

**Table of contents**

1. ^1^H -NMR and ^13^C- NMR spectra of compounds **3a-g**, **3i-l**, **4**, **7a-e**, **9a**,**b** and **11**.
2. Elemental analysis (**Table S1**).
3. Docking pose of SEI molecule (Figure S1).

**
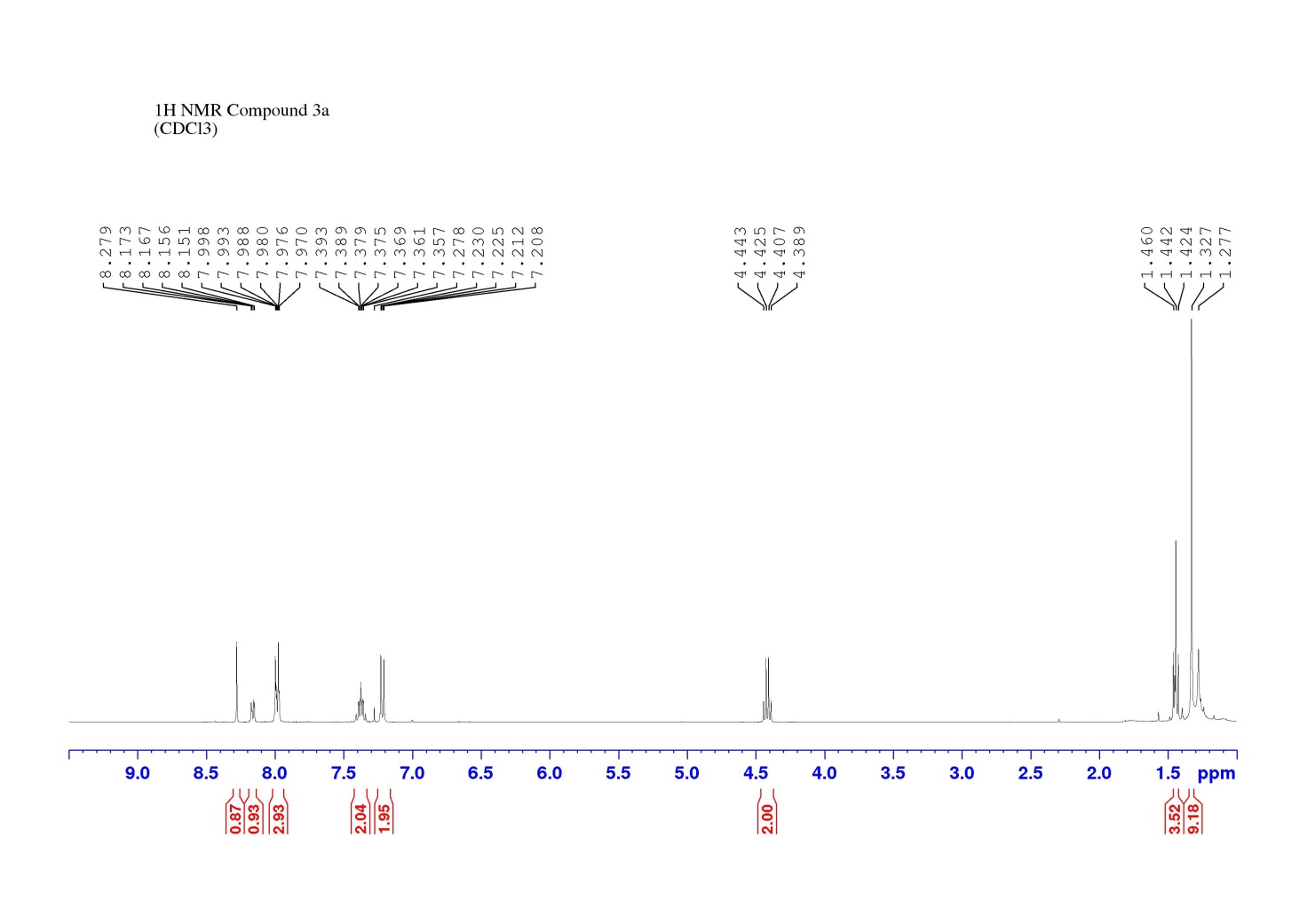
**

**
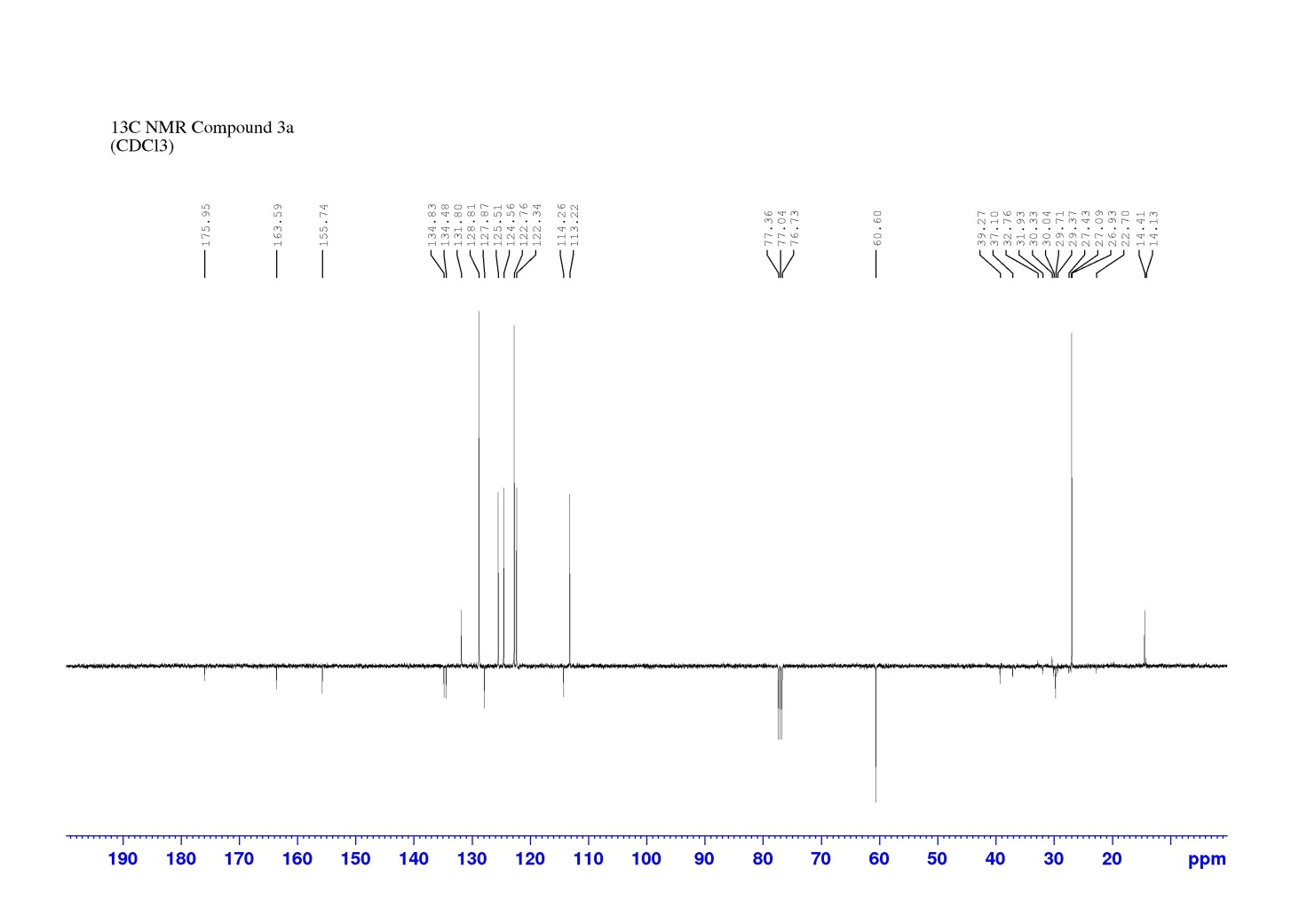
**

**
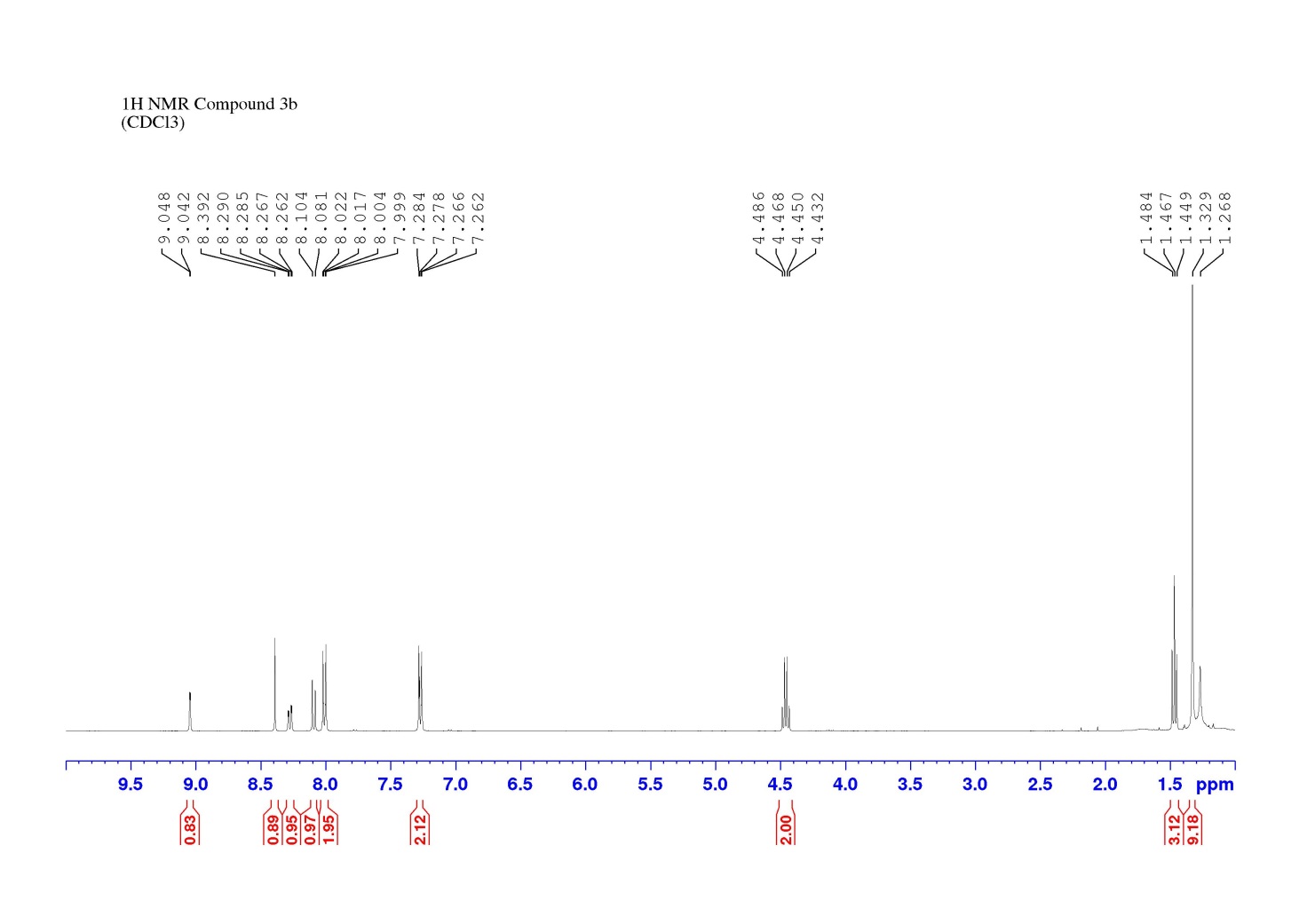
**

**
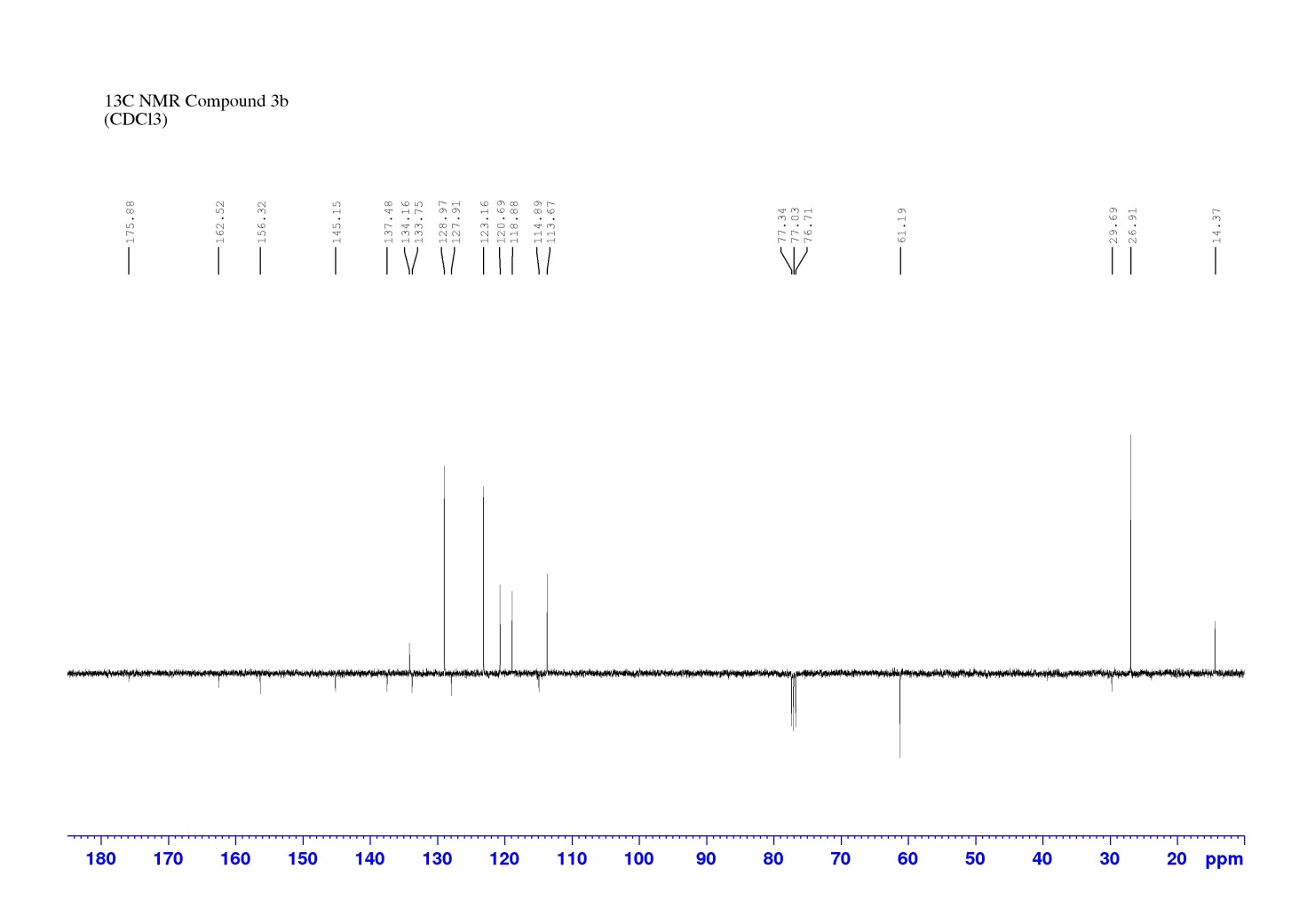
**

**
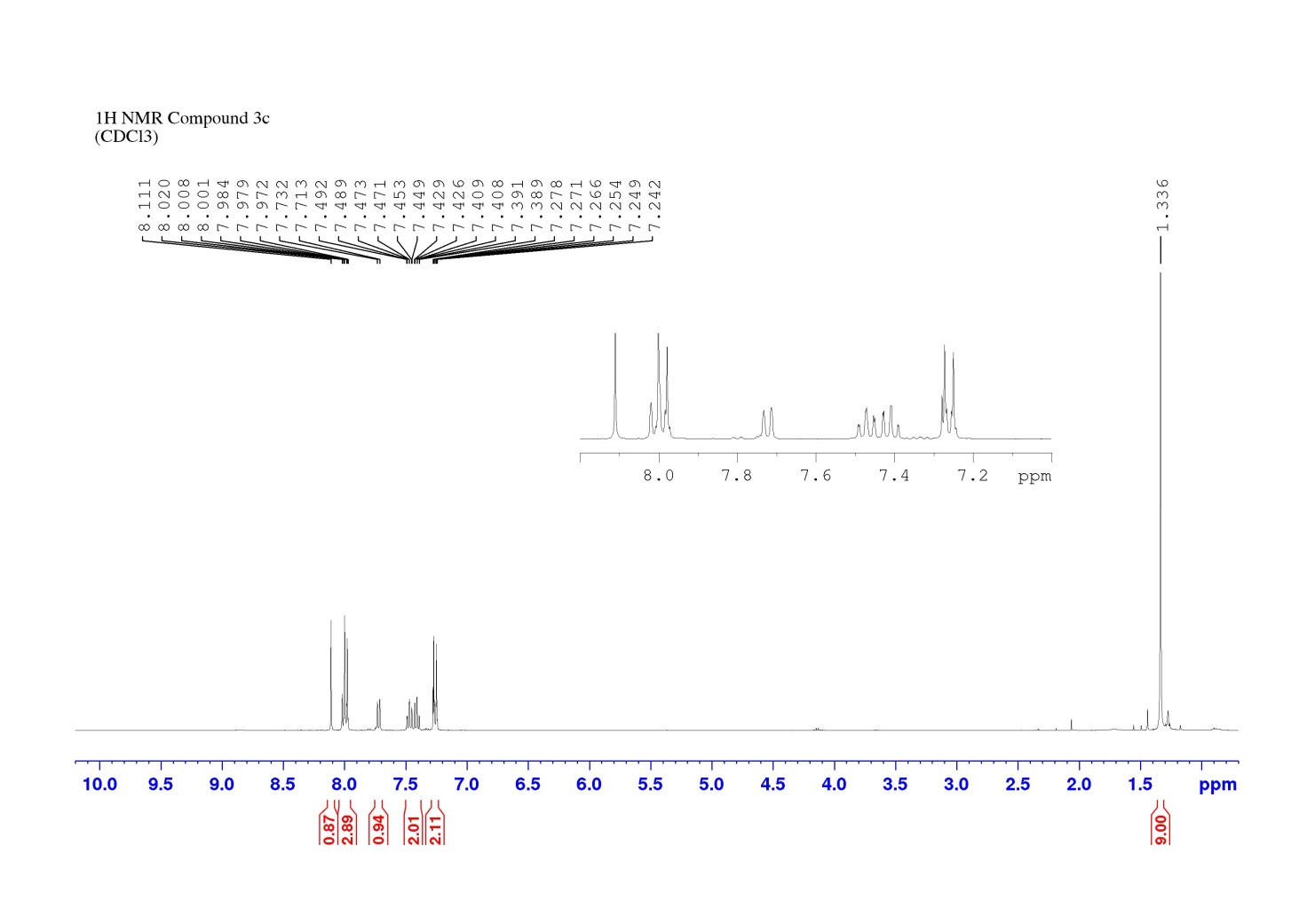
**

**
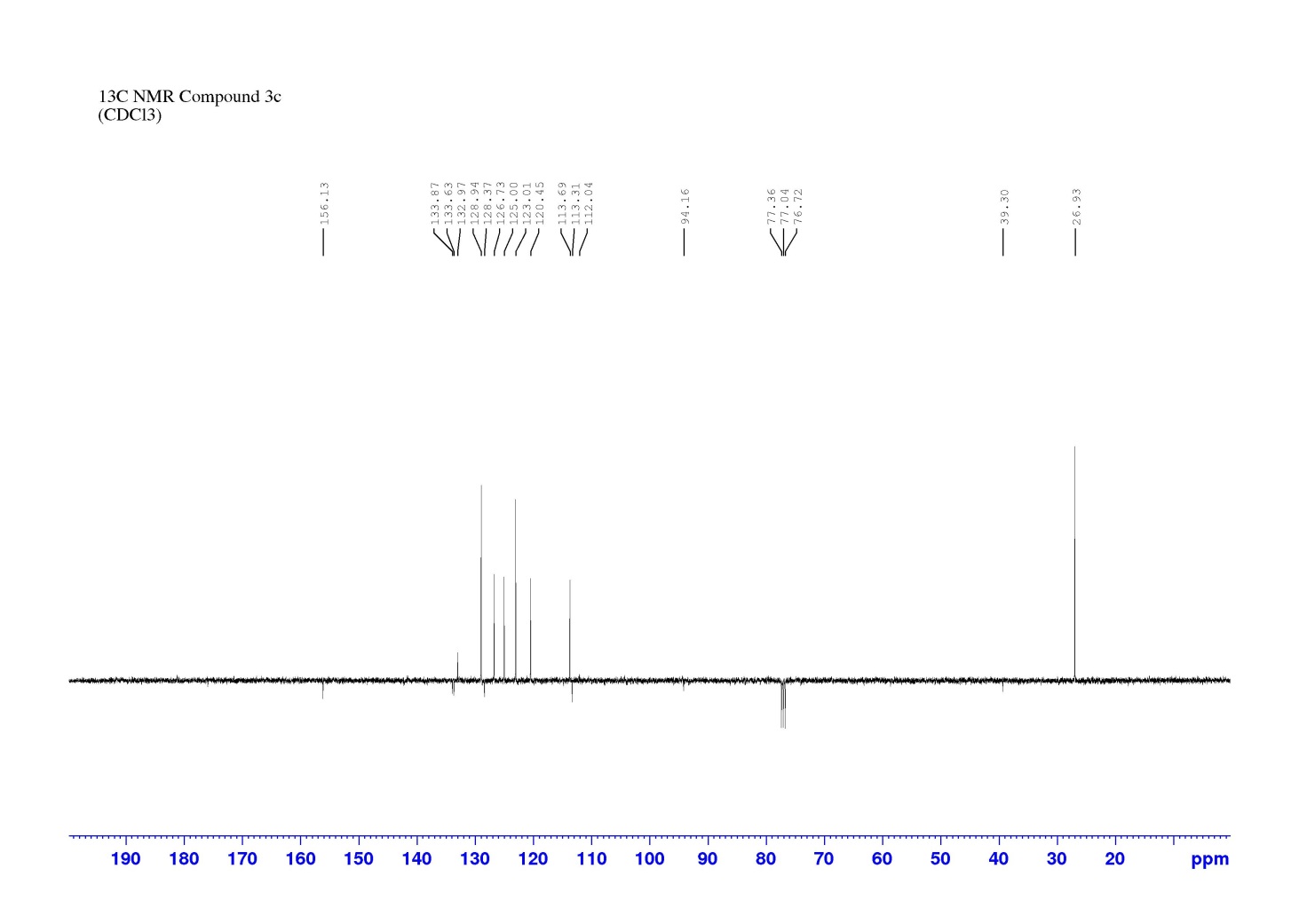
**

**
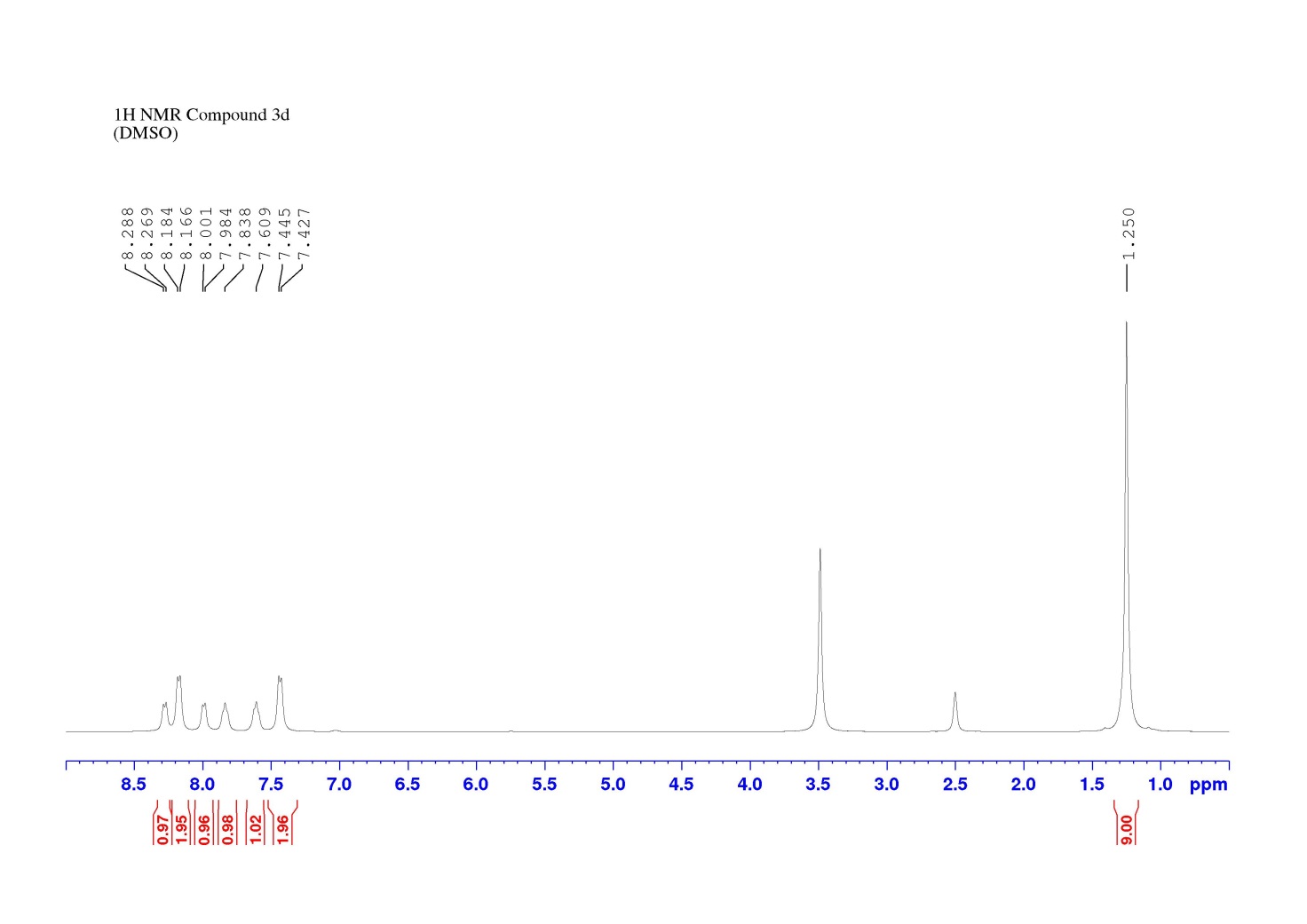
**

**
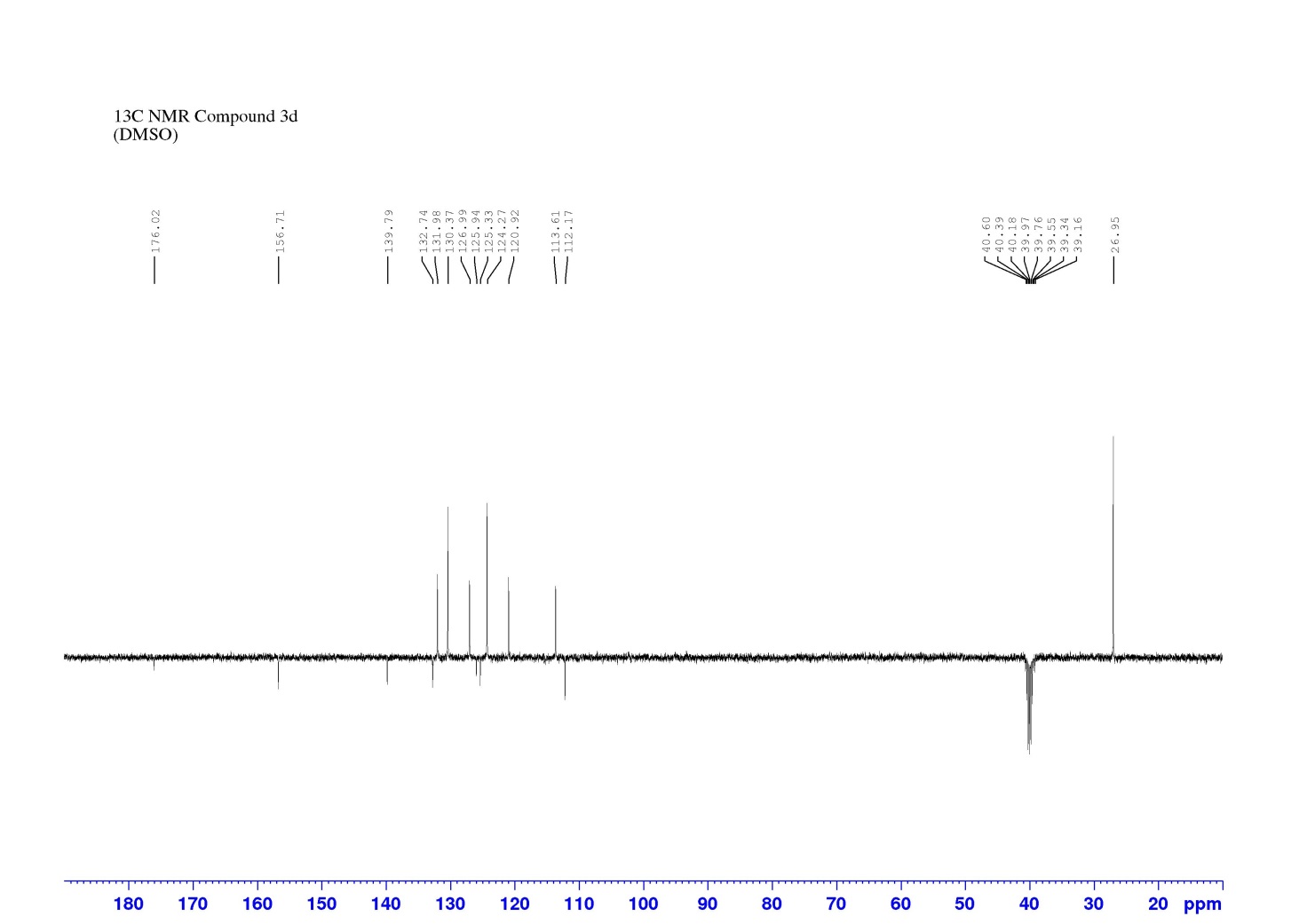
**

**
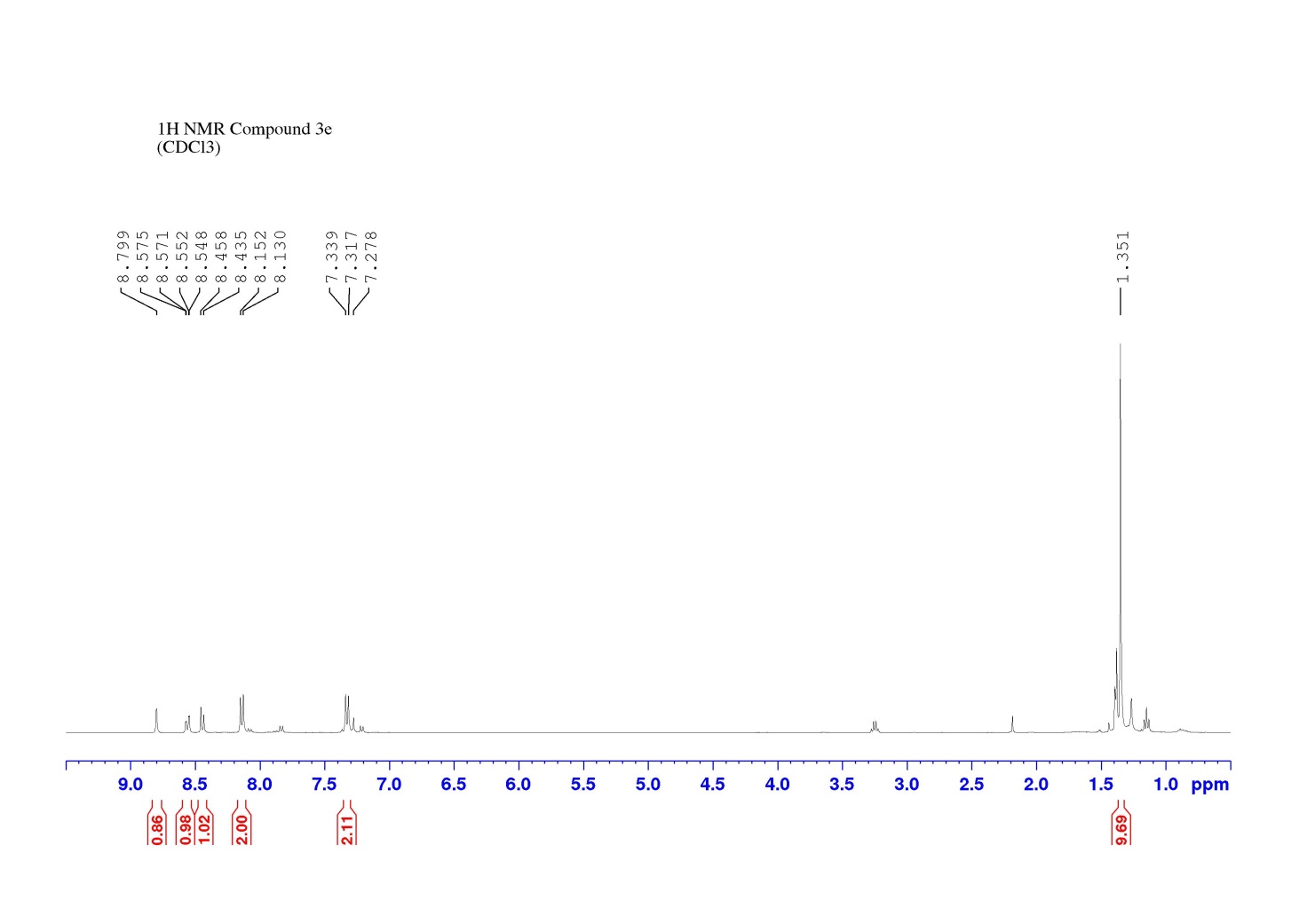
**

**
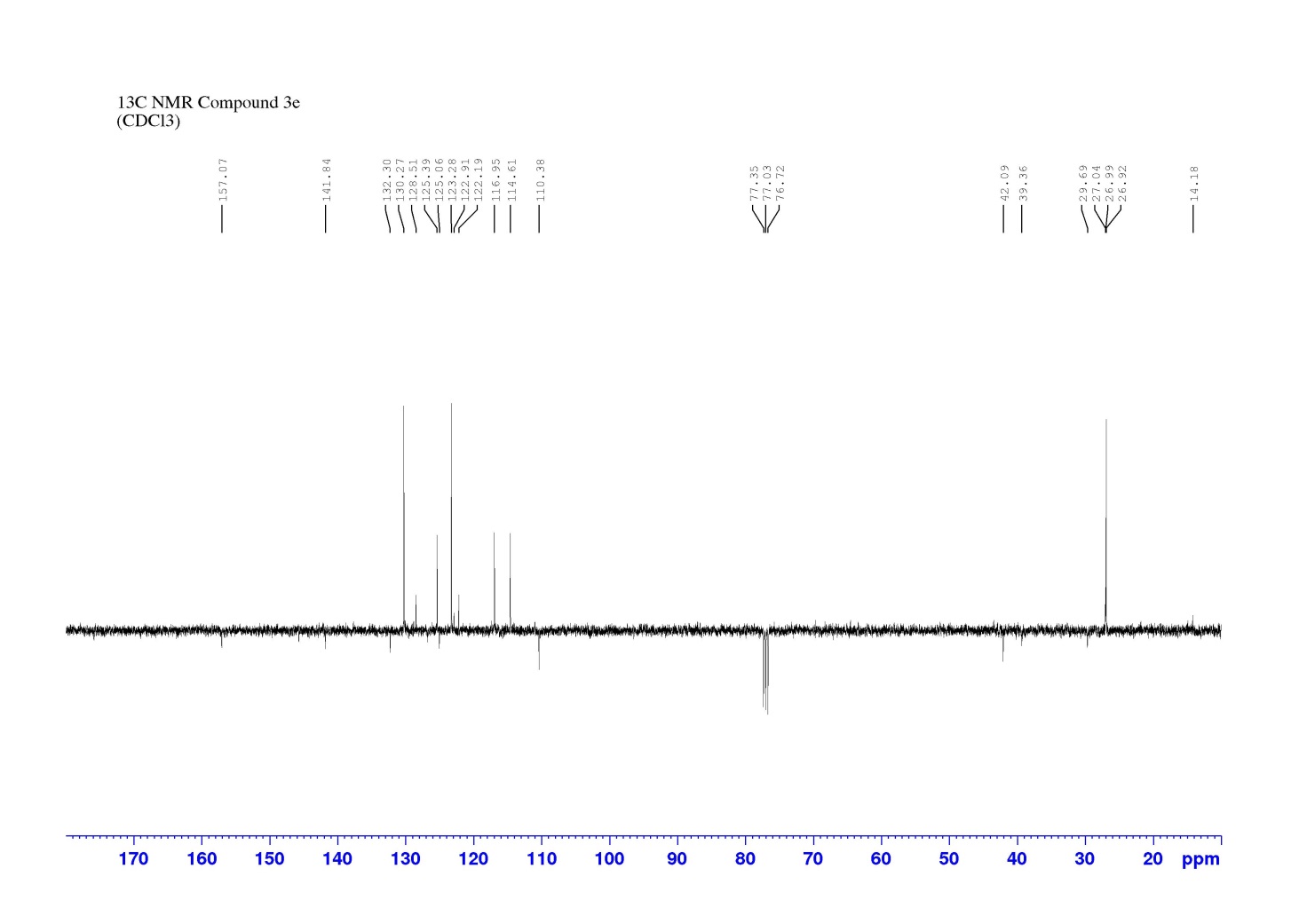
**

**
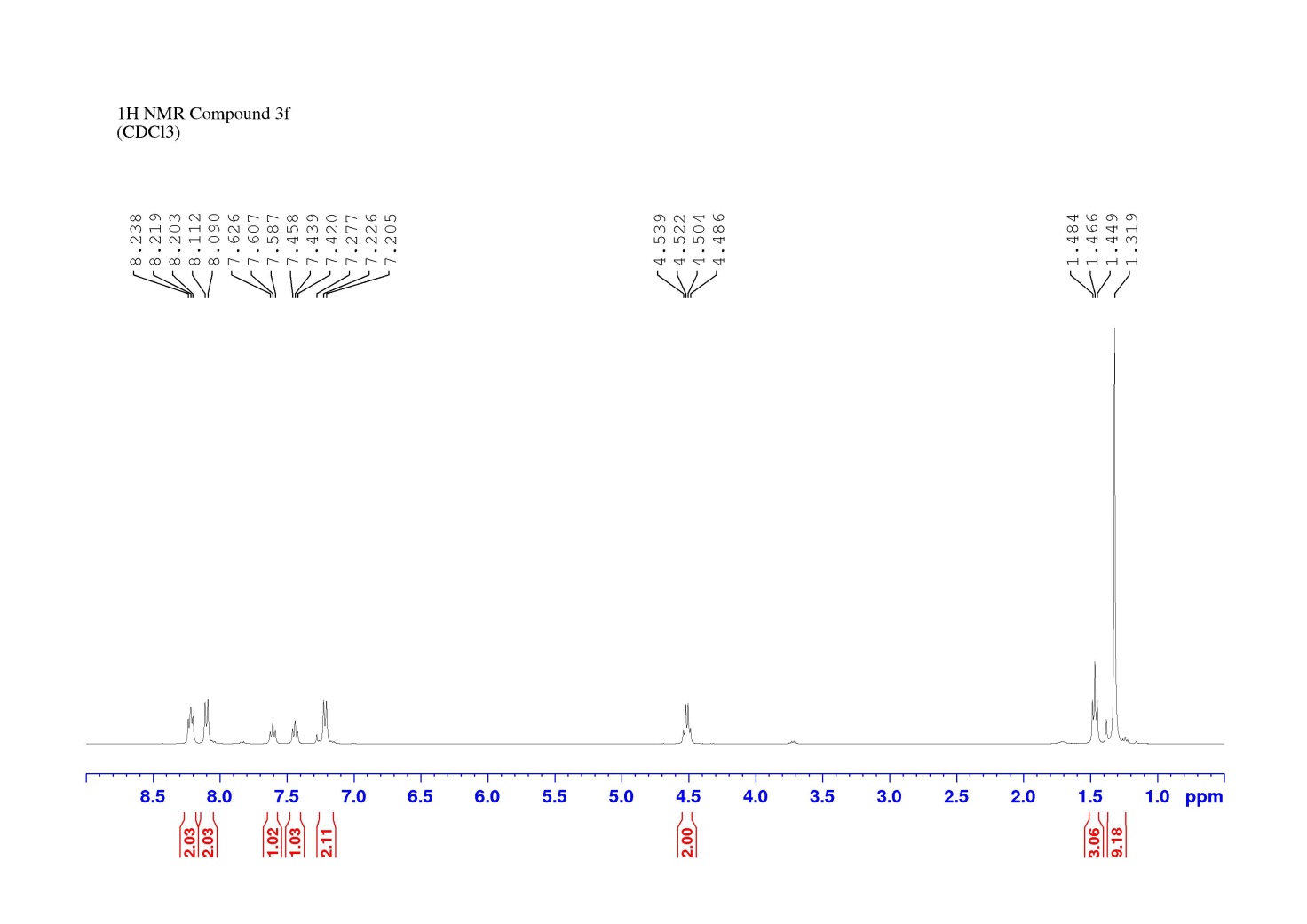
**

**
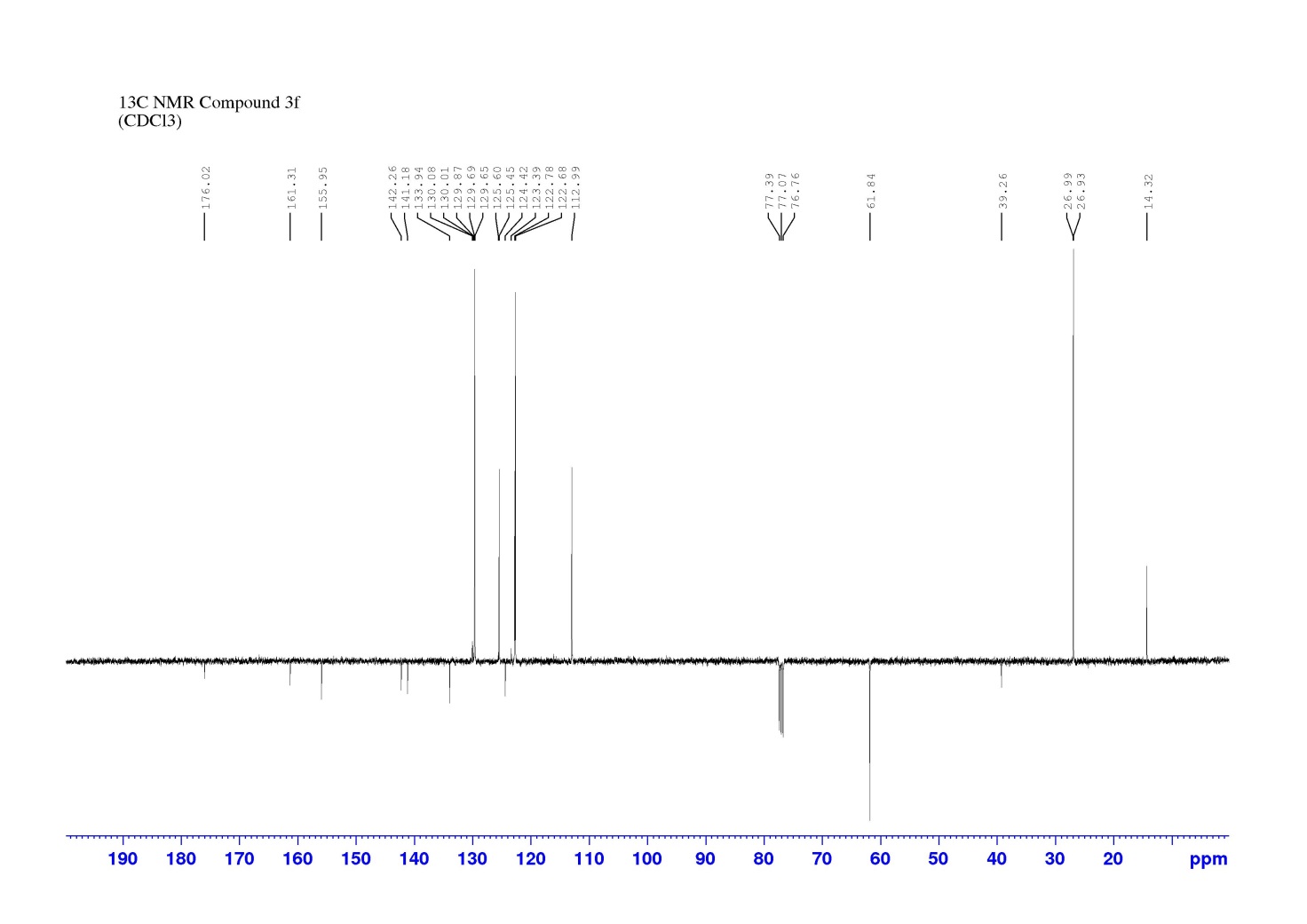
**

**
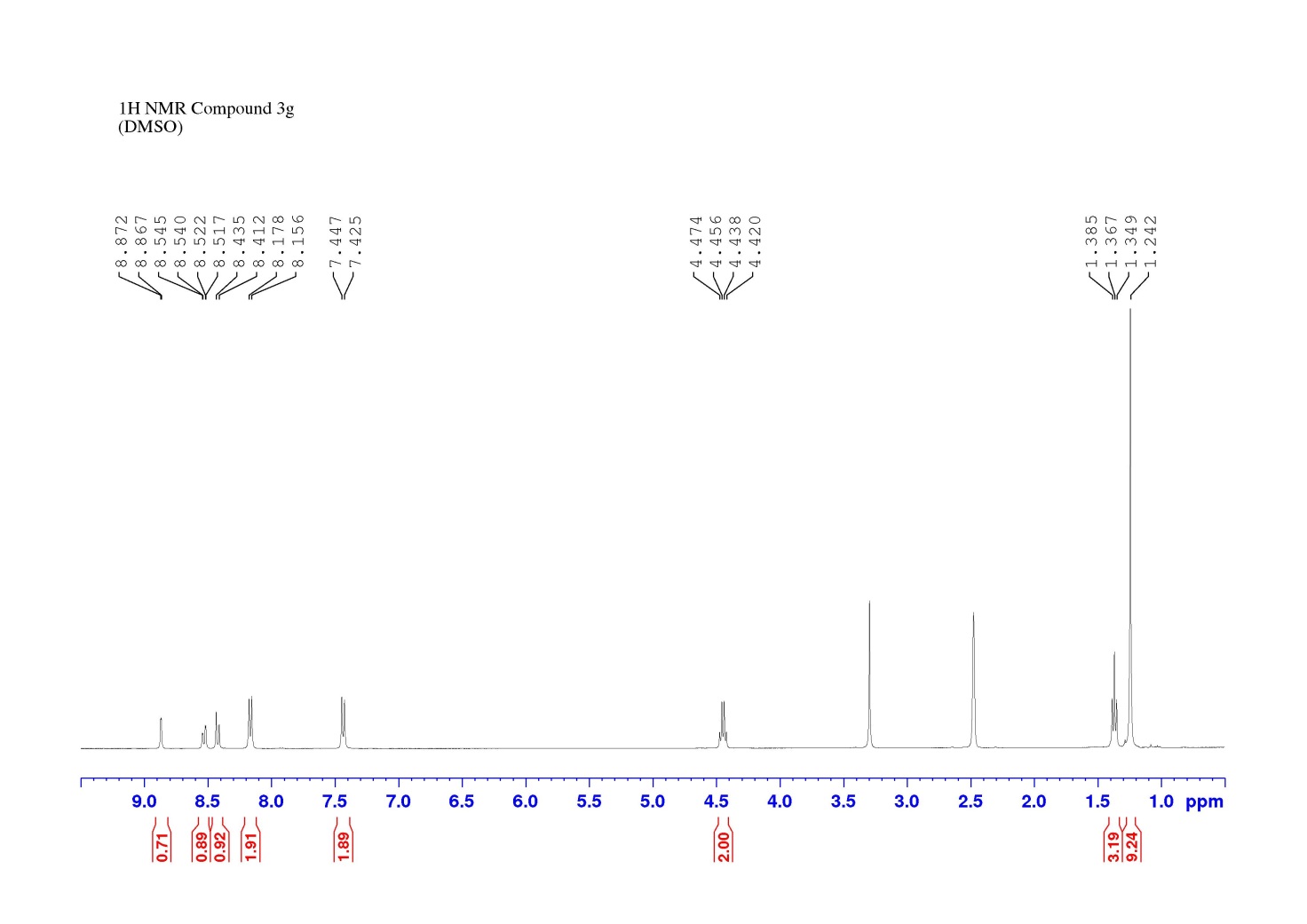
**

**
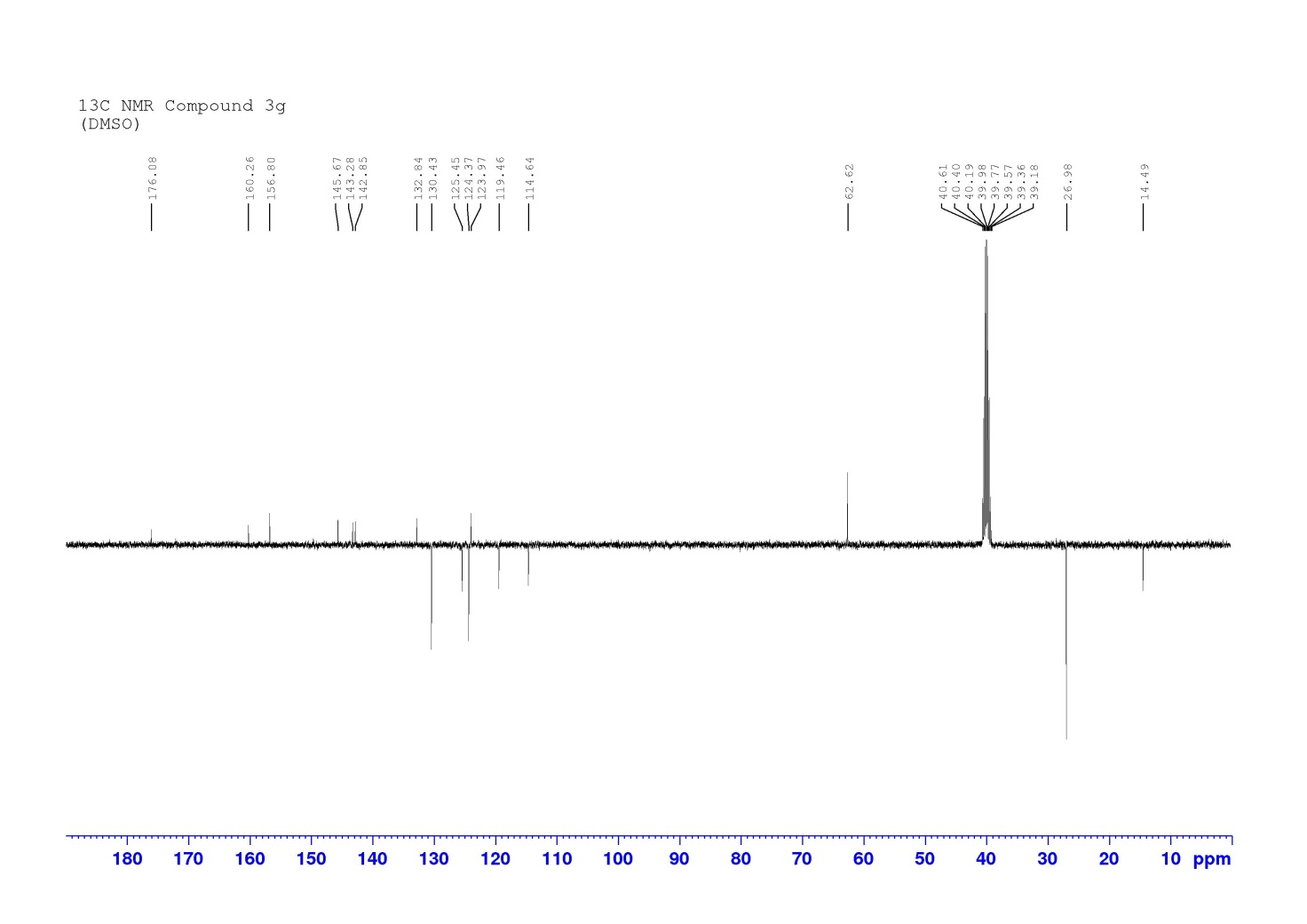
**

**
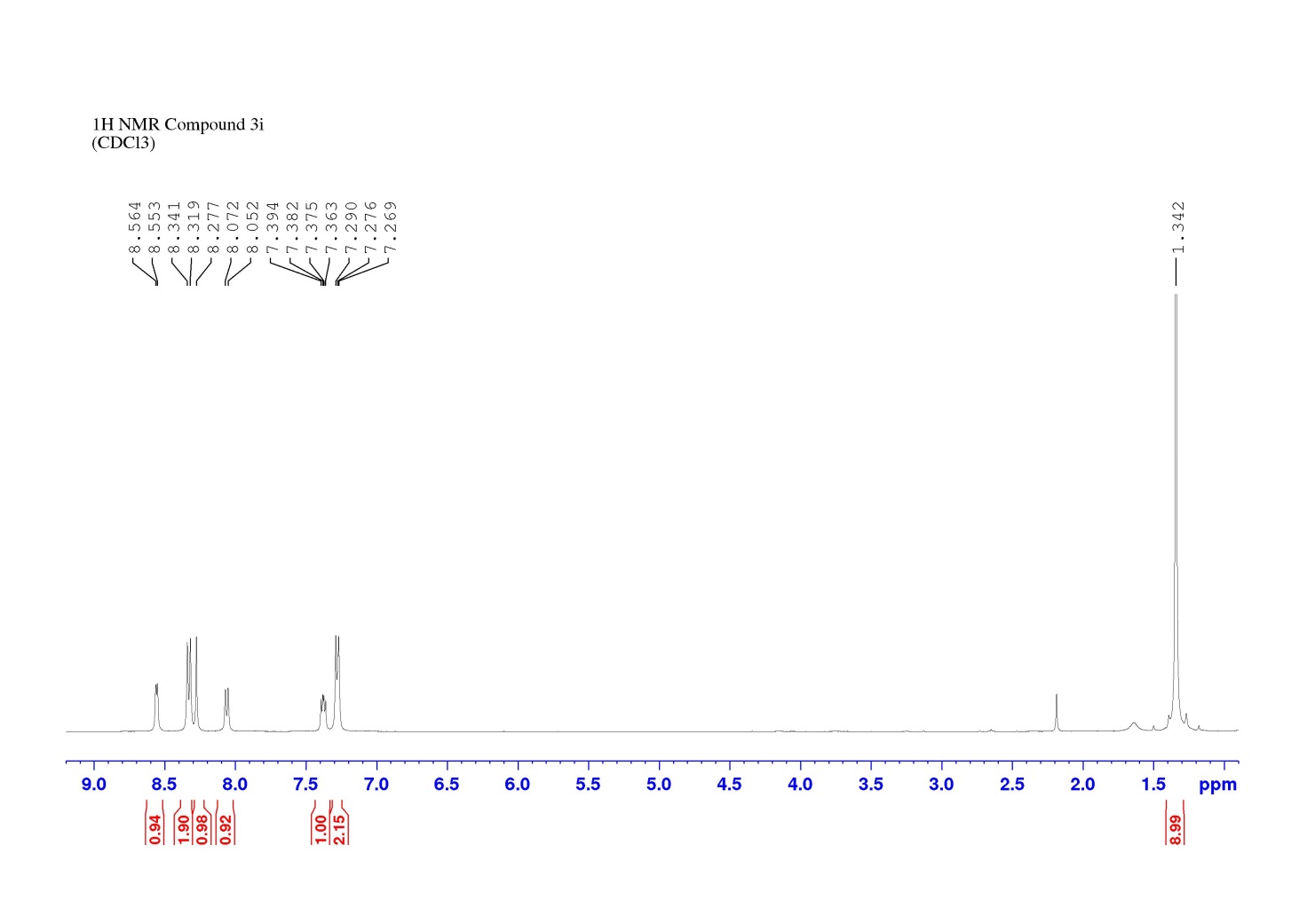
**

**
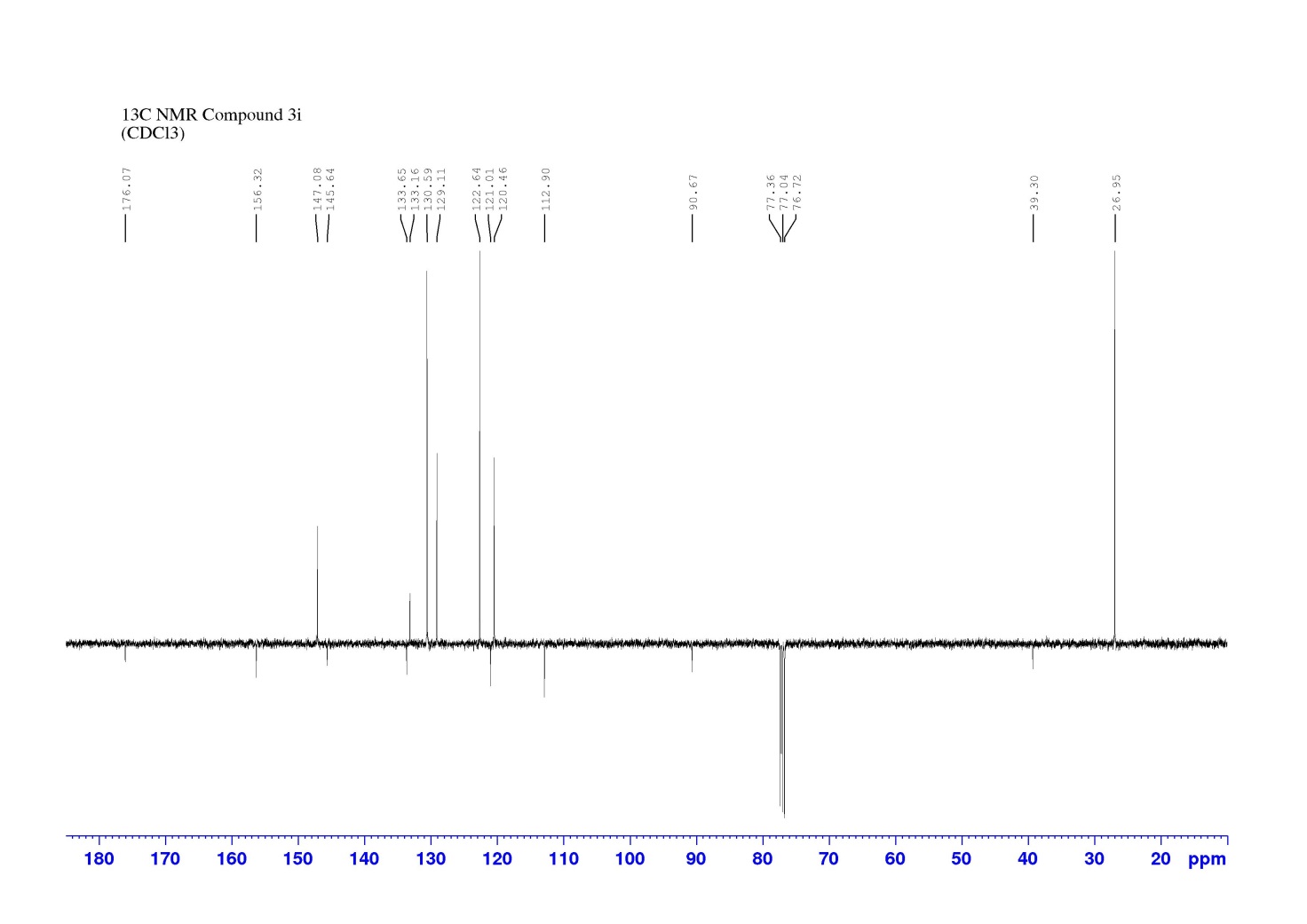
**

**
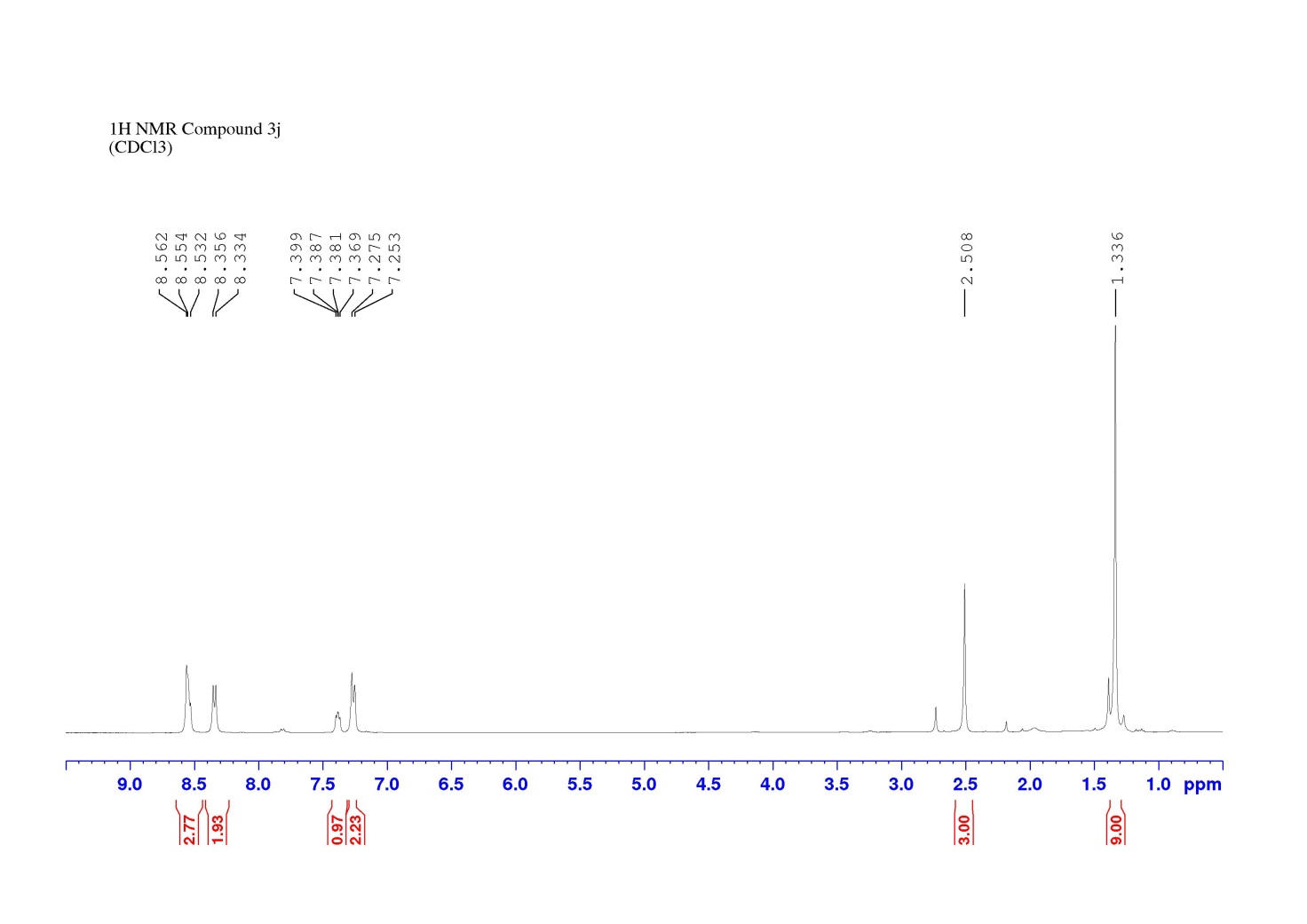
**

**
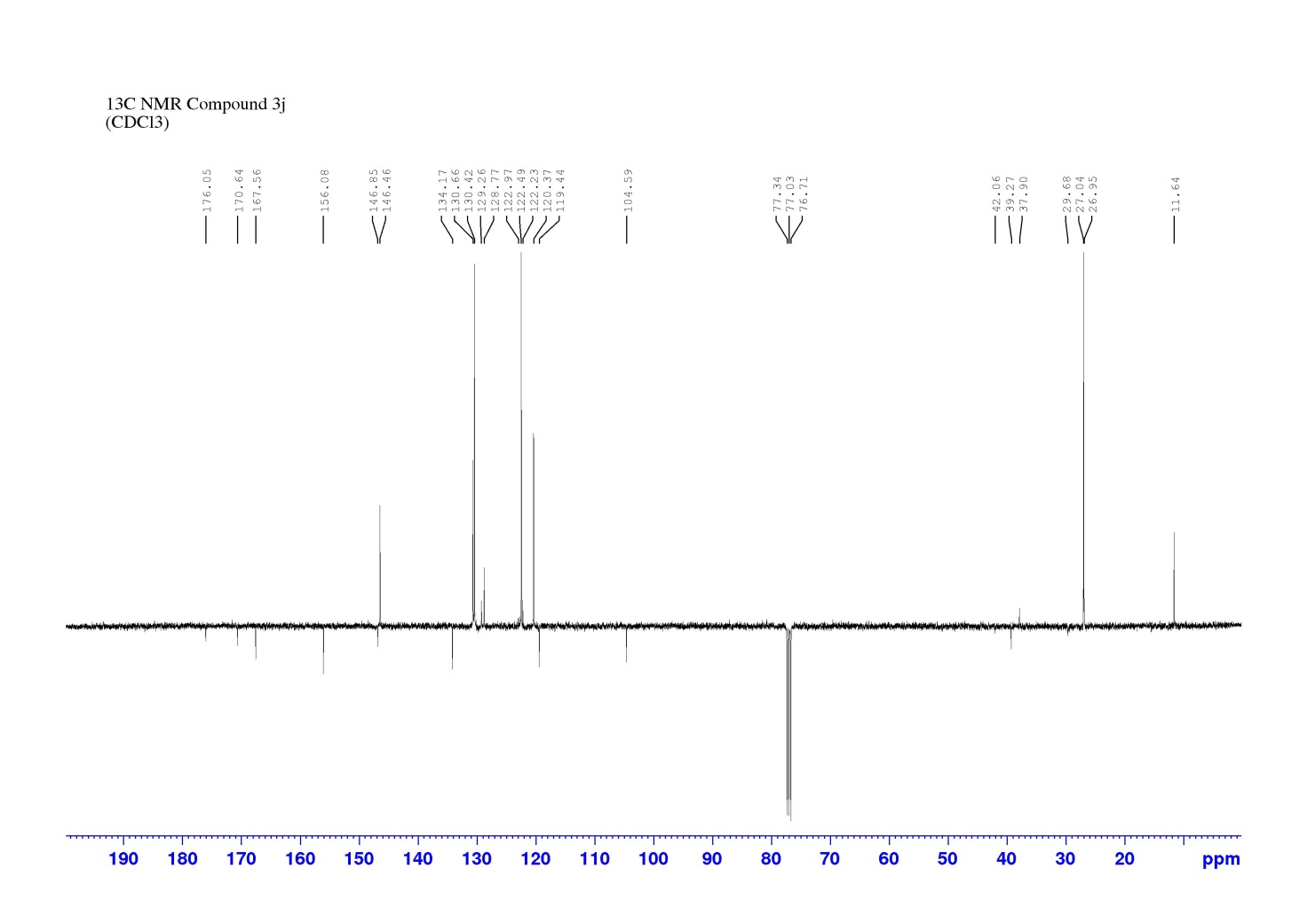
**

**
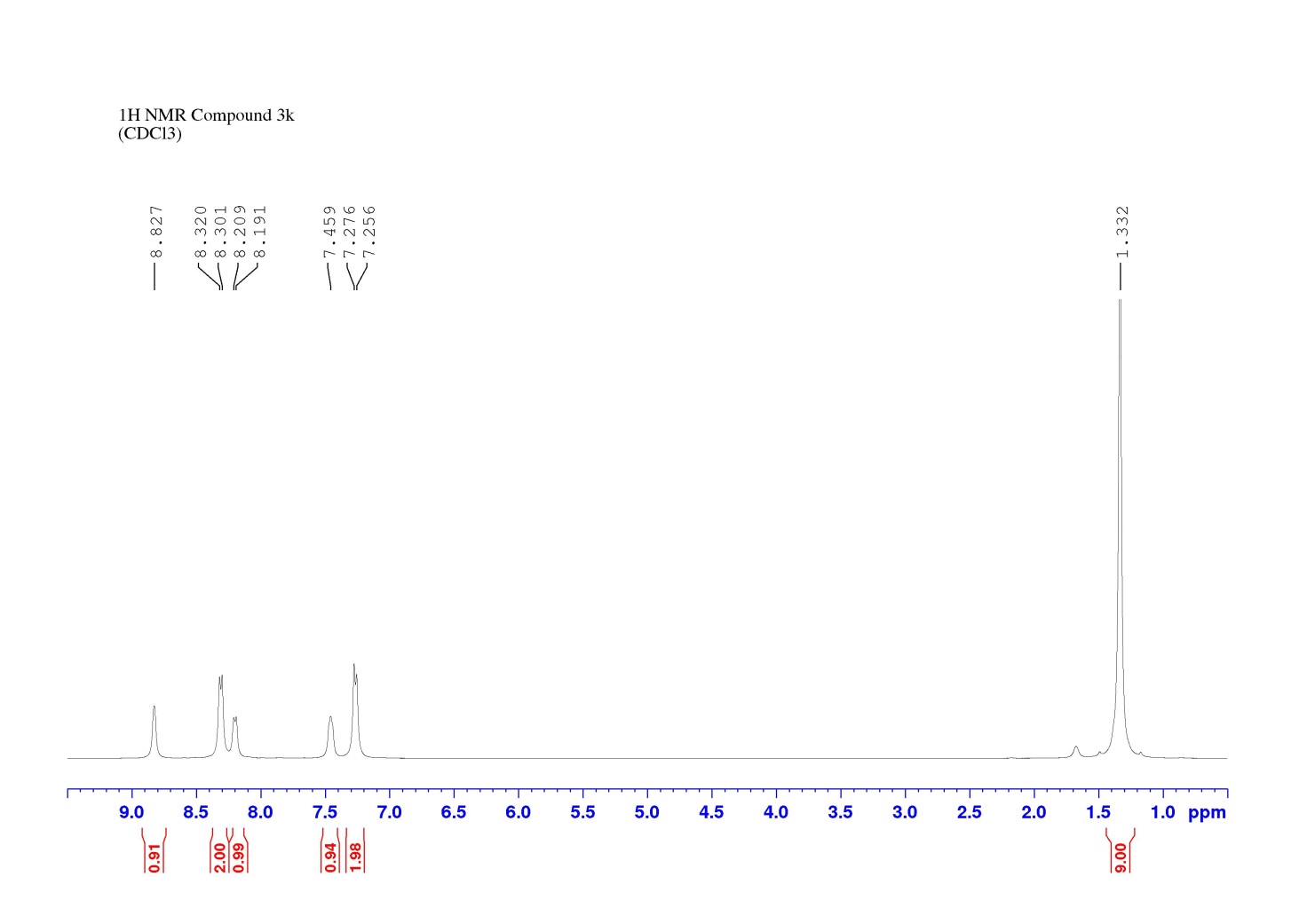
**

**
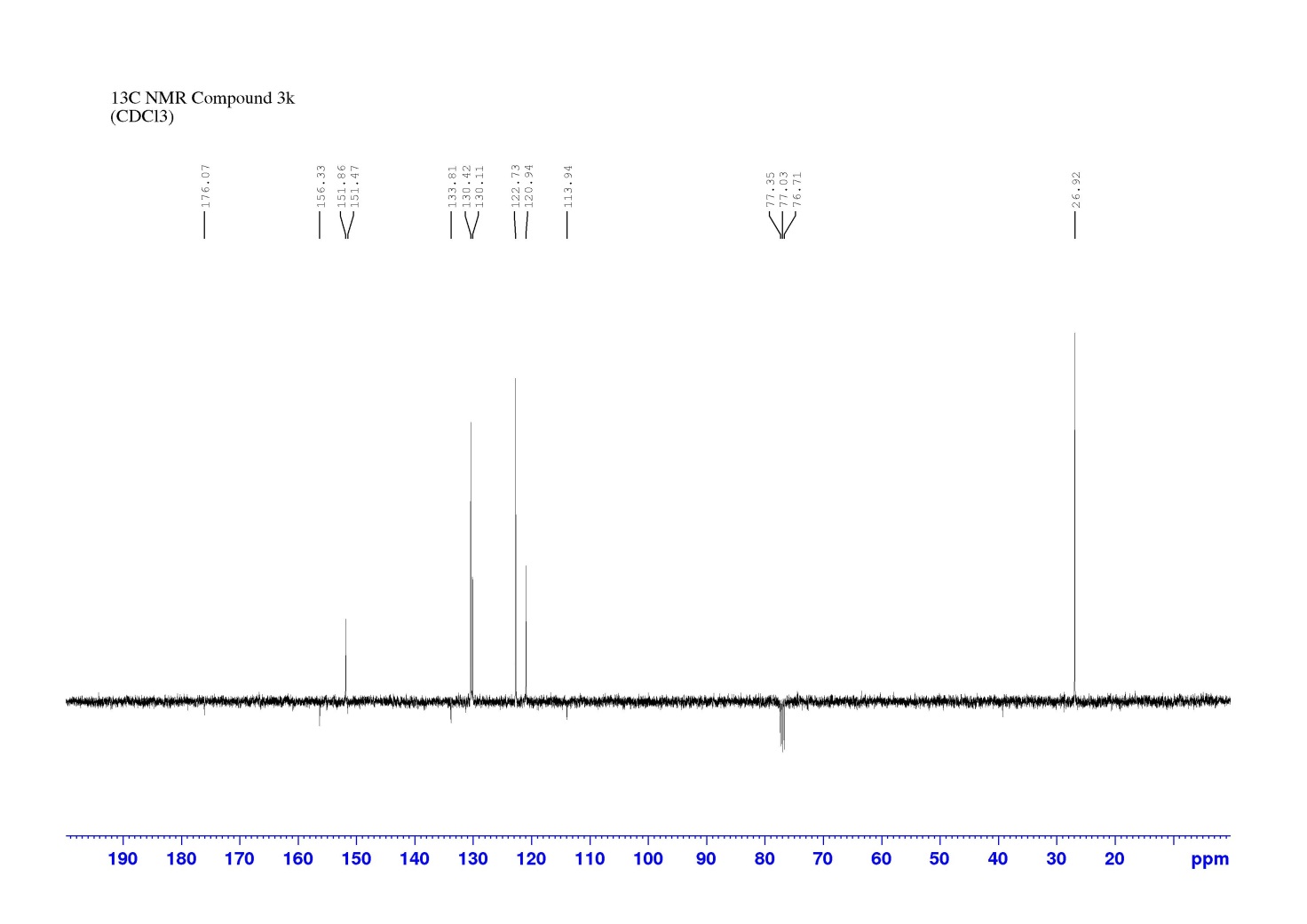
**

**
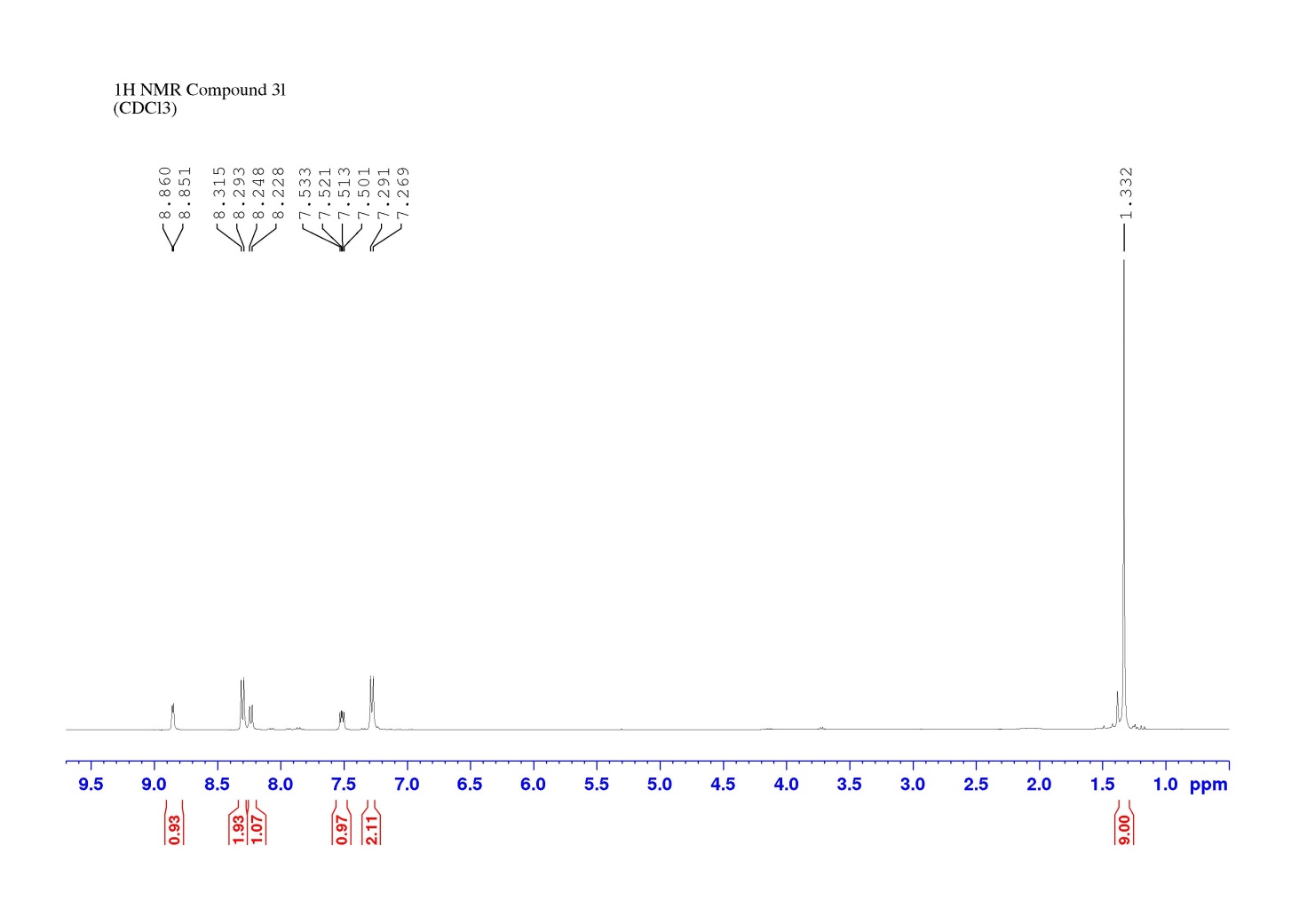
**

**
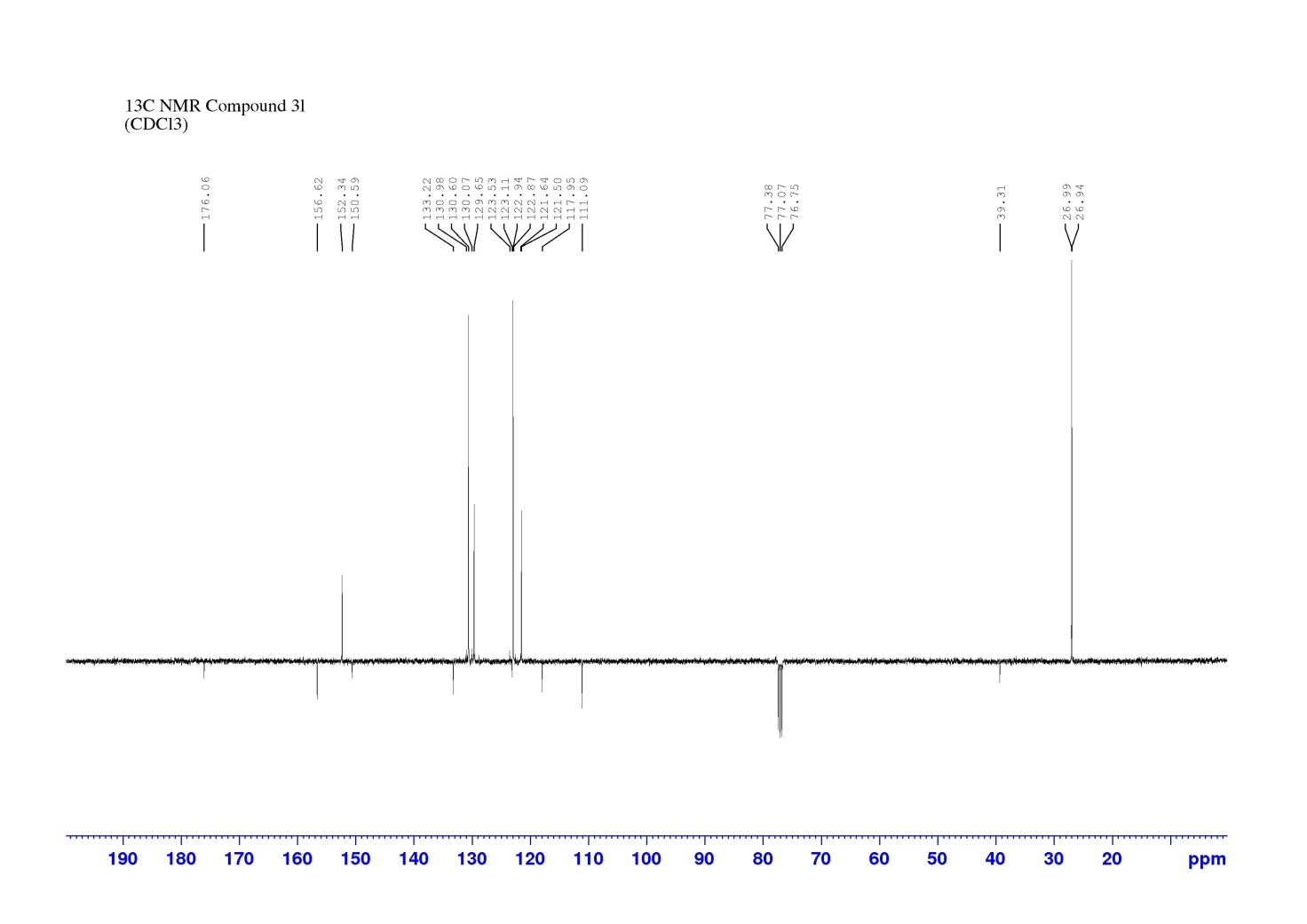
**

**
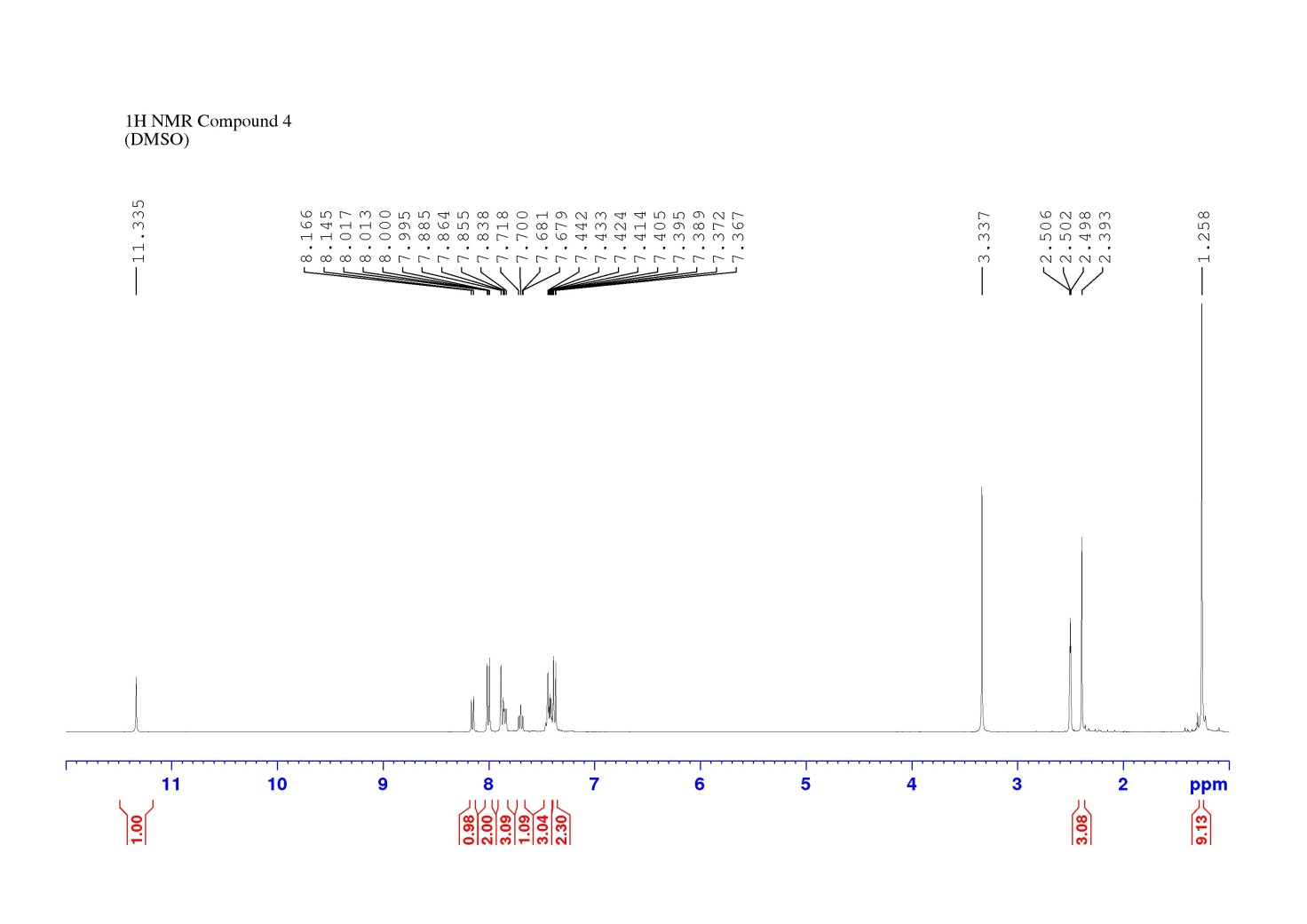
**

**
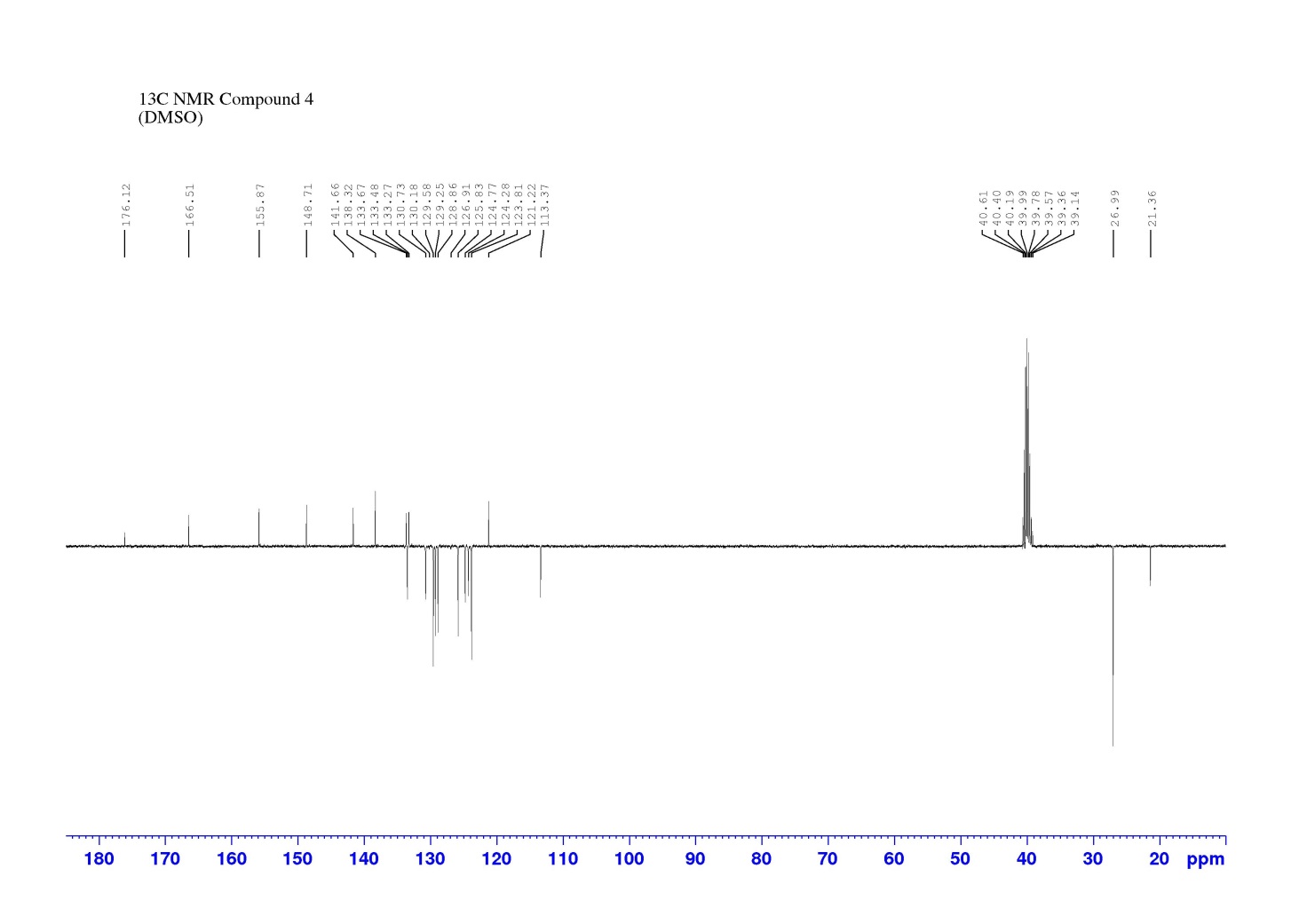
**

**
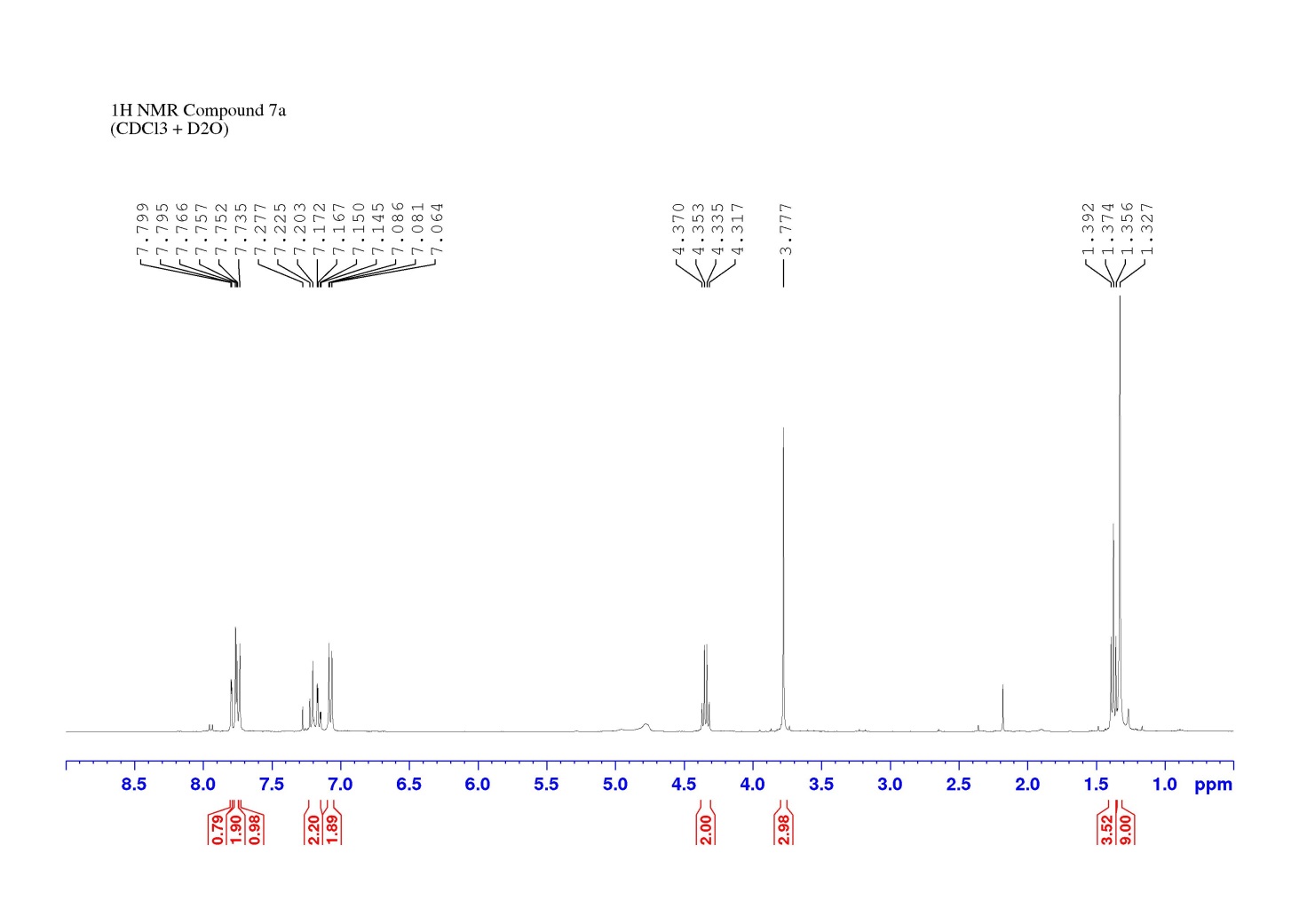
**

**
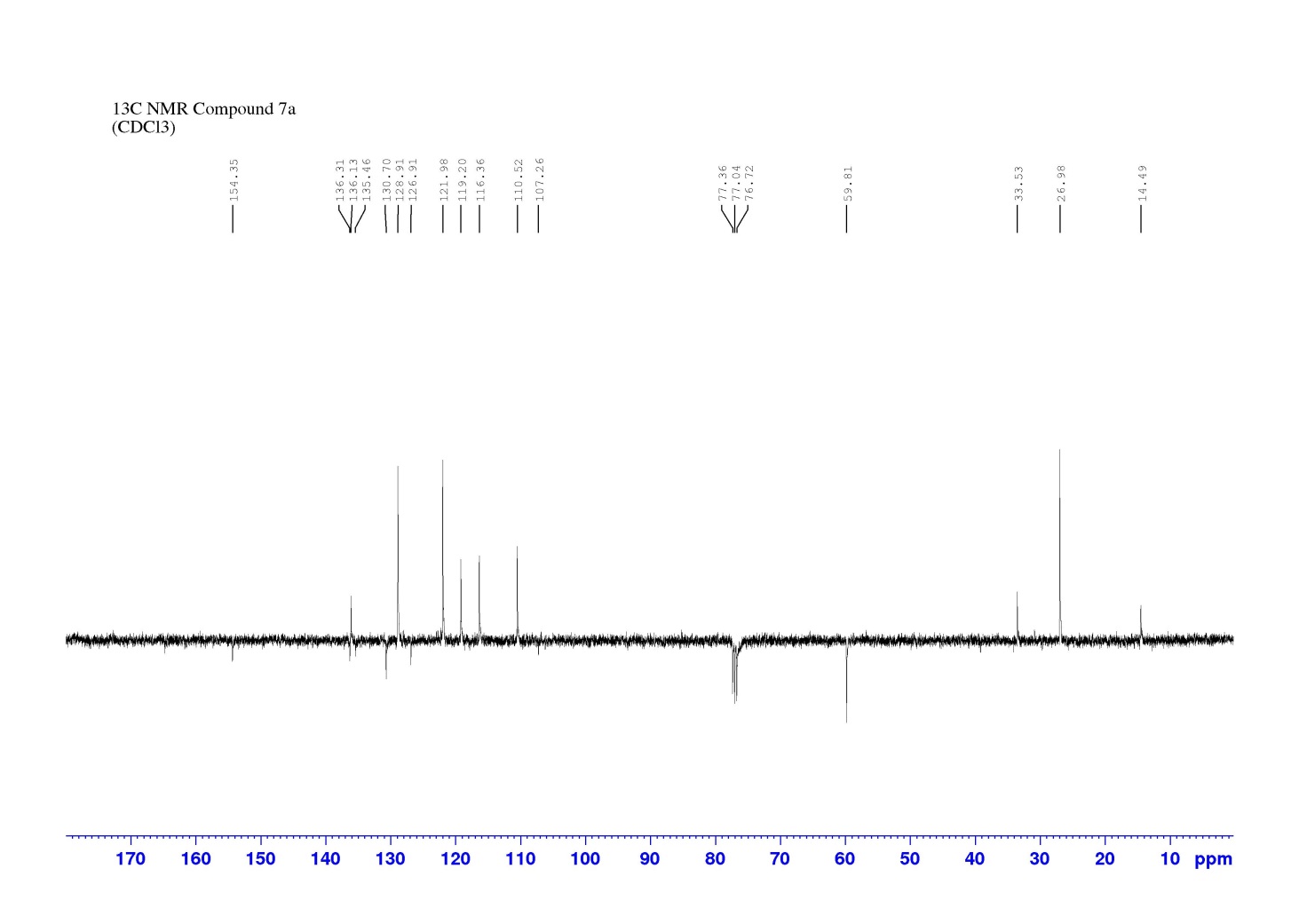
**

**
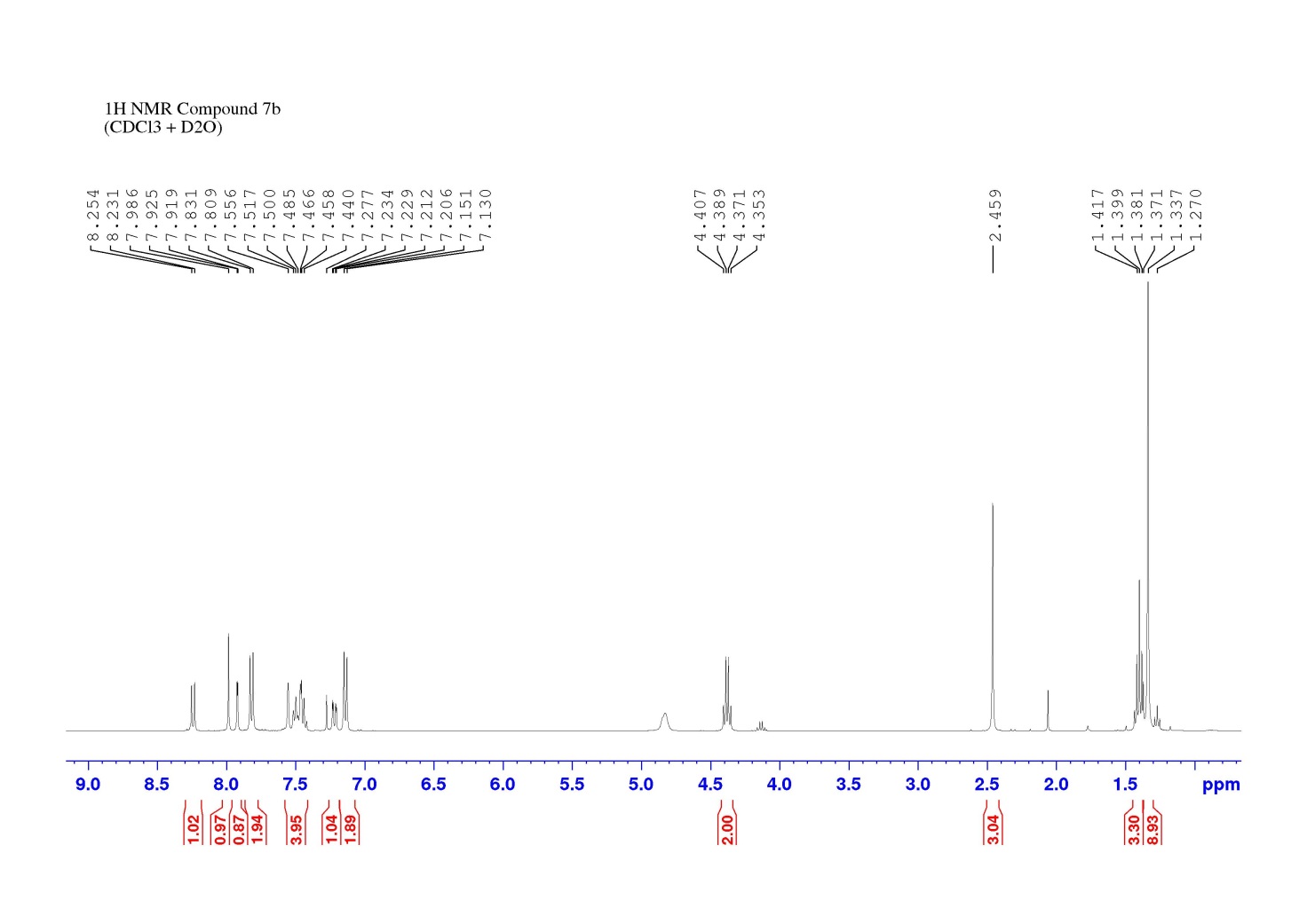
**

**
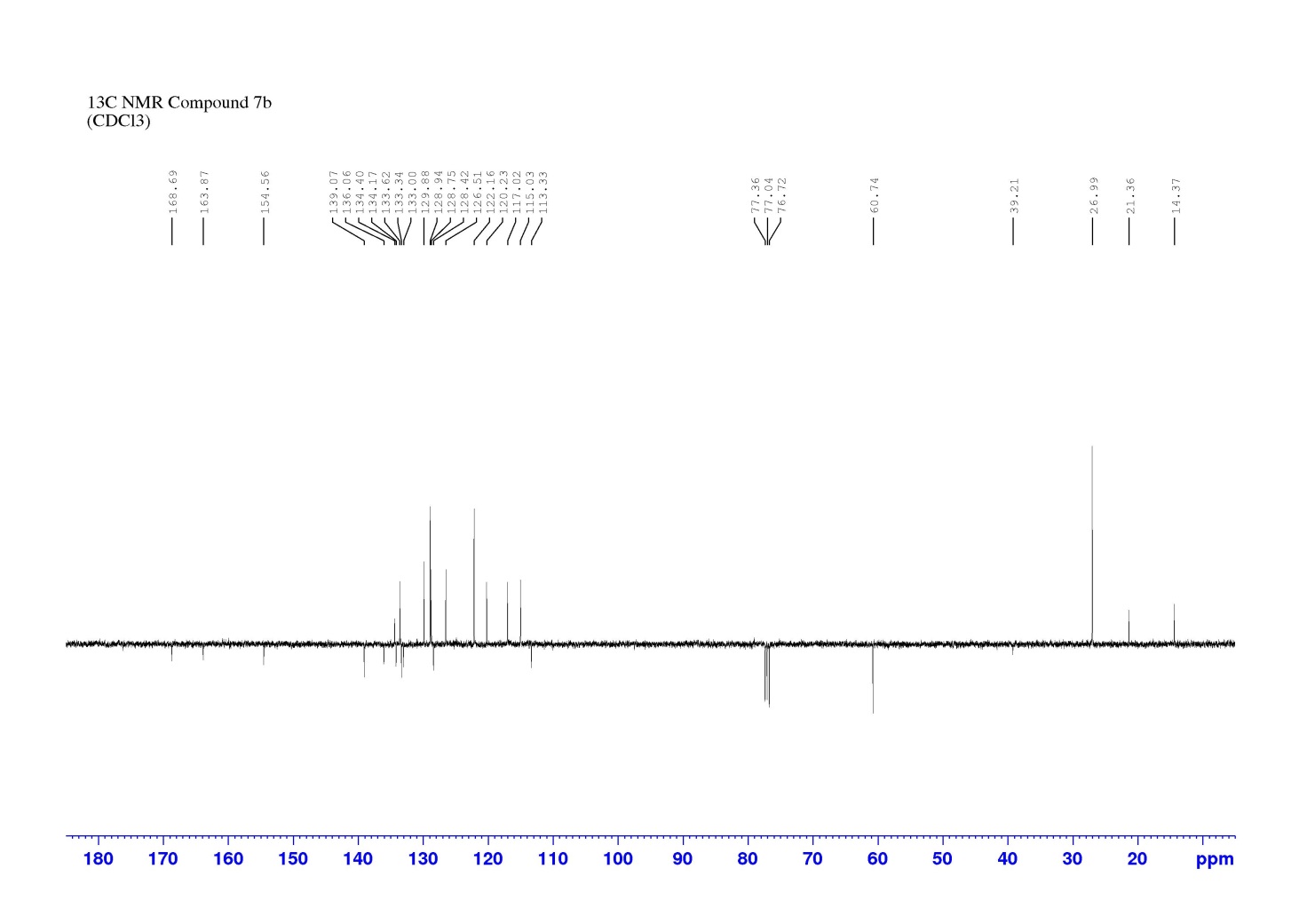
**

**
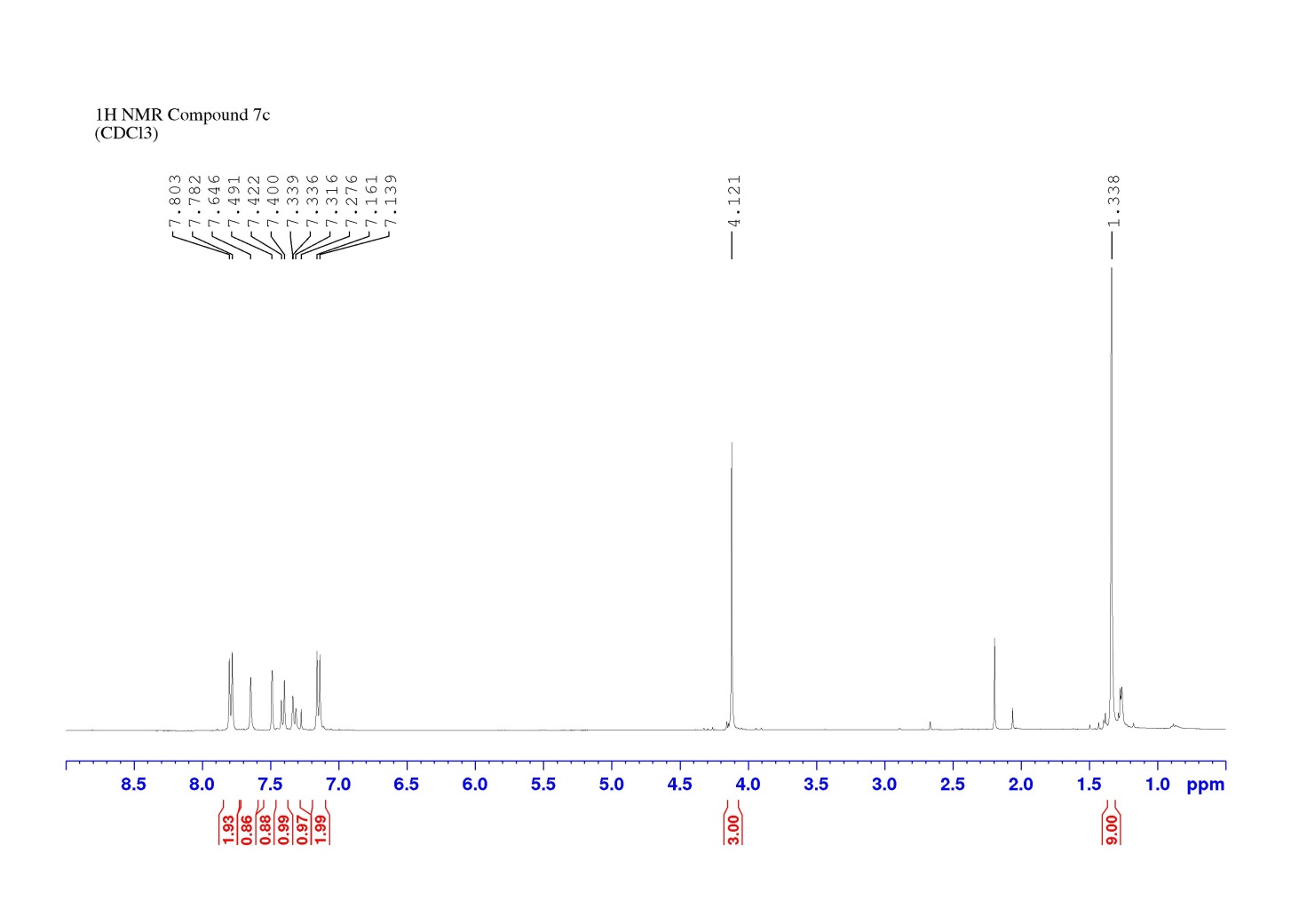
**

**
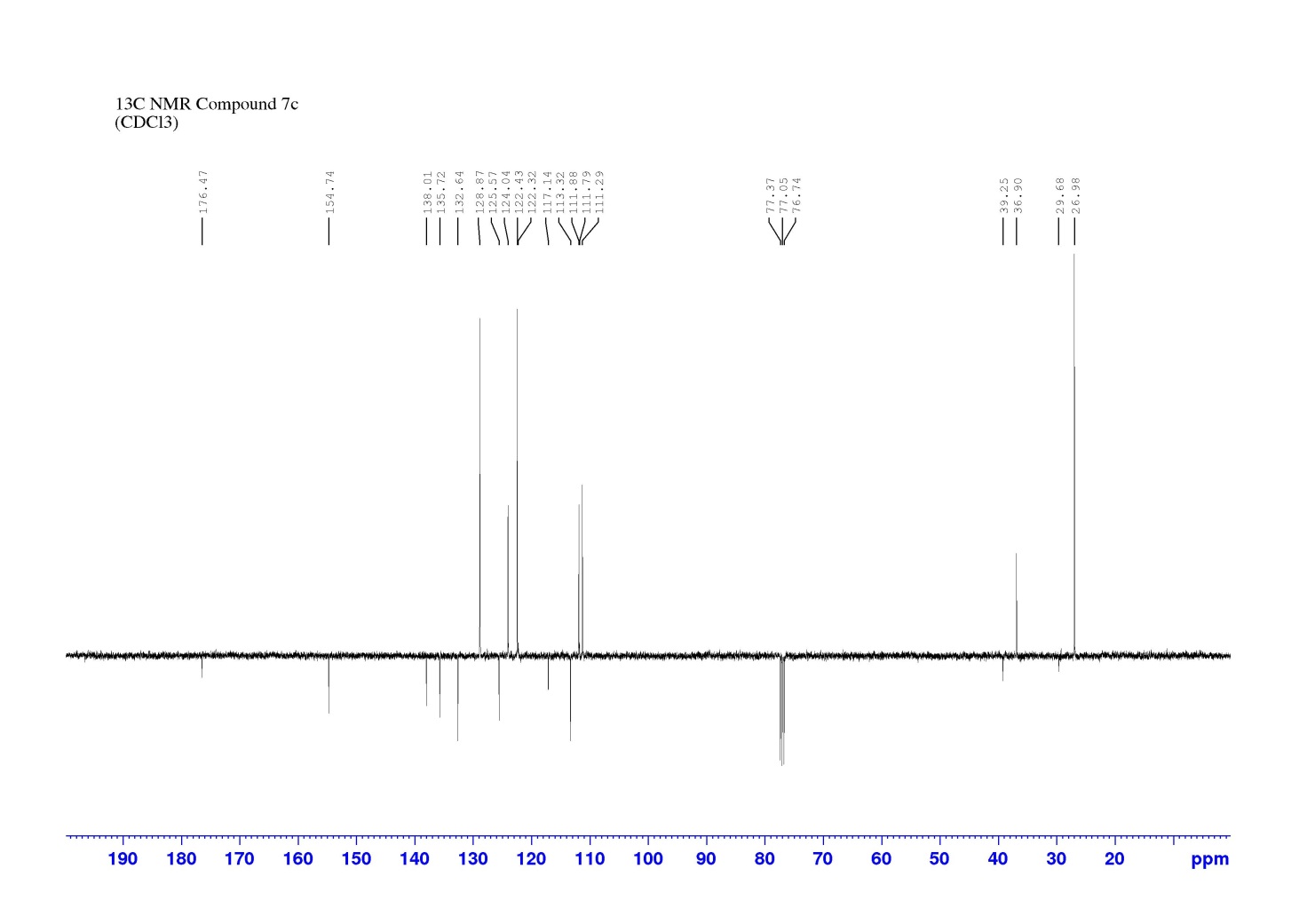
**

**
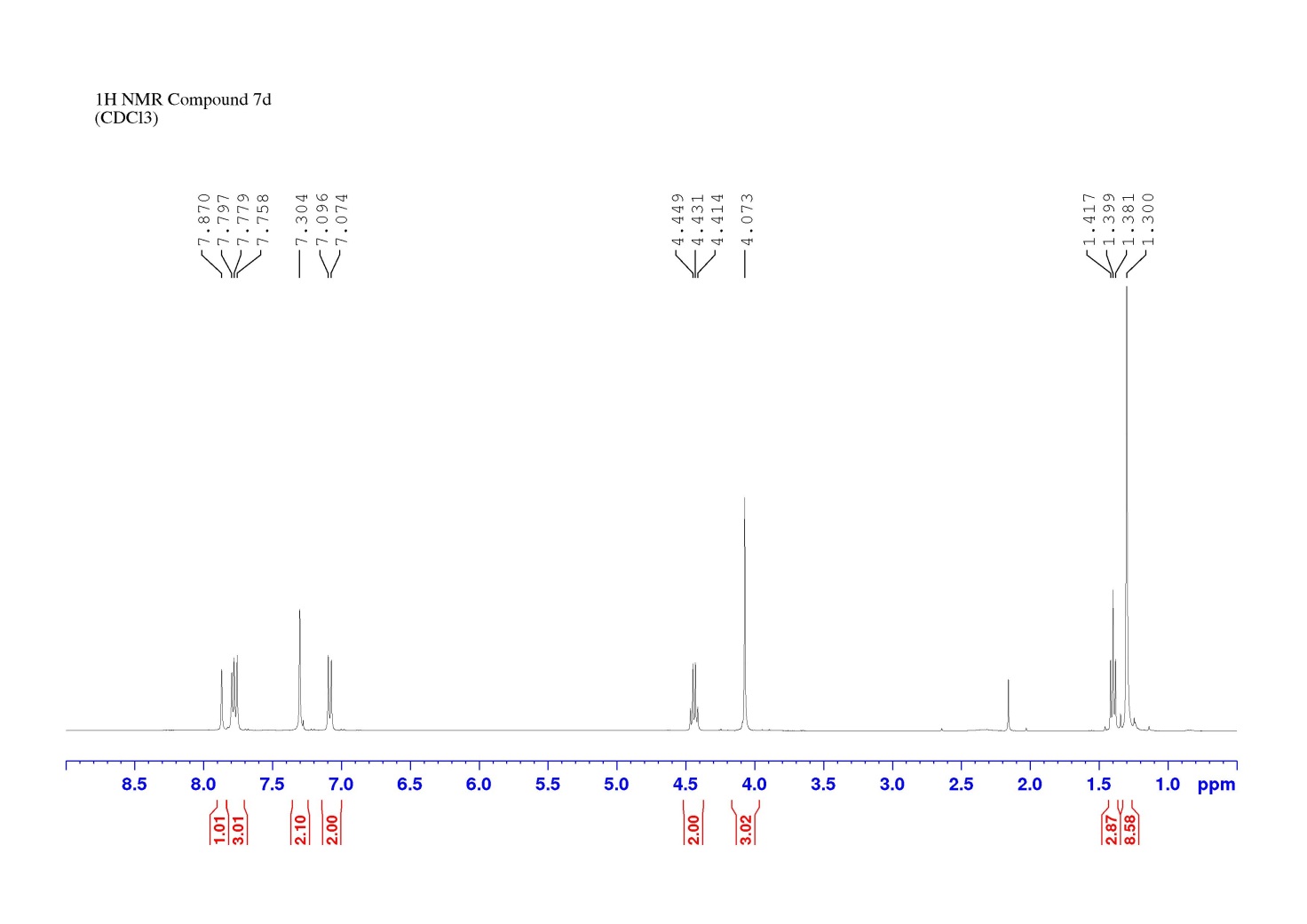
**

**
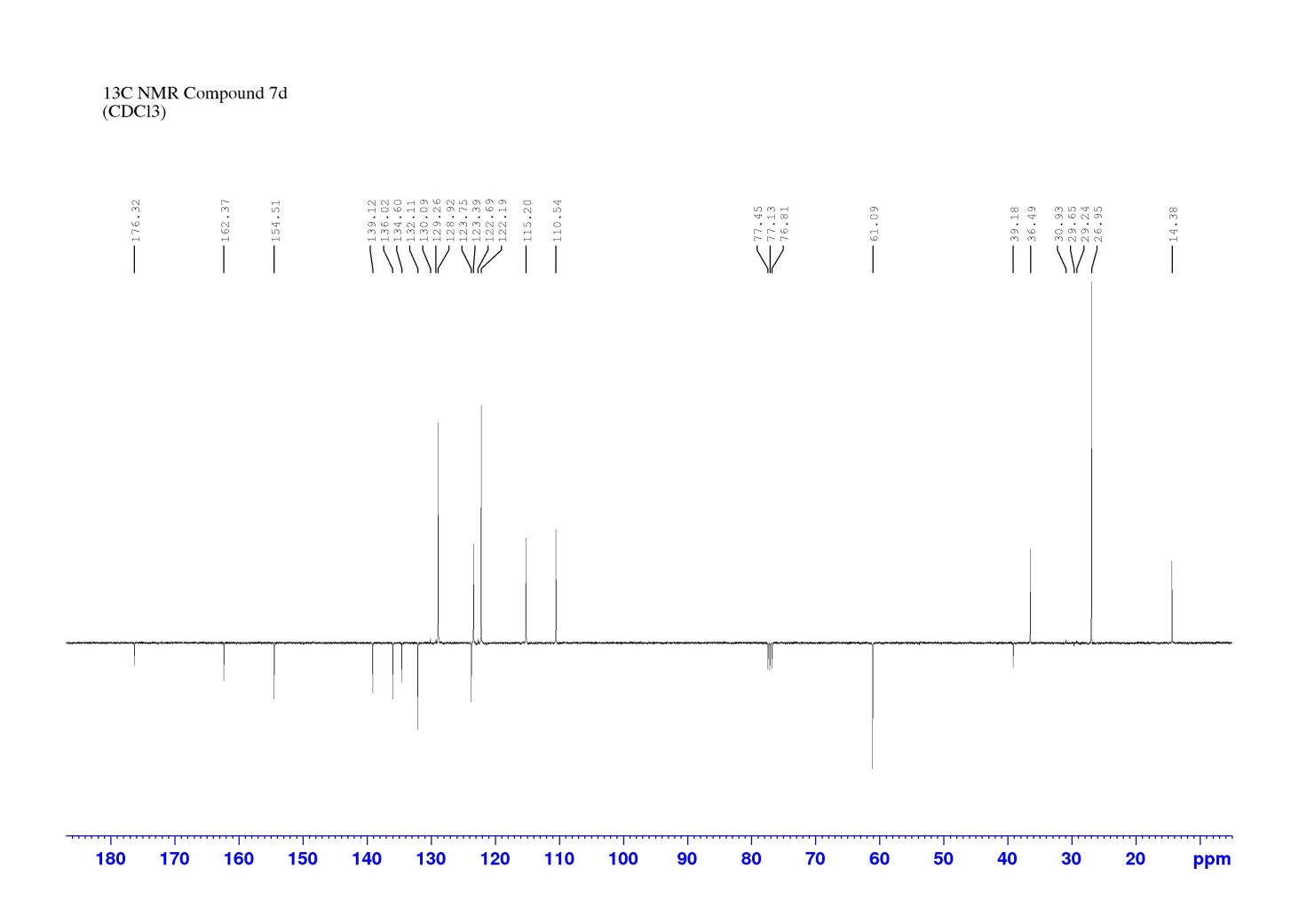
**

**
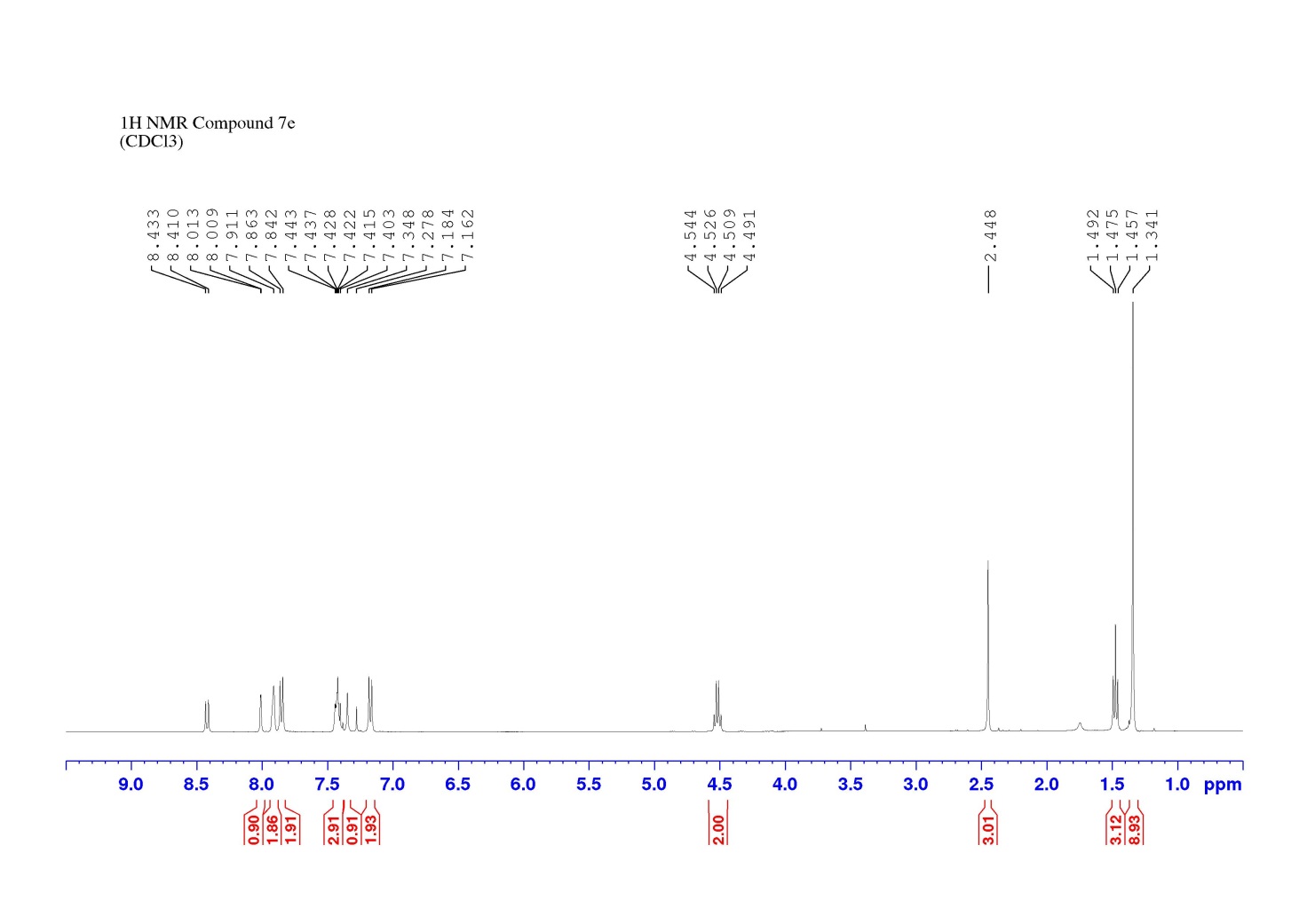
**

**
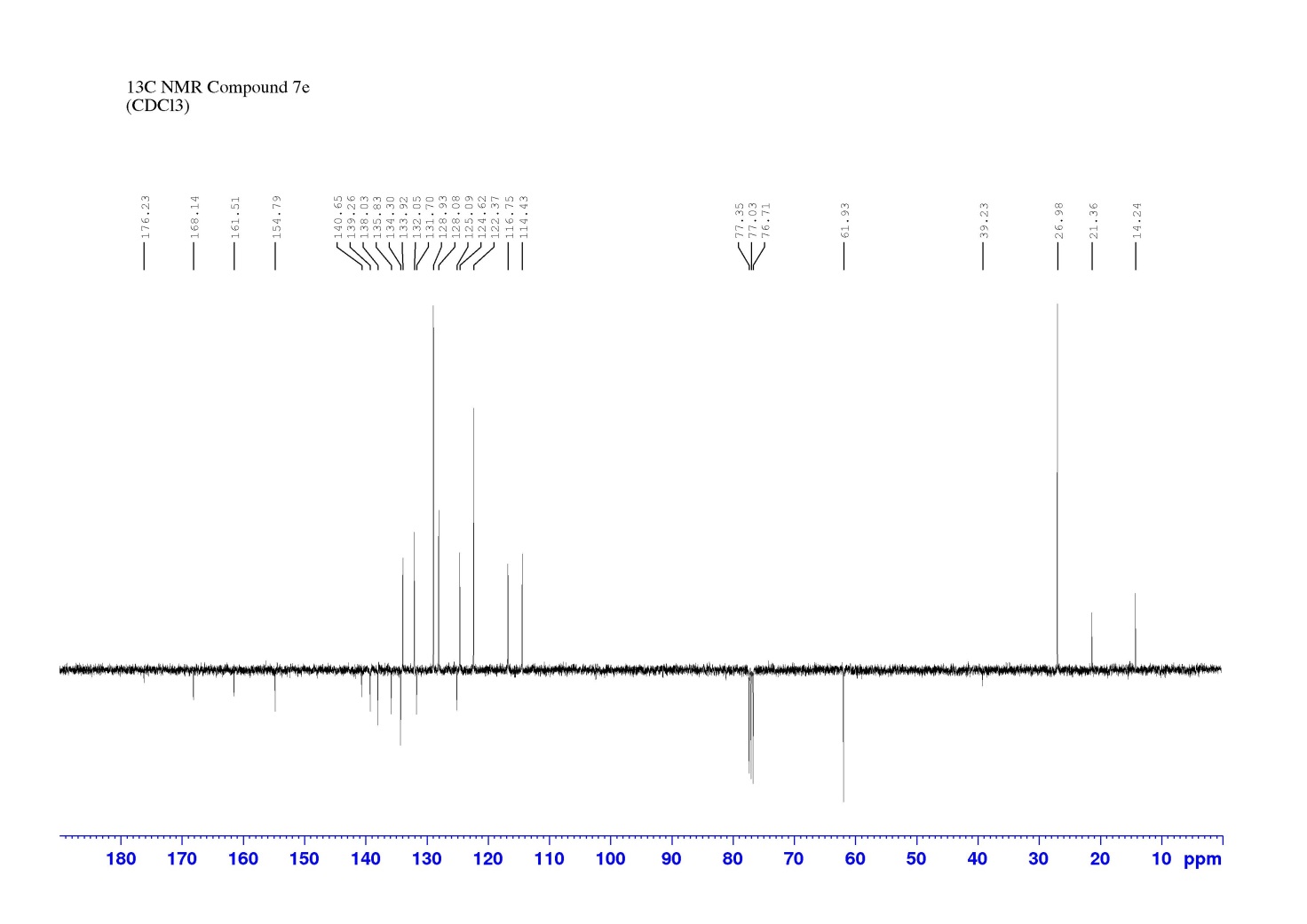
**

**
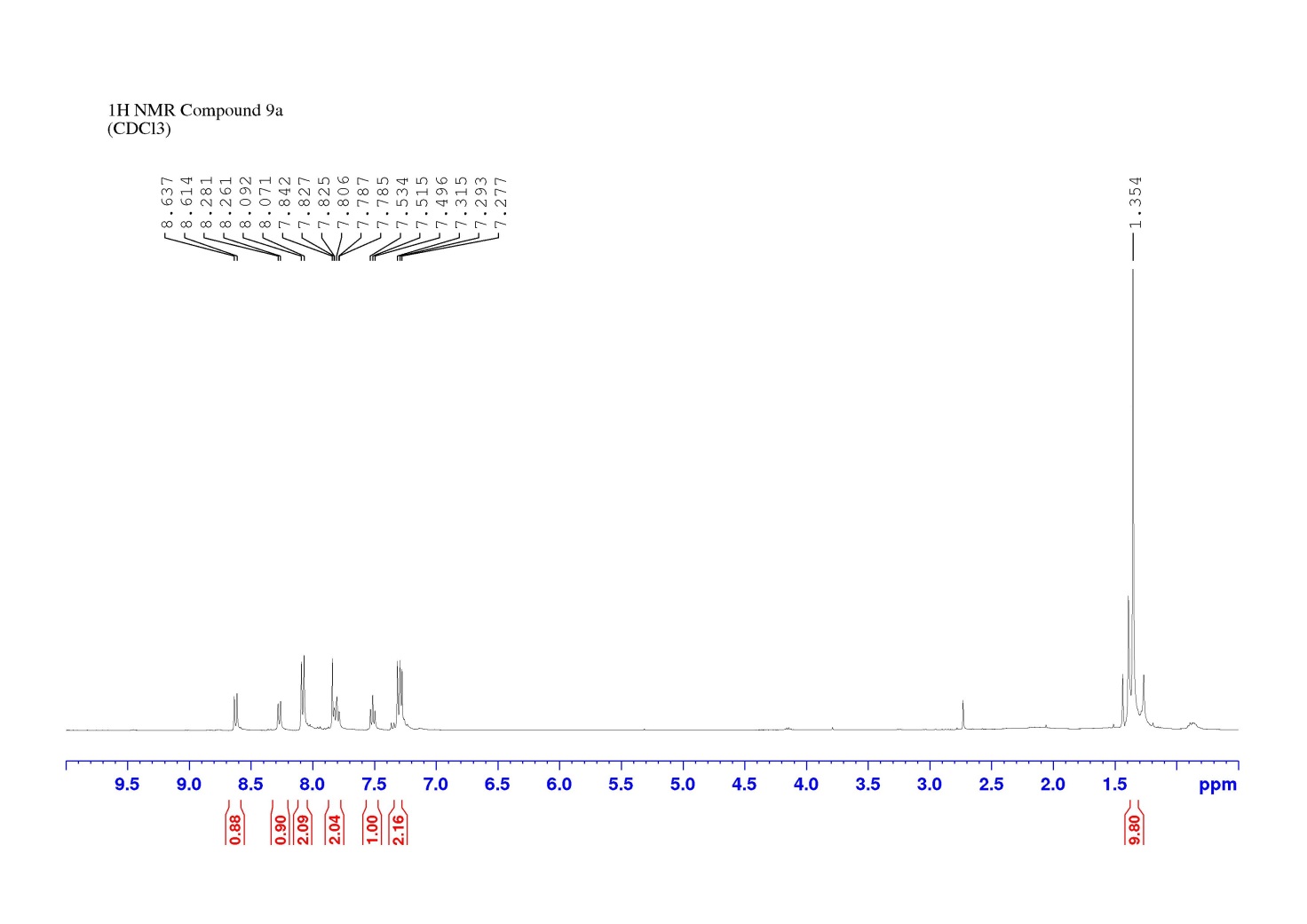
**

**
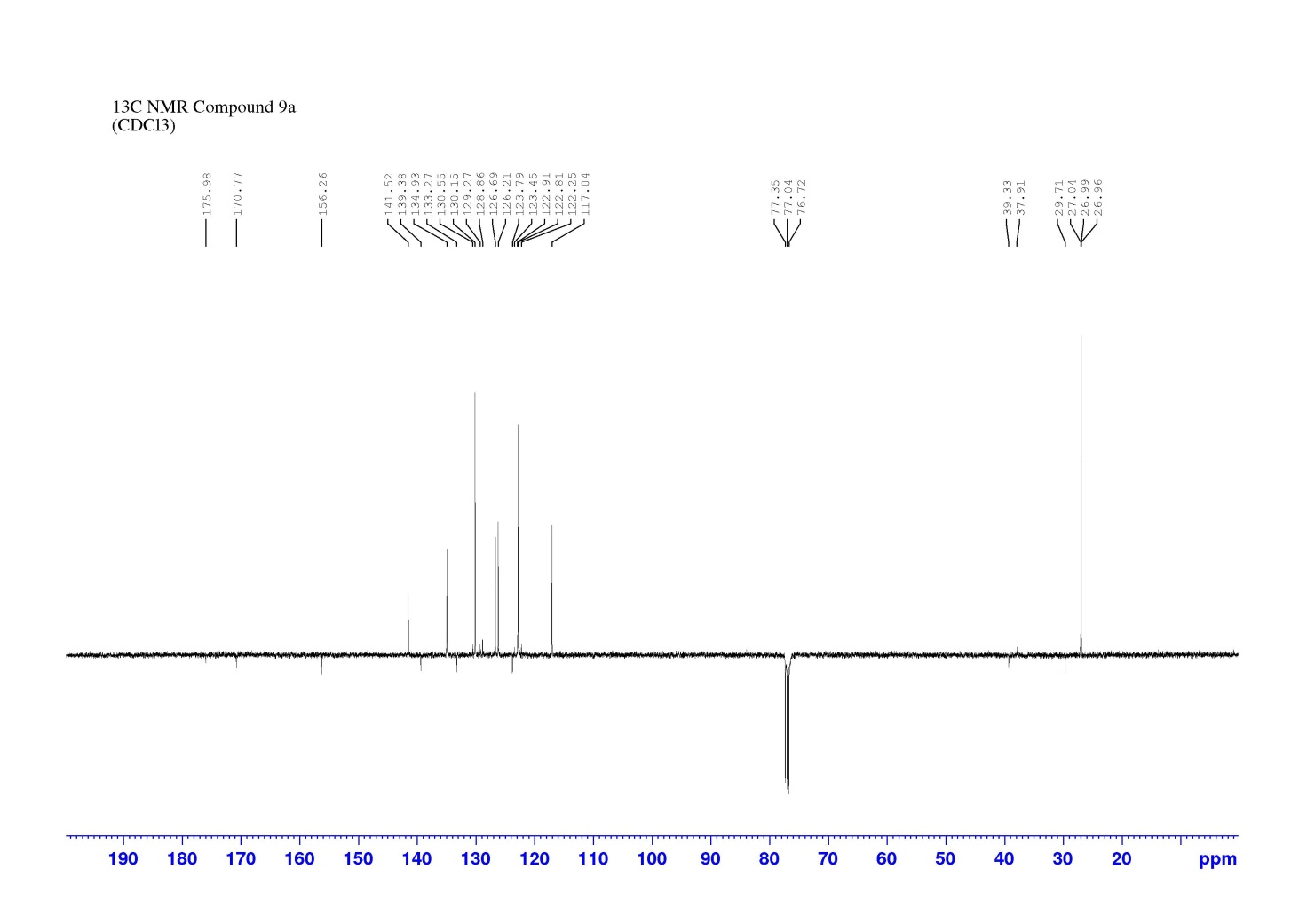
**

**
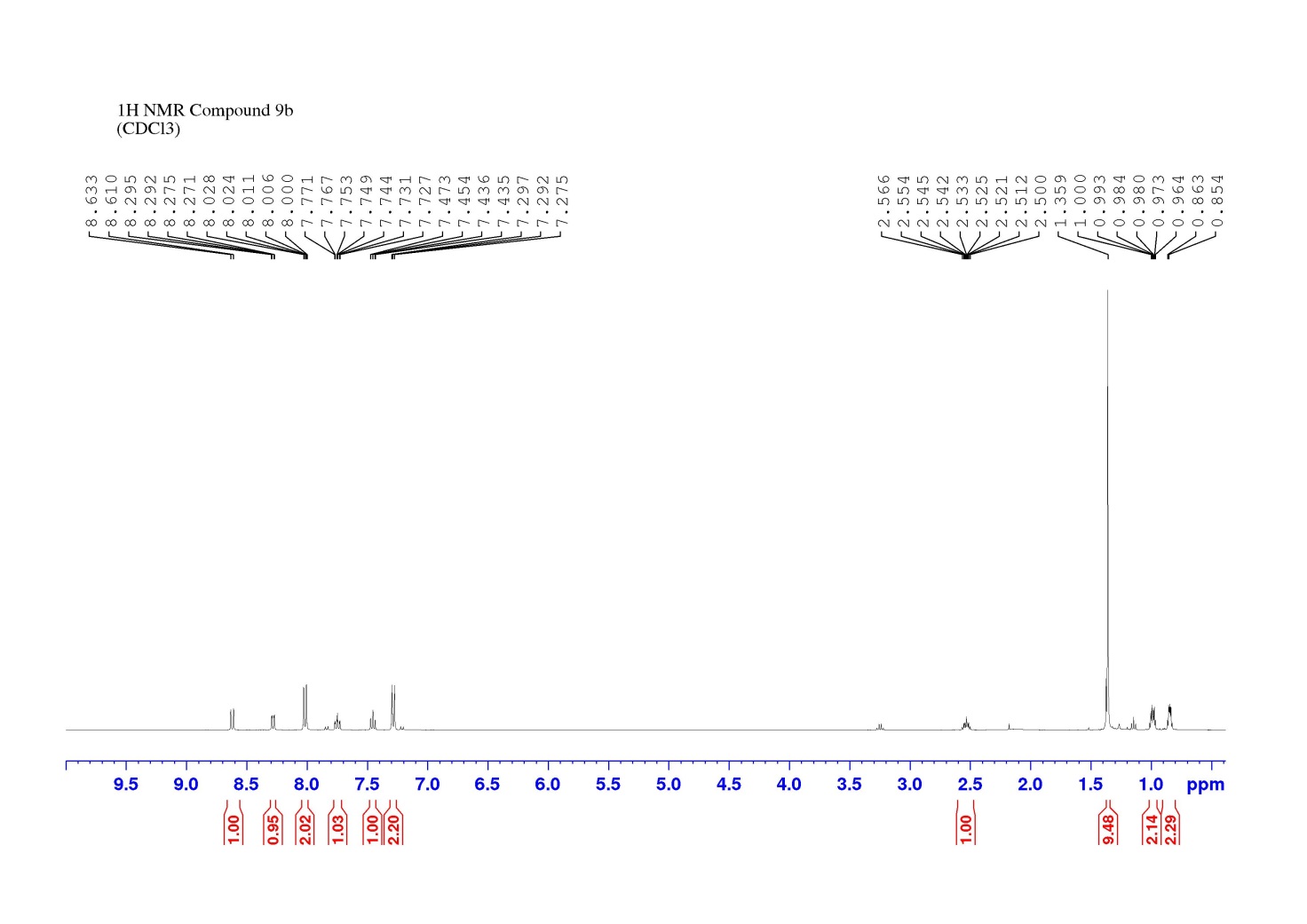
**

**
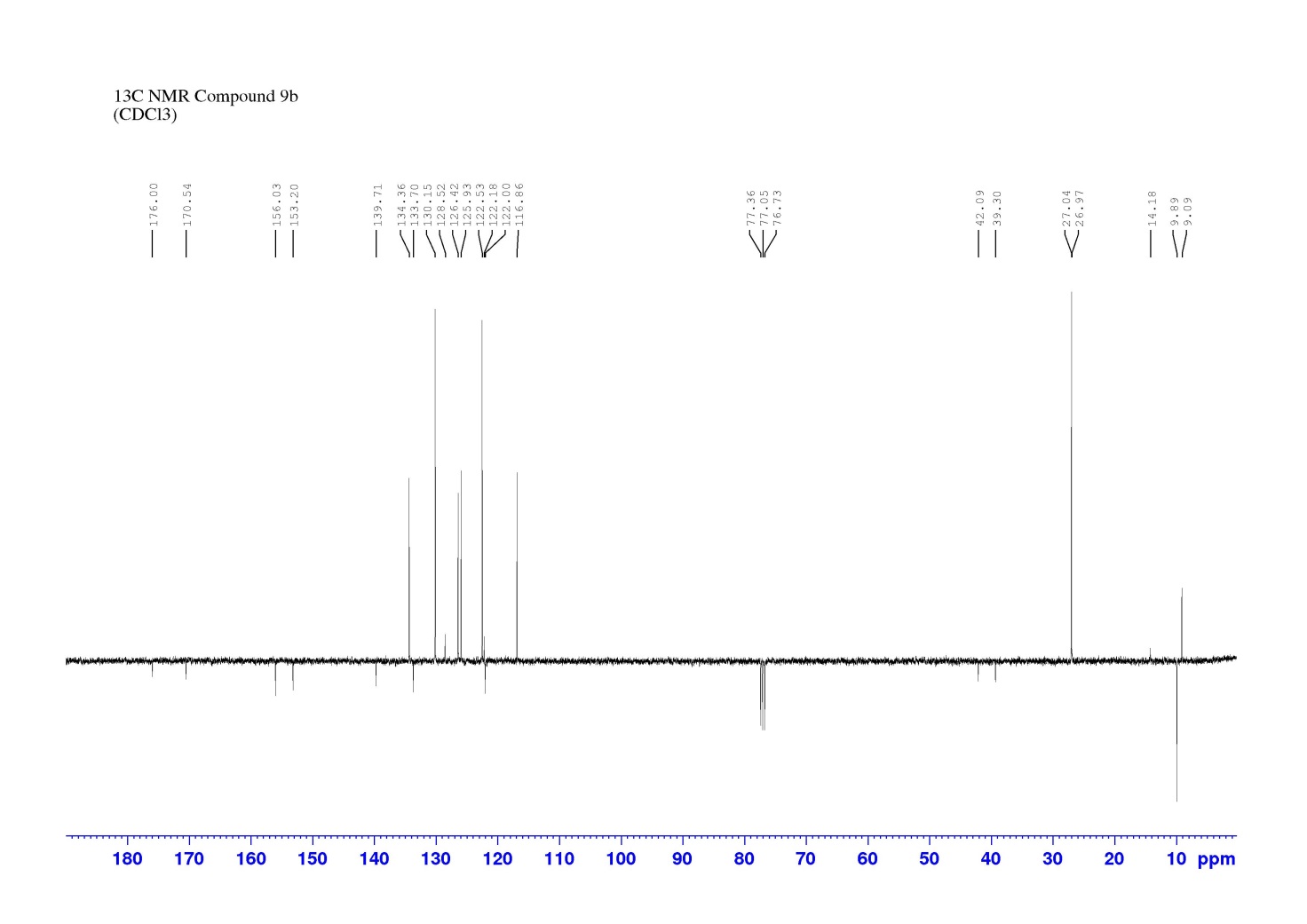
**

**
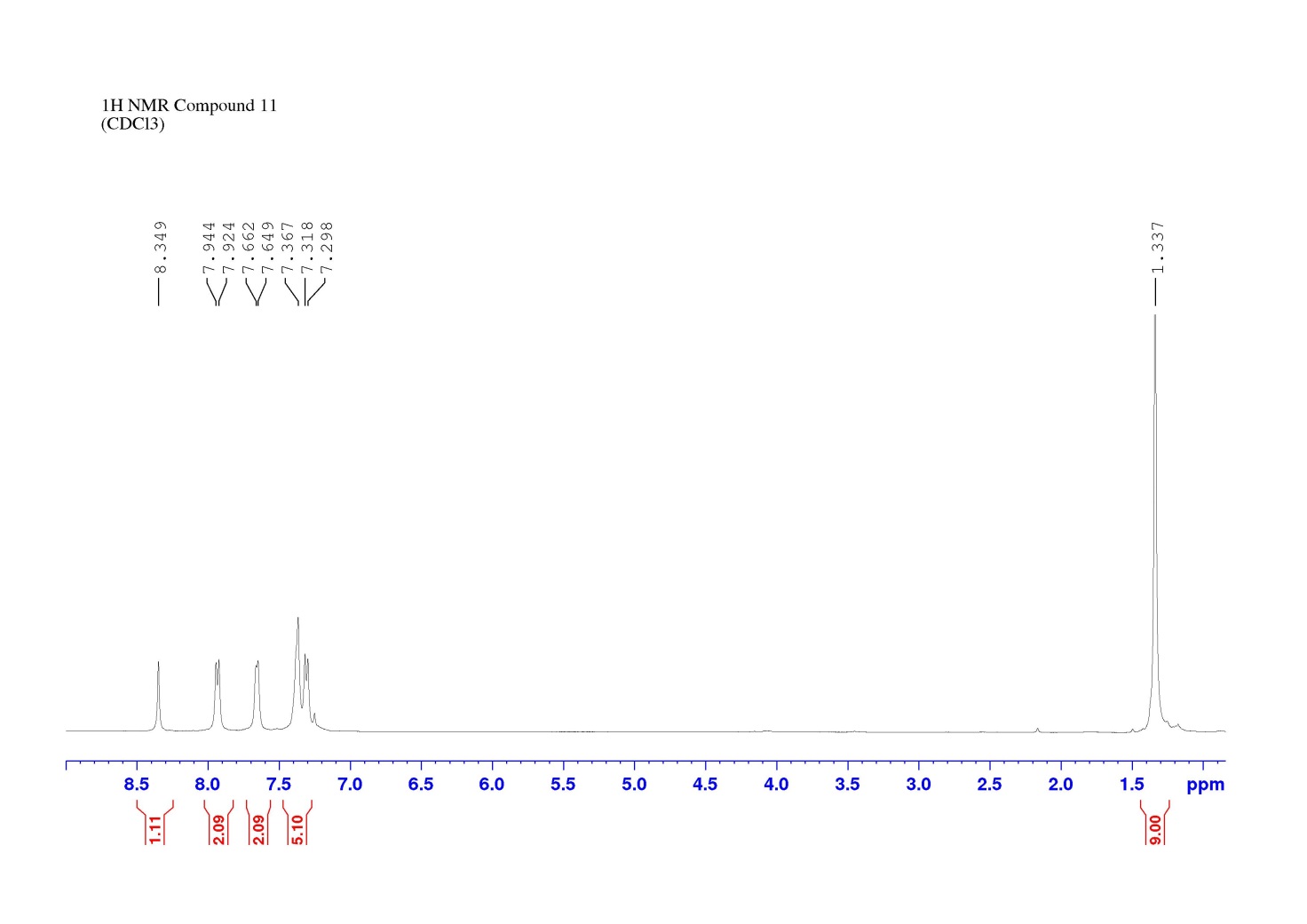
**

**
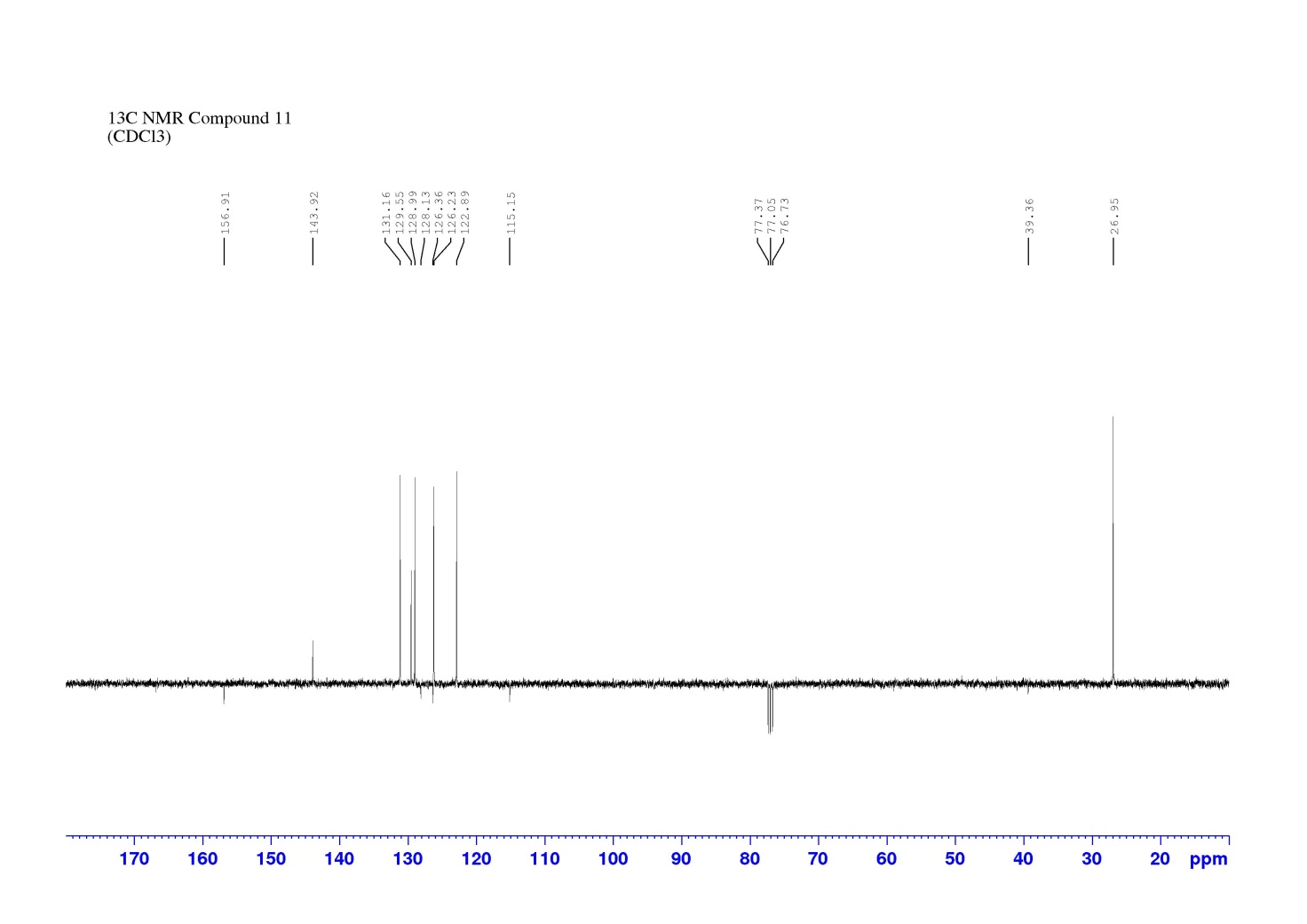
**

**Table S1. Elemental analysis**

| **Comp.** | **Formula (MW)** | **Anal. Calcd.** | | |  | **Anal. Found** | | |
| --- | --- | --- | --- | --- | --- | --- | --- | --- |
|  |  | **C** | **H** | **N** |  | **C** | **H** | **N** |
| **3a** | C_22_H_23_NO_6_S (429.49) | 61.53 | 5.40 | 3.26 |  | 61.77 | 5.42 | 3.27 |
| **3b** | C_22_H_22_N_2_O_8_S (474.48) | 55.69 | 4.67 | 5.90 |  | 55.91 | 4.68 | 5.92 |
| **3c** | C_20_H_18_N_2_O_4_S (382.43) | 62.81 | 4.74 | 7.33 |  | 62.55 | 4.72 | 7.30 |
| **3d** | C_19_H_17_N_3_O_4_S (383.42) | 59.52 | 4.47 | 10.96 |  | 59.28 | 4.45 | 10.91 |
| **3e** | C_19_H_16_N_4_O_6_S (428.42) | 53.27 | 3.76 | 13.08 |  | 53.48 | 3.77 | 13.13 |
| **3f** | C_21_H_22_N_2_O_6_S (430.48) | 58.59 | 5.15 | 6.51 |  | 58.82 | 5.17 | 6.53 |
| **3g** | C_21_H_21_N_3_O_8_S (475.47) | 53.05 | 4.45 | 8.84 |  | 52.83 | 4.43 | 8.80 |
| **3h** | C_18_H_19_N_3_O_4_S (373.43) | 57.90 | 5.13 | 11.25 |  | 57.66 | 5.11 | 11.20 |
| **3i** | C_19_H_17_N_3_O_4_S (383.42) | 59.52 | 4.47 | 10.96 |  | 59.28 | 4.45 | 10.91 |
| **3j** | C_21_H_20_N_4_O_5_S (440.47) | 57.26 | 4.58 | 12.72 |  | 57.48 | 4.59 | 12.77 |
| **3k** | C_18_H_16_F_3_N_3_O_4_S (427.40) | 50.58 | 3.77 | 9.83 |  | 50.37 | 3.75 | 9.79 |
| **3l** | C_18_H_16_N_4_O_4_S_4_ (384.41) | 56.24 | 4.20 | 14.58 |  | 56.46 | 4.21 | 14.63 |
| **4** | C_26_H_25_N_3_O_5_S (491.56) | 63.53 | 5.13 | 8.55 |  | 63.27 | 5.11 | 8.51 |
| **7a** | C_23_H_26_N_2_O_6_S (458.53) | 60.25 | 5.72 | 6.11 |  | 60.49 | 5.74 | 6.13 |
| **7b** | C_30_H_30_N_2_O_7_S (562.64) | 64.04 | 5.37 | 4.98 |  | 64.29 | 5.39 | 4.99 |
| **7c** | C_20_H_20_N_4_O_4_S (412.46) | 58.24 | 4.89 | 13.58 |  | 58.47 | 4.90 | 13.63 |
| **7d** | C_22_H_25_N_3_O_6_S (459.52) | 57.50 | 5.48 | 9.14 |  | 57.27 | 5.45 | 9.10 |
| **7e** | C_29_H_29_N_3_O_7_S (563.63) | 61.80 | 5.19 | 7.46 |  | 61.55 | 5.17 | 7.43 |
| **9a** | C_19_H_18_N_2_O_5_S (386.42) | 59.06 | 4.70 | 7.25 |  | 59.29 | 4.72 | 7.28 |
| **9b** | C_22_H_22_N_2_O_5_S (426.49) | 61.96 | 5.20 | 6.57 |  | 61.71 | 5.18 | 6.54 |
| **11** | C_20_H_19_NO_6_S (401.43) | 59.84 | 4.77 | 3.49 |  | 59.60 | 4.75 | 3.47 |


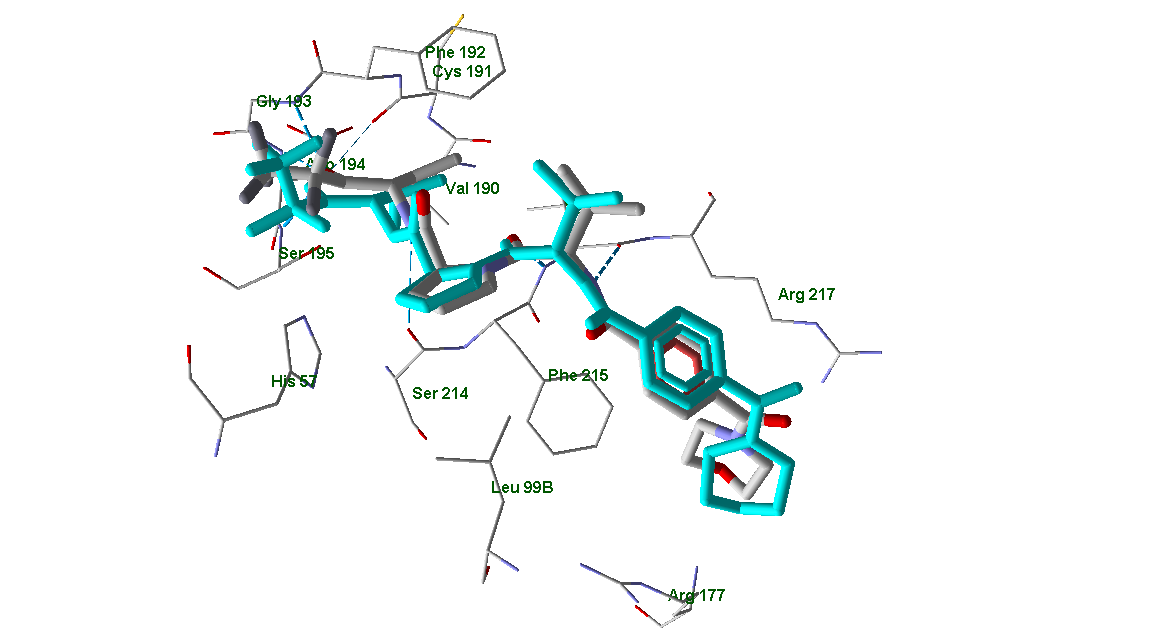


**Figure S1.** Co-crystallized SEI ligand (PDB structure 1B0F, blue) superimposed with the obtained docking pose of SEI molecule (grey skeleton). Residues within 3 Å from the co-crystallized ligand are visible.
